# *Trypanosoma cruzi* dysregulates expression profile of piRNAs in primary human cardiac fibroblasts during early infection phase

**DOI:** 10.3389/fcimb.2023.1083379

**Published:** 2023-03-02

**Authors:** Kayla J. Rayford, Ayorinde Cooley, Anthony W. Strode, Inmar Osi, Ashutosh Arun, Maria F. Lima, Smita Misra, Siddharth Pratap, Pius N. Nde

**Affiliations:** ^1^ Department of Microbiology, Immunology, and Physiology, Meharry Medical College, Nashville, TN, United States; ^2^ Biomedical Sciences, School of Medicine, City College of New York, New York, NY, United States; ^3^ School of Graduate Studies and Research, Meharry Medical College, Nashville, TN, United States; ^4^ Bioinformatics Core, School of Graduate Studies and Research, Meharry Medical College, Nashville, TN, United States

**Keywords:** Chagas disease, human cardiac fibroblasts, fibrosis, parasite pathogenesis, piRNAs, piRNome, *Trypanosoma cruzi*

## Abstract

*Trypanosoma cruzi*, the etiological agent of Chagas Disease, causes severe morbidity, mortality, and economic burden worldwide. Though originally endemic to Central and South America, globalization has led to increased parasite presence in most industrialized countries. About 40% of infected individuals will develop cardiovascular, neurological, and/or gastrointestinal pathologies. Accumulating evidence suggests that the parasite induces alterations in host gene expression profiles in order to facilitate infection and pathogenesis. The role of regulatory gene expression machinery during *T. cruzi* infection, particularly small noncoding RNAs, has yet to be elucidated. In this study, we aim to evaluate dysregulation of a class of sncRNAs called piRNAs during early phase of *T. cruzi* infection in primary human cardiac fibroblasts by RNA-Seq. We subsequently performed *in silico* analysis to predict piRNA-mRNA interactions. We validated the expression of these selected piRNAs and their targets during early parasite infection phase by stem loop qPCR and qPCR, respectively. We found about 26,496,863 clean reads (92.72%) which mapped to the human reference genome. During parasite challenge, 441 unique piRNAs were differentially expressed. Of these differentially expressed piRNAs, 29 were known and 412 were novel. In silico analysis showed several of these piRNAs were computationally predicted to target and potentially regulate expression of genes including *SMAD2, EGR1, ICAM1, CX3CL1*, and *CXCR2*, which have been implicated in parasite infection, pathogenesis, and various cardiomyopathies. Further evaluation of the function of these individual piRNAs in gene regulation and expression will enhance our understanding of early molecular mechanisms contributing to infection and pathogenesis. Our findings here suggest that piRNAs play important roles in infectious disease pathogenesis and can serve as potential biomarkers and therapeutic targets.

## 1 Introduction

*Trypanosoma cruzi* is an intracellular protozoan parasite that causes Chagas disease. This disease, which was originally endemic in Central and South America, is now present in all industrially advanced countries due modern globalization (Coura and Vinas, 2010; Gascon et al., 2010; Hotez et al., 2013; Bern et al., 2019; Bonney et al., 2019), qualifying the disease as an emerging global health problem (Tanowitz et al., 2009). Autochthonous *T. cruzi* transmissions including vertical transmission from mother to the fetus have been reported in both inland states and those sharing a border with Mexico (Dorn et al., 2007; Rassi et al., 2010; Beatty and Klotz, 2020; Lynn et al., 2020). Although the acute phase of the disease is associated with indeterminate symptoms and high parasitemia, it remains an essential prelude to the chronic condition where about 30-40% of infected individuals present with incurable cardiac, neurological, or gastrointestinal tract pathological conditions (Marin-Neto et al., 2007; Matsuda et al., 2009; Pinazo et al., 2010; Py, 2011; Martinez et al., 2019; Ricci et al., 2020). Chagas disease is the world’s leading cause of infectious myocarditis and the molecular basis of *T. cruzi*-induced Chagasic cardiomyopathy remains unknown despite ongoing research (Bonney and Engman, 2008; Rassi et al., 2009). Our laboratory among others have used several *in vitro*, *ex vivo*, human and murine cardiomyocyte culture models to show that the parasite induces an increase in the expression of extracellular matrix components during the acute phase of infection, which could lead to the cardiac remodeling observed in some chronically infected individuals (Cruz et al., 2016; Udoko et al., 2016; da Costa et al., 2019). Fibroblasts constitute an essential component of the heart where they play important roles in structure and function of the heart under normal homeostatic conditions (Souders et al., 2009; Ivey and Tallquist, 2016). Fibroblasts also play a role in the regulation of the extracellular and matricellular contents of the heart (Herum et al., 2017; Hortells et al., 2019; Silva et al., 2020). Elucidation of how the parasite dysregulates host gene expression profiles in cardiac myocytes and fibroblasts will advance our understanding of *T. cruzi* induced cardiac pathogenesis.

Accumulating literature suggests that host small noncoding RNA (sncRNA) molecules such as microRNAs (miRNAs), small interfering RNAs (siRNAs), and P-element induced wimpy testis (PIWI)-interacting RNAs (piRNAs) play important roles in regulating gene expression by forming complexes with Argonaute proteins to recognize specific target sequences (Shimoni et al., 2007; Dana et al., 2017; O'Brien et al., 2018; Rayford et al., 2021). To date, several thousand piRNAs have been identified in somatic and germ stem cells where they are thought to play important epigenetic roles including, but not limited to transposon silencing and *de novo* methylation to maintain genome integrity (Peng and Lin, 2013; Rayford et al., 2021). Dysregulated expression of piRNAs has been suggested to play important roles in tumorigenesis in a context dependent manner (Rizzo et al., 2016; AboEllail and Hata, 2017; Weng et al., 2018; Liu et al., 2019; Halajzadeh et al., 2020). In cardiac cells, it was suggested that piRNAs interact with PIWIL2 protein to facilitate cardiomyocyte proliferation and regeneration by regulating AKT signaling (Rajan et al., 2014). Patients presenting with myocardial infarction were shown to have a significantly elevated level of piR-2106027, which has been suggested to be an important diagnostic marker for the disease (Xuan et al., 2017; Das et al., 2018). Recently, we showed in infectious disease research that *T. cruzi* dysregulates the expression profile of known and novel piRNAs in primary human cardiac myocytes during the early phase of infection (Rayford et al., 2020). Our *in silico* analysis showed that significantly differentially expressed piRNAs could target regions in genes coding for *NFATC2*, *FOS*, and *TGFB1*, which are reported to be critical during the early phase of *T. cruzi* pathogenesis (Rayford et al., 2020). Despite these important functions of piRNAs, the contextual characteristics and molecular signature of the *T. cruzi*-induced piRNA profile in primary human fibroblasts (PHCF) during the early phase of infection remains unknown. Here, we challenged PHCF with the Tulahuen strain of *T. cruzi*, clone MMC20A and evaluated the piRNA expression profile (piRNome kinetics) during the early phase of infection. We validated the piRNA expression and target mRNA transcript expression. The significantly differentially expressed piRNAs were computationally predicted to target genes including *ICAM1*, *SMAD2*, *EGR1*, *CXCR2*, *FOS*, and *CX3CL1*. Furthermore, we connected them in biological interaction networks to theoretically predict piRNA pathway-level interactions involved in *T. cruzi* pathogenesis.

## 2 Materials and methods

### 2.1 Primary human cardiac fibroblast culture

PHCF were obtained from and cultured following the manufacturer’s recommendations (PromoCell, Heidelberg, Germany). Briefly, the PHCF were cultured in fibroblast basal growth medium supplemented with the supplemental mix (PromoCell, Heidelberg, Germany) containing fetal calf serum (0.05 mL/mL), recombinant human epidermal growth factor (0.5 ng/mL), recombinant human basic fibroblast growth factor (2 ng/mL) and recombinant human insulin (5 ug/mL). The cells were cultured in T75 flasks at 37°C in the presence of 5% CO_2_ to approximately 80% confluency (approximately 4 × 10^6^ cells) prior to being used in our assays.

### 2.2 Parasite culture and infection assays

Heart myoblast monolayers at 80% confluence, cultured in complete DMEM containing 5% glutamax, 10% fetal bovine serum, 1% each of penicillin/streptomycin, multivitamins and MEM non-essential amino acids (Life Technologies, Carlsbad, CA, USA), were infected with *T. cruzi* trypomastigotes. Pure cultures of highly invasive *T. cruzi* trypomastigotes (clone MMC 20A, Tulahuen strain) were harvested from the supernatant of infected heart myoblast monolayers as previously described (Lima and Villalta, 1989; Villalta et al., 1990). The parasites were washed with Hanks Balanced Salt Solution (HBSS) and resuspended in PHCF growth medium without supplement at 1 × 10^7^ parasites/mL. For the infection assays, confluent PHCF monolayers (about 80%) were starved in HBSS containing 30 mM HEPES, followed by the addition of *T. cruzi* trypomastigotes in PHCF growth medium without supplements at a ratio of 10 parasites per cell. Parasite-challenged PHCFs were incubated for 1, 3, and 6 h, respectively, in triplicate for the assays. Total and small RNAs were purified from the samples. Mock-infected (basal media only) PHCFs served as control.

### 2.3 RNA extraction and quality assessment

Control and parasite-challenged PHCF were washed with HBSS. The cells were lysed in QIAzol, an RNA extraction lysis buffer and extracted with chloroform following the manufacturer’s instructions (Qiagen, Valencia, CA, USA). The aqueous phase of the extract was mixed with an equal volume of 70% ethanol and passed through the RNeasy Mini spin column. The eluate, which contained small RNA species, was mixed with 0.65 volumes of pure ethanol and passed through an RNeasy MiniElute spin column. The column was washed with 80% ethanol and bound small RNA species were eluted with RNase-free water essentially as described by the manufacturer (Qiagen). Large RNAs bound to the RNeasy mini spin column were washed and eluted with RNase-free water as described by the manufacturer (Qiagen). The quality of the purified RNA was analyzed using the Bioanalyzer 2100 system (Agilent Technologies, Santa Clara, CA, USA) to determine the RNA Integrity Number (RIN). Samples with a RIN of at least 8 were considered for further analysis.

### 2.4 RNA-sequencing of sncRNA, filtering, and expression evaluation

Briefly, purified small RNA species ranging from 18 to 30 nt were ligated to the Illumina 3′ adaptor and 5′ adaptor. Ligation products were gel-purified, reverse-transcribed and amplified by Rolling-Circle Replication (RCR). This linear amplification only copies the original DNA template instead of copy-of-a-copy in order to make small, very high-density sequencing compacted templates called DNA NanoBalls (DNBs). The DNBs were compacted on high-density patterned nanoarray and sequenced by combinatorial Probe-Anchor Synthesis (cPAS), a sequencing chemistry technique which is optimized for DNBseq. The combination of linear amplification and DNB technology reduces the error rate while enhancing the signal. Smaller DNB spots with highly concentrated DNA deliver significantly higher dye density than PCR cluster arrays, leading to higher signal-to-noise for optimal imaging signal integrity. High-throughput sequencing (HTS) was conducted using the BGISEQ-500 sequencing platform for small RNA (BGI, Cambridge, MA, USA) utilizing the NGS 2.0 DNBseq technology. The DNBseq sequencing technology (NGS 2.0) combines the power of DNA Nanoballs (DNB), PCR-free Rolling Circle Replication, Patterned Nano Arrays and cPAS to deliver a new level of data clarity and reliability. The BGISEQ-500 sequencing platform has comparable sensitivity and accuracy in terms of quantification of gene expression, and low technical variability as compared to the Illumina HiSeq platform (Fehlmann et al., 2016; Natarajan et al., 2019). High-throughput sequencing (HTS) was conducted using the BGISEQ-500 sequencing platform for small RNA (BGI, Cambridge, MA, USA) (Drmanac et al., 2010; Fu et al., 2018). In total, 29,496,864 raw HTS data were filtered by eliminating low-quality reads (less than 20 in Phred quality score), as well as removing confounders such as adaptors and other contaminants including missing 3′ primers, 5′ primer contamination and incomplete small non-coding RNA (sncRNA) reads of less than 18 bp. Reads that met our analysis criteria were subjected to size filtration for piRNA to select for sncRNA transcripts that were 25–30 bp in length and did not match any known miRNA or siRNA sequences. Bowtie was used to map the reads to reference genomes (Langmead et al., 2009). The piRNA annotation program (Piano) was used to predict known piRNAs *via* a support vector machine (SVM) algorithm with transposon interaction informatics (Wang et al., 2014). piRNA expression was determined using the standard transcripts per kilobase million mapped (TPM) method (John et al., 2004).

### 2.5 Analysis of differential piRNA expression

The NOISeq method (version 3.34) was used to determine differentially expressed piRNAs. Each time point was screened for significantly different piRNA expression compared to control by calculating the log_2_ fold-change (M) and absolute differential value (D) between each pair of time points to build a noise distribution model. A piRNA is counted as differentially expressed if M and D values are likely to be higher than in noise. The significance threshold for differential piRNA expression was set to fold-change ≥2 or ≤2 and a divergence probability ≥0.8. A probability of 0.8 is equivalent to an odds value of 4:1, meaning that a given piRNA is 4 times more likely to be differentially expressed than non-differentially expressed (Tarazona et al., 2011). NOISeq calculates probability of differential expression (q-value) rather than FDR for multiple comparisons correction. This statistic is considered comparable to the Benjamini and Hochberg FDR as explained in Efron et al. (Efron et al., 2001).

### 2.6 piRNA target prediction

Differentially expressed piRNAs were compared against hg38 RefSeq transcripts in miRanda (Enright et al., 2003) using a high-stringency pairing score cutoff of ≥175, an energy cutoff of ≤−30 kcal/mol and a requirement for exact seed region alignment.

### 2.7 Quantitation of mRNA and piRNA expression

Total RNA from *T. cruzi* challenged PHCF was reverse transcribed into cDNA using iScript cDNA synthesis Kit (Bio-Rad, Hercules, CA, USA). Quantitative real-time PCR (qPCR) for validation of the mRNA targets was done using a customized PrimePCR assay (Bio-Rad, Hercules, CA, USA) containing the genes of interest. In the PrimePCR array experiment, total RNA (1 µg) converted to cDNA using the iScript cDNA synthesis kit was mixed with SsoAdvanced SYBR green 2X master mix and loaded (20 µl/per well) on a customized PrimePCR plate containing primers for selected genes to be validated, including housekeeping genes and controls as described (www.bio-rad.com/PrimePCR). The PCR amplification was carried out on a C1000 Touch Thermal Cycler as described by the manufacturer (Bio-Rad, Hercules, CA, USA). The Ct values of housekeeping genes (beta-2 microglobulin, Ribosomal protein, lateral stalk subunit (RPLO) and, beta-actin) were checked for consistency on all plates across all samples. The data were normalized against the housekeeping genes and control samples using CFX manager analysis software with support from Bio-Rad technical service using the ΔΔCT method. The primer sequences used for quantification of the transcript levels in the PrimePCR were not disclosed by Bio-Rad.

For piRNA quantification, we used the TaqMan Small RNA Assay essentially as described by the manufacturer (Thermofisher, Waltham, MA, USA). Briefly, piRNA-specific stem-loop RT primers were used to create each cDNA template. The TaqMan Small RNA Assay used (assay ID) were as follows: novel_piR_17 (CTNKRW3), hsa_piR_016828 (CTH497C); hsa_piR_017716 (CTGZFME); hsa_piR_016742 (CTFVK2G); novel_piR_167 (CTPRKGZ). For each piRNA, a TaqMan primer-probe set was utilized to amplify each target cDNA by TaqMan qPCR, using U6 as a housekeeping sncRNA. TaqMan real-time PCR assays were carried out on a CFX96 Thermal Cycler (Bio-Rad) as per Thermofisher recommendations. The Ct values of housekeeping gene U6 were checked for consistency on all plates across all samples. The data were normalized against the housekeeping U6 and control samples using CFX manager analysis software using the ΔΔCT method. The primer sequences used to quantify the piRNA levels in the TaqMan Small RNA were not disclosed by Thermofisher.

### 2.8 Target gene selection mapping of biological pathway interactions

To identify piRNA target genes to evaluate with an unbiased approach, we used the Genshot search engine (Lachmann et al., 2019) (https://maayanlab.cloud/geneshot/). Geneshot produces a ranked list of genes most relevant to user-provided search terms. These lists integrate information from publications and gene expression data to identify both common and understudied genes. We used the terms “cruzi” and “fibrosis” to create a prioritized list which was matched with the piRNA target list. The resulting genes were used as input for network construction. Biological interaction network construction was conducted with the GeneMANIA algorithm (Warde-Farley et al., 2010) by querying multiple biological interaction databases including GEO, BioGRID and EMBL-EBI. Predicted piRNA target genes were set as starting seed nodes, and the network was then expanded to one degree of biological interaction using GeneMANIA and visualized with Gephi (Mathieu Bastian and Jacomy., 2009) (https://gephi.org/).

### 2.9 Data availability

All relevant data not presented in the manuscript are located in the SRA database: SRA accession: SRX12127485

## 3 Results

### 3.1 piRNAs are differentially expressed in PHCF during early phase of *T. cruzi* infection

**Figure 1:**
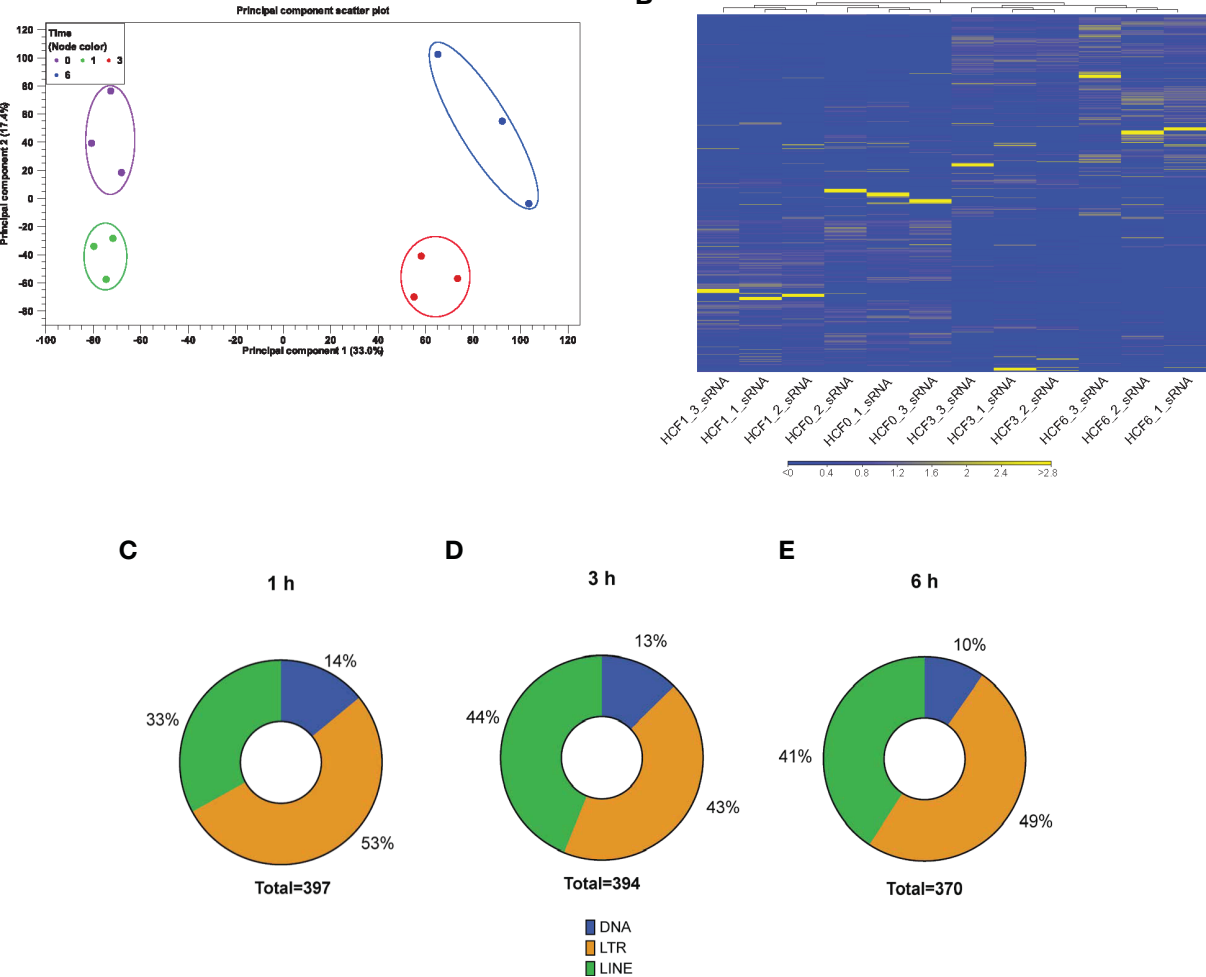
RNA-seq analysis of small RNAs dysregulated by *T. cruzi* infection of PHCF. **(A)** Principle component analysis of PHCF gene expression was completed for all samples and probe sets. The x-axis is PC1 while the y-axis is PC2, with the percentage of variance indicated in parenthesis. PHCF was challenged with *T. cruzi* for various time points (0, 1, 3, and 6) denoted by circle and clustering (0 h = purple, 1 h = green, 3 h = red, 6 h = blue). **(B)** Heatmap and hierarchical clustering was performed on control and test samples 0, 1, 3, and 6 h, respectively, using Euclidean distance measure and single linkage analysis. Each column represents one sample and each row represents one small non-coding RNA within the data set. **(C–E)** Differential expressed putative and known piRNAs induced during parasite challenge of PHCF at 1, 3, and 6 hours respectively, belong to various transposable element subfamilies.

To evaluate if *T. cruzi* can dysregulate piRNA expression in heart cells, we challenged PHCF with invasive *T. cruzi* trypomastigotes Tulahuen strain clone MMC20A. Small RNAs were purified and sent to BGI (Cambridge, MA, USA) for sequencing, as previously described (Rayford et al., 2020). To evaluate the clustering and variance between biological replicates, we performed principal component analysis. The analytical output showed segregation of 0, 1, 3, and 6 hour time points in the PCA scatter plot. Hierarchical clustering and heatmap show *T. cruzi* challenged samples clustered independently of the control group. Principal component 1 (33.0%) and principal component 2 (17.4%) showed clustering of each replicate group (**Figure 1A**). Furthermore, to evaluate whether the gene expression profiles of sncRNAs at each time point was specific, we generated hierarchical clustering, which showed that biological replicates of each time point clustered together (**Figure 1B**).

Others suggested that piRNAs can be generated from transposable element (TE) sequences to target and repress TEs, therefore, piRNAs can be classified into TE families (Weick and Miska, 2014; Nandi et al., 2016; Wang and Lin, 2021). We evaluated the origin of the differentially expressed (DE) piRNAs and mapped them to their various genomic loci such as DNA, long interspersed nuclear elements (LINE), and long terminal repeat (LTR) TE families; we found that during the early phase of *T. cruzi* infection of PHCF, the majority of DE piRNAs were derived from LTRs, representing 53% at 1 h, 43% at 3 h, and 49% at 6 h, respectively (**Figures 1C–E**).

### 3.2 *T. cruzi* induces differential expression of known and novel piRNAs in PHCF during the early phase of infection

**Figure 2:**
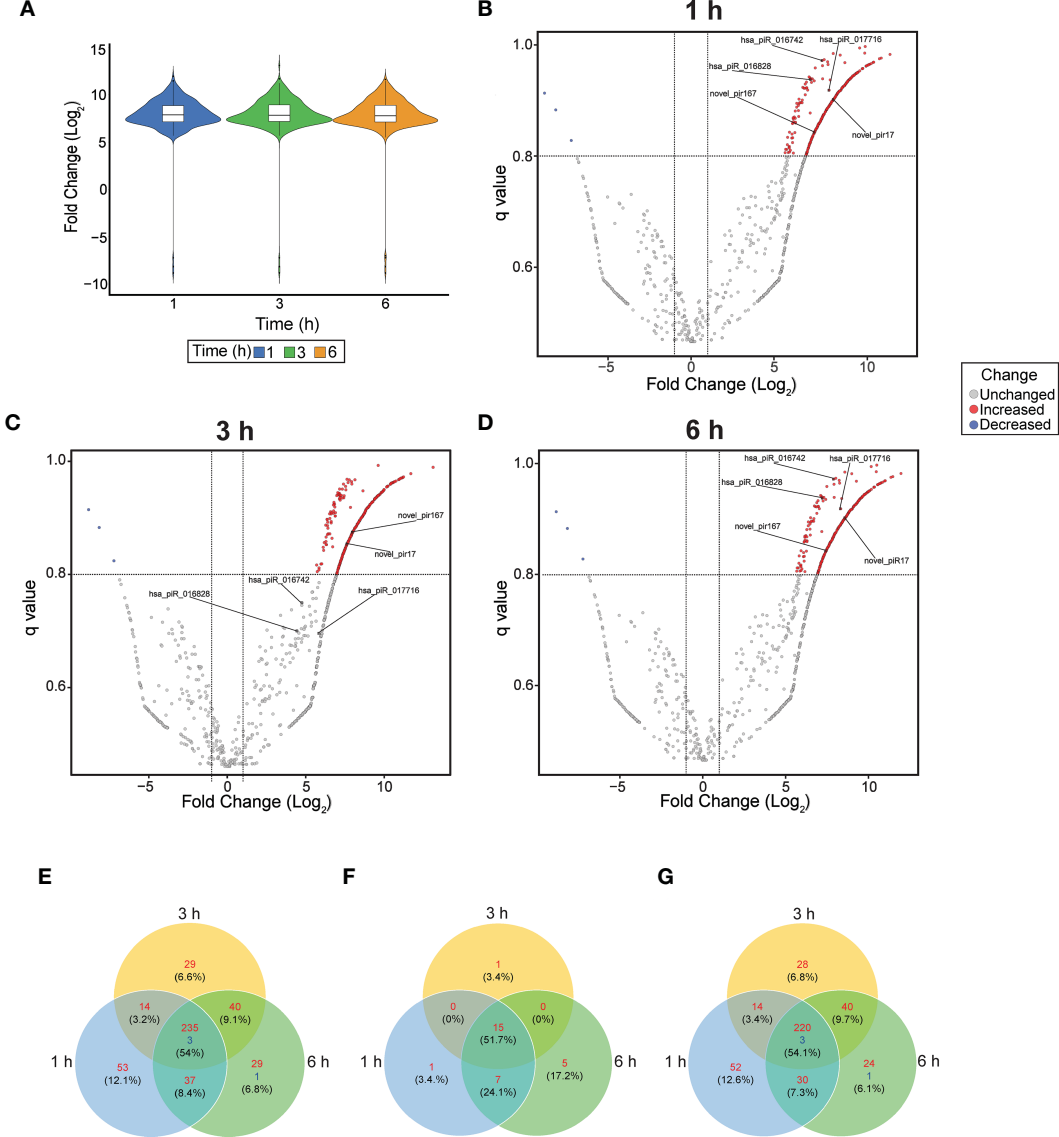
Dysregulation of piRNA expression in during early *T. cruzi* infection of PHCF. **(A)** Violin Plot of differentially expressed piRNA at each time point. The boxplot indicates the median, quartiles, and extremes of piRNA fold change. The width of the colored areas represents the proportion of piRNAs exhibiting a given level of expression. Volcano plots of piRNA expression at 1 **(B)**, 3 **(C)**, and 6 **(D)** hours post-*T. cruzi* challenge. The x-axis indicates the log_2_Fold change relative to the 0-hour control. The y-axis shows the q value. Upregulated and downregulated piRNAs are shown in red and blue, respectively. The criteria for statistically significant differential expression was log_2_Fold change >1, q >.8, and are indicated by the x-intercept and y-intercepts. The 5 selected piRNAs evaluated in our study are labeled in each plot. **(E)** Venn diagram of all upregulated (red) and downregulated (blue) piRNAs at various time points after *T. cruzi* challenge with FDR < 0.05. **(F)** Venn diagram of known piRNAs and **(G)** putative piRNAs dysregulated over the course of infection. These piRNAs previously not published have been deposited into NCBI SRA database.

To evaluate significant changes in the piRNA expression profile of PHCF during parasite infection, we used NOISeq to analyze normalized DE (Tarazona et al., 2015). Violin plot (**Figure 2A**) and volcano plots (**Figures 2B–D**) were used to visualize and identify DE across the various time points during parasite challenge. We found that 441 piRNAs were significantly DE during our experimental parasite challenge. Of these, 235 piRNAs were upregulated throughout the course of infection, while 3 were downregulated when compared to control (**Figure 2E**). **Supplemental Tables 1**, **2** show statistical significance for each DE piRNA and sequence, respectively. Of the piRNAs we found to be dysregulated, a total of 29 known piRNAs were upregulated. Fifteen (51.7%), were upregulated at all the time points (**Figure 2F**). The majority of the dysregulated piRNAs during early *T. cruzi* infection of PHCF were putative and novel; 220 were upregulated and 3 were downregulated at all time points relative to control (**Figure 2G**). Putative piRNAs accounted for all the downregulated piRNAs. The greatest count of DE putative piRNAs was 52 at 1h compared to 28 at 3 h, and 25 at 6 h, respectively.

### 3.3 Parasite induced DE piRNAs were computationally predicted to target fibrogenic and inflammatory genes

Accumulating evidence from our laboratory and others suggest that *T. cruzi* alters host cell expression profiles during the course of infection. In particular, we and others showed that the parasite dysregulates the expression profile of profibrotic and inflammatory molecules at both the transcript and protein levels through unknown mechanisms (Udoko et al., 2016; Suman et al., 2018).

To determine if any of the DE piRNAs could potentially target any of the reported fibrotic and inflammatory genes, we used the miRANDA algorithm (Enright et al., 2003) to computationally predict potential target binding sites. We then used Geneshot (Lachmann et al., 2019) to obtain a ranked list of genes correlating with search terms “cruzi” and “fibrosis.” We used the overlap of the prioritized gene list and piRNA targets to identify genes for further analysis including *ICAM1*, *SMAD2*, *EGR1*, *CX3CL1*, and *CXCR2*.

**Figure 3:**
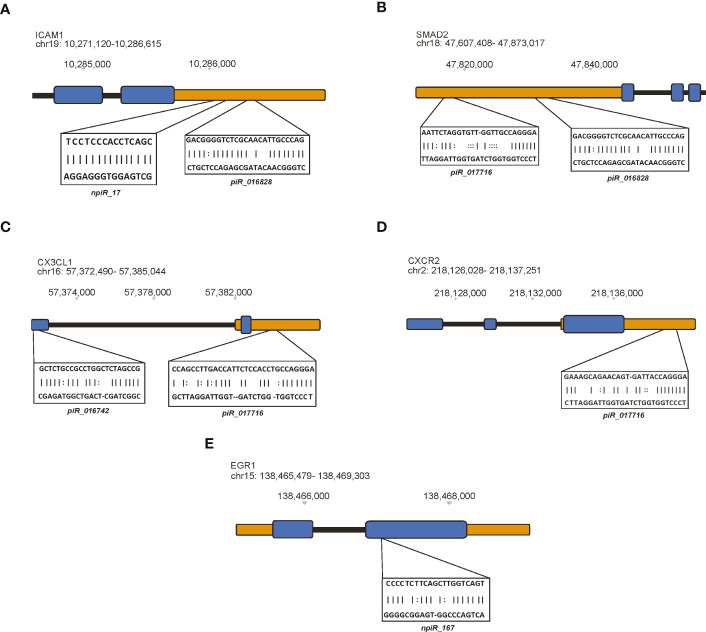
Differentially expressed piRNAs dysregulated by *T. cruzi* infection map to specific genetic regions. The miRanda algorithm was used to predict potential target binding sites of both known and putative piRNAs. In these cartoons, exons represented by blue blocks, 5’ and 3’ untranslated regions (UTRs) are orange, and intronic regions are black. piRNAs predicted to target **(A)** ICAM1, **(B)** SMAD2, **(C)** CX3CL1, **(D)** CXCR2, and **(E)** EGR1.

Few studies have investigated the expression or presence of ICAM1 during parasite infection (Cunningham et al., 2017; Tuikue Ndam et al., 2017). However, several research findings have implicated ICAM1 in various fibrogenic diseases (Kuwahara et al., 2003; Tsoutsou et al., 2004; Richards et al., 2012; Pincha et al., 2018; Wen et al., 2022). Putative npiR_17 and known piR_016828 were predicted to target the 3’ UTR region of *ICAM1* (**Figure 3A**). We and others have reported that the TGF-β signaling pathway plays an important role in *T. cruzi* infection, pathogenesis and, fibrogenesis (Waghabi et al., 2005; Ferrao et al., 2015; da Costa et al., 2019). Our *in silico* analysis showed that two known piRNAs, piR_017716 and piR_016828 respectively, target the transcription factor *SMAD2* at the 3’ UTR (**Figure 3B**).

Interestingly, chemokine signaling has been shown to contribute to fibrotic remodeling *via* fibrogenic/alternative macrophages and through pro-fibrotic actions such as recruitment of activated neutrophils and fibrogenic monocytes. The link between chemokine signaling during early *T. cruzi* infection in heart cells remains to be investigated. Some of the DE piRNAs were computationally predicted to target *CX3CL1* and *CXCR2*, respectively. Two known piRNAs, piR_016742 and piR_017716 were predicted to target an exon and the 3’ UTR of *CX3CL1*, respectively (**Figure 3C**). Similarly, piR_017716 was shown to target the 3’ UTR of *CXCR2* (**Figure 3D**). We previously showed in Udoko et al. (Udoko et al., 2016) that *T. cruzi* dysregulates the gene expression profile of cardiac myocytes during the early phase of infection. In that report we showed that the parasite significantly dysregulates the expression of several profibrotic transcription factors including *EGR1*. Here, we show that a putative piRNA, npiR_167, which is DE during the early phase of parasite challenge is computationally predicted to target an exon region of *EGR1* (**Figure 3E**).

### 3.4 Validation of differentially expressed piRNAs and their targets

**Figure 4:**
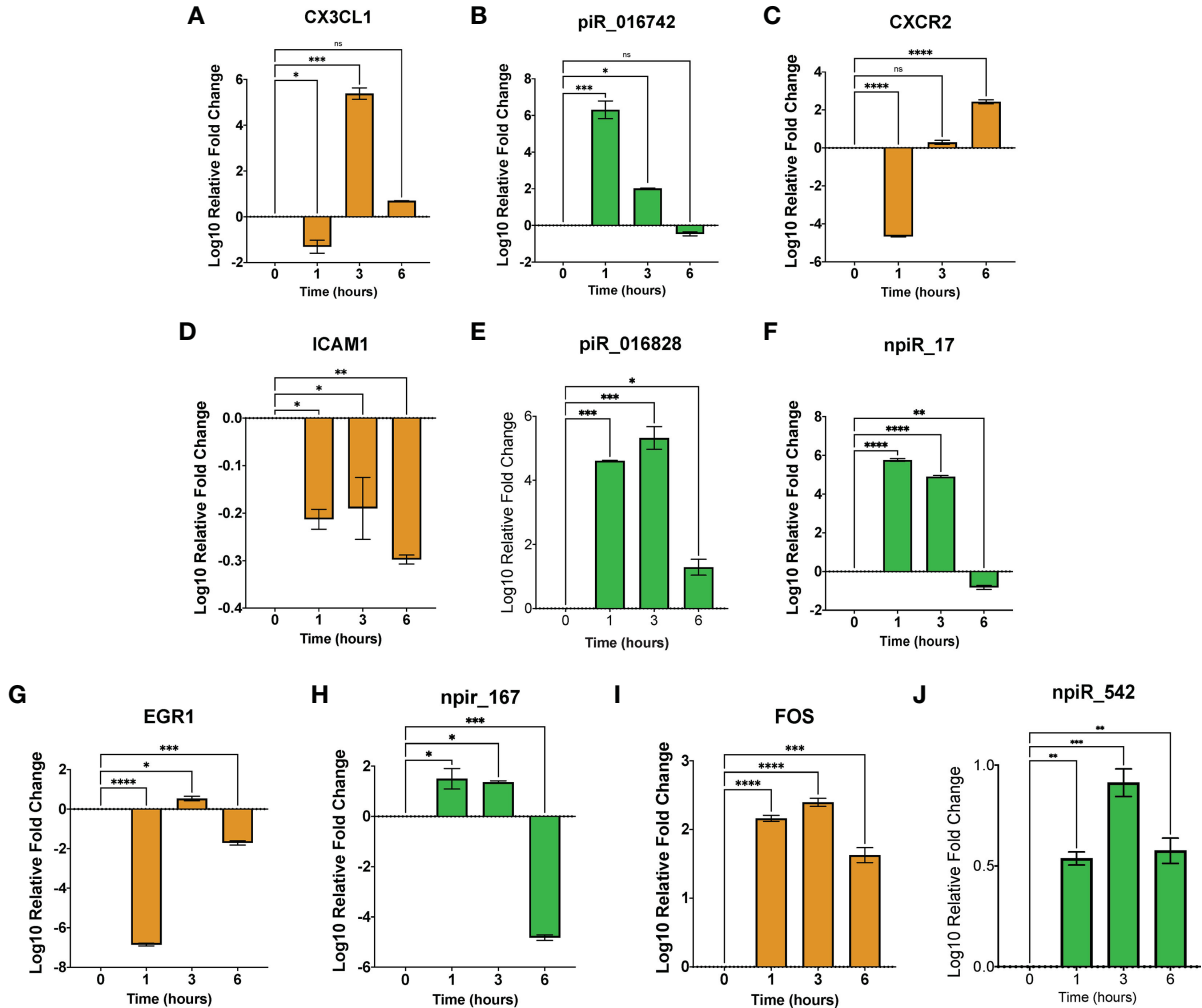
Early *T. cruzi* infection dysregulates expression of piRNAs and their targets. **(A–J)**. Total RNA and small RNA were purified from PHCF challenge with invasive *T. cruzi* trypomastigotes. Target transcript expression was evaluated using RT-qPCR, while piRNA expression was validated using stem-loop PCR for cDNA synthesis and RT-qPCR for relative expression. Bar graphs show log_10_ fold change ± SEM (n=3). Orange bar graphs denote relative mRNA target transcript expression while green bar graphs represent relative piRNA expression. Student’s t-test and visualization performed using GraphPad Prism. ns = not significant, *p < .05, **p < .01, ***p < 0.001, ****p < 0.0001.

We validated the expression of the selected dysregulated piRNAs and their target genes (*ICAM1*, *SMAD2*, *CX3CL1*, *CXCR2* and *EGR1*), which are implicated in *T. cruzi* infection and pathogenesis by qPCR. Total and small RNAs purified from *T. cruzi* challenged PHCF were used in our validation assays. Stem-loop PCR primers were designed to reverse transcribe each individual specific mature piRNA and U6 snRNA (housekeeping). The generated cDNA was quantified in the second step of the PCR by qPCR; a two-step RT-qPCR. Gene targets were evaluated using RT-qPCR as well. Our qPCR data show that during parasite challenge, the normalized fold change of *CX3CL1* transcript showed a significant decrease at 1 h followed by a relative increase in expression at 3 h and 6 h, respectively (**Figure 4A**). The targeting piR_016742 was shown to be upregulated at 1 and 3 h relative to 0, with a non-significant decrease at 6 h. This dynamic normalized expression negatively correlates to normalized expression levels of *CX3CL1* transcript expression (**Figure 4B**). *CXCR2* showed a normalized decreased expression at 1 h followed by a normalized increase that was maximum at the 6 h time point (**Figure 4C**). We previously reported that piR_017716, computationally predicted to target both *CXCR2* and *CX3CL1* was significantly decreased at 6 h (Rayford et al., 2022). More studies are needed to understand piRNA mediated regulation of *CXCR2*. The transcript levels of *ICAM1* were significantly decreased at all time points. *ICAM1* is uniquely targeted by 2 piRNAs, piR_016828 and npiR_17. Both piRNAs are significantly upregulated at 1 and 3 h relative to 0 h. Putative npiR_17 decreases at 6 h while piR_016828 remains significantly increased (**Figures 4E, F**). We previously showed that *EGR1* expression during *T. cruzi* infection of cardiac myocytes increases to activate profibrotic molecules. Interestingly, *EGR1* transcript expression showed a significant decrease at 1 and 6 h with a significant increase at 3 h relative to 0 h (**Figure 4G**). The novel piRNA, npiR_167, predicted to target an exon of *EGR1*, showed a significant increase at 1 and 3 h, followed by a significant decrease at 6 h (**Figure 4H**). Accumulating evidence has shown that AP-1 protein FOS can play a role in early *T. cruzi* pathogenesis by activating profibrotic genes (Moreira et al., 2017; Choudhuri and Garg, 2020). We predicted in Rayford et al. (Rayford et al., 2020) that several piRNAs can target *FOS* transcript in *T. cruzi* challenged PHCM. Here, we evaluated the *FOS* transcript expression and show that it remained significantly increased from 1 to 6 h compared to control (**Figure 4I**), while the npiR_542 computationally predicted to target *FOS* was also significantly increased at all time points (**Figure 4J**).

### 3.5 Biological interaction networks of differentially expressed piRNAs and their targets during early *T. cruzi* infection

**Figure 5:**
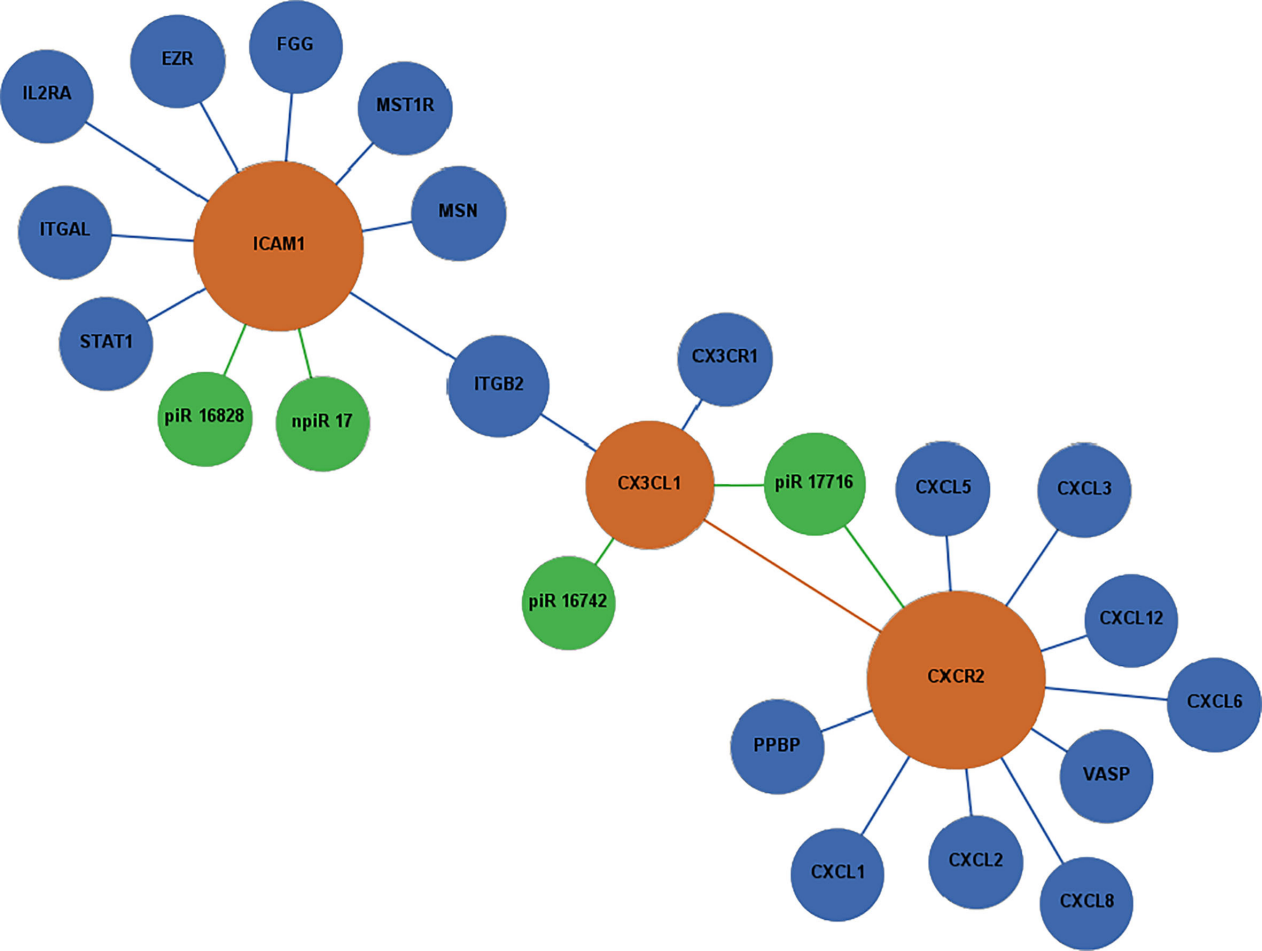
Network of differentially expressed piRNAs targeting adhesion and chemokine signaling molecules operating during *T. cruzi* challenge of PHCF. Biological interaction networks were created using predicted piRNA targets (ICAM1, CX3CL1, and CXCR2) as primary seed nodes to query pathway and interaction data sources out to one degree of biological interaction. Primary seed notes are denoted as orange circles. Expansion notes (blue) with a predicted binding target of DE piRNAs (green).

**Figure 6:**
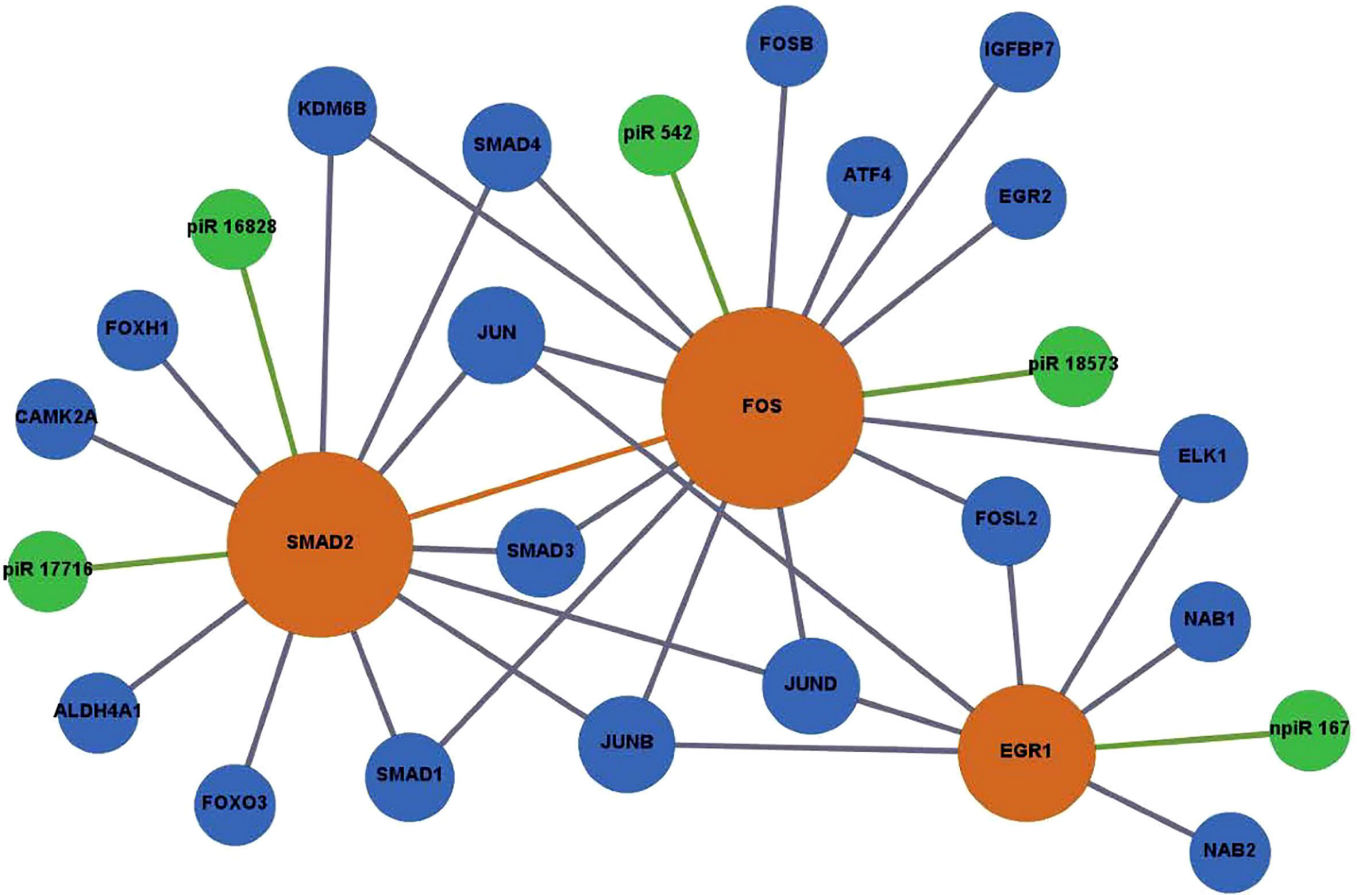
Network of DE piRNAs targeting profibrotic transcription factors during PHCF challenged with *T. cruzi*. Biological interaction networks were created using predicted piRNA targets (SMAD2, FOS, and EGR1) as primary seed nodes to query pathway and interaction data sources out to one degree of biological interaction. Primary seed notes are displayed as orange circles. Expansion notes (blue) with a predicted binding target of DE piRNAs (green).

To identify the molecular mechanisms that could be activated in PHCF and regulated by DE piRNAs induced during the early phase of cellular infection by *T. cruzi*, we used the GeneMANIA algorithm to construct two biological interaction networks based on adhesion molecule and chemokine expression (ICAM1, CX3CL1, CXCR2) and the AP-1 transcription factor family (FOS, SMAD2, EGR1). These two sets of query molecules were expanded to one degree of molecular protein–protein interactions and then connected with validated known and putative piRNAs. The resulting networks were visualized with Gephi. We show that through predicted targeting of CXCR2, piR_017716 may influence the activity of the durotactic mediator vasodilator-stimulated phosphoprotein (VASP), some molecules involved in fibrotic responses including CXCL2, CXCL3, and CXCL8. ICAM1 targeted by piR_016828 and npiR_17, and CX3CL1 targeted by piR_016742 and piR_017716 interacted with various molecules including fibrinogens, integrins, and other regulators of the inflammatory response such as STAT1 and IL2RA (**Figure 5**). SMAD2 was predicted to be targeted by piR_016828 and piR_017716 and FOS by piR_542 and piR_018573. Putative piRNA npiR_167 is the sole piRNA predicted to target EGR1, which is directly connected to several AP-1 family proteins. Molecules interacting with FOS, SMAD2, and EGR1 are involved in TGF-β signaling through FOXH, SMAD3, and SMAD4. Epidermal growth factor (EGF) signaling may also be impacted through CAMK2A, ELK1, FOSB, JUN and JUND (**Figure 6**).

## 4 Discussion

*T. cruzi*, the etiological agent of Chagas Disease, causes severe morbidity, mortality, and economic burden globally. Recent studies emphasize increased surveillance and screening of *T. cruzi* infection in the US, following increased detections of autochthonous infections (Dorn et al., 2007; Rassi et al., 2010; Beatty and Klotz, 2020; Lynn et al., 2020). Chronic infections have been associated with the development of cardiomyopathies including but not limited to myocardial hypertrophy, cardiac fibrosis, and cardiomegaly, among others. Though current research aims to understand gene expression regulation in cardiac tissue, few studies have been focused on the contribution of cardiac fibroblasts to infection and pathogenesis. Upon injury, cardiac fibroblasts differentiate into myofibroblasts to produce excessive amounts of extracellular and other profibrogenic molecules. This unregulated response can lead to the onset of *T. cruzi*-induced cardiomyopathies. However, the molecular mechanisms by which *T. cruzi* alters the gene expression profile in cardiac fibroblasts remains to be fully understood. Ongoing research seeks to understand alterations in gene regulation during parasite infection.

Small noncoding RNAs such as miRNAs and piRNAs are potent modulators of gene expression and regulation, and thus have become important in understanding dysregulation of host gene expression during disease pathogenesis. In recent years, we and others have begun to investigate the role of small noncoding RNAs in *T. cruzi* pathogenesis. Ferreira et al. linked altered miRNA expression and their targets to various *T. cruzi* pathologies such as hypertrophy and fibrosis (Ferreira et al., 2017). The traditional sncRNA regulation paradigm proposes miRNAs directly regulate transcript and protein expression *via* binding to targets when complexed with RISC (Ferreira et al., 2017). In the new world of gene regulation, piRNAs have been suggested to regulate gene expression, transposon silencing, *de novo* methylation, chromatin remodeling, and translational activation when complexed with PIWI proteins (Ku and Lin, 2014; Rayford et al., 2021). Recently, we showed in infectious disease research that *T. cruzi* dysregulates the expression profile of host piRNAs computationally predicted to target profibrotic molecules including *TGFB1*, *FOS*, and *NFATC2* in primary human cardiac myocytes (Rayford et al., 2020). Here, we hypothesize that *T. cruzi* dysregulates the expression of piRNAs in PHCF during the acute phase of infection. The dysregulated piRNAs can regulate the gene expression profile of profibrotic and inflammatory molecules that we and others reported (Suzin et al., 1987; Waghabi et al., 2005; Kayama et al., 2009; Udoko et al., 2016).

We challenged PHCF with the parasite at different time points and evaluated the piRNome employing an approach that results in lower error accumulation and sequencing bias, thereby improving the quality of our data as we recently described (Rayford et al., 2020). PHCF constitute a good model that closely mimics the natural infection. We anticipate that the data obtained from *in vitro* assays using PHCF will mimic changes in host piRNome profile induced by the parasite in a natural infection. The findings will guide studies geared towards understanding parasite mediated pathology in complex microenvironment in subsequent studies. *In silico* analysis showed that replicates clustered independently from each other and controls, indicating substantial alterations in the piRNA expression profile (**Figures 1A, B**, **2A, D**). Though piRNAs are derived from a plethora of origins, we found that the significantly DE piRNAs could be classified into various TE families such as LINEs, LTRs, and class 2 DNA transposons (**Figures 1C-E**). The pattern of piRNAs mapped to different transposable element subfamilies agrees with our observations in PHCM (Rayford et al., 2020). Over 85% of the DE piRNAs were derived from LTRs and LINEs, which suggests that these TEs may serve as important precursor sources of piRNA biogenesis in cardiac cells during *T. cruzi* infection. The high percentage of piRNAs originating from LTRs and LINEs suggests that they play a key role in the regulation of gene expression during the early phase of *T. cruzi* infection in PHCF compared to PHCM where the large majority of DE piRNAs were from LTRs (Rayford et al., 2020).

Our data show that the parasite induced significant DE of known and novel piRNAs at different time points (**Figures 2A-G**). The majority of the DE piRNAs that we report here are novel. NCBI BLAST search analysis of the putative piRNA sequences against the *T. cruzi* sequence database showed no significant alignments, suggesting that the piRNAs are host-derived. These novel putative piRNAs exhibit characteristics of canonical piRNAs that are yet to be annotated in the human genome (**Supplementary Tables 1**, **2**).

We showed in our previous publication that miRANDA and RNA22 algorithms could be adapted for predicting piRNA targets (Rayford et al., 2020). We used miRANDA to computationally predict the piRNA gene targets. We created a list of genes most pertinent to *T. cruzi* infection and fibrosis by using Geneshot to search aggregate publications and gene expression repositories (Lachmann et al., 2019). From our list of piRNA target genes we identified *ICAM1*, *SMAD2*, *CX3CL1*, *CXCR2*, and *EGR1* for further evaluation and validation in this report (**Figures 3**, **4**). The selected genes have been suggested to play roles in *T. cruzi* infection and pathology (Blanton et al., 1991; Laucella et al., 1996; Huang et al., 1999; Michailowsky et al., 2004; Shen et al., 2019). We validated the relative expression levels of the selected piRNAs of interest and their gene targets (**Figure 4**). Several studies have linked ICAM1 as a driver of cardiac remodeling in human, rat, and mouse models (Luc et al., 2003; Davani et al., 2006; Fotis et al., 2012; Lino et al., 2019). Studies examining chronic *T. cruzi* infection suggest upregulation of various vascular adhesion molecules such as VCAM1 and ICAM1 (Laucella et al., 1996; Huang et al., 1999; Michailowsky et al., 2004). We found that during the acute phase of infection, *ICAM1* transcript levels were significantly decreased at all time points following *T. cruzi* challenge. Conversely, piR_016828 expression was significantly increased at all corresponding time points. Similarly, npiR_17 significantly increased at 1 and 3 hours but significantly decreased at 6 hours. Our data suggest that these two piRNAs predicted to target *ICAM1* may in part contribute to the downregulation of *ICAM1* transcript observed during early infection. It will be interesting to evaluate the levels of the *ICAM* and the target piRNAs during the chronic phase of *T. cruzi* infection as a potential biomarker for cardiac remodeling. Few studies have examined the role of *CX3CL1* in *T. cruzi* pathogenesis, however Zhang et al. observed that the expression of *CX3CL1* is increased in liver fibrosis caused by *Schistosoma* infection (Zhang et al., 2020). Here, we observed a significant decrease in *CX3CL1* expression at 1 hour following parasite challenge with a significant increase at 3 and 6 hours. The expression of piR_016742 was significantly increased at 1 and 2 hours and significantly decreased at 6 hours. This inverse expression relationship between the piRNA and transcript suggests that piR_016742 may negatively regulate *CX3CL1* expression. CXCR2 is a G-protein-coupled receptor that binds to interleukin 8 (IL8) with high affinity. This receptor also binds to CXCL1 and CXCL8 (Li and Frangogiannis, 2021). Previous studies show that CXCR2 activation can be cardioprotective or pathogenic, depending on the cardiovascular disease (Tarzami et al., 2003; Liehn et al., 2013). Schmitz et al. found that CXCR2 antagonism prevented *T. cruzi* trypomastigote-induced plasma leakage, suppressing the inflammatory response to the parasite (Blanton et al., 1991). During parasite challenge in PHCF, *CXCR2* expression was significantly downregulated at 1 h but recovered at 6 h post infection. Interestingly piR_017716 showed nonsignificant increases at 1 and 3 h but significantly decreased at 6 h. This correlation implicates an inverse relationship and potential negative regulation of *CXCR2* by piR_017716. EGR1 is a transcription regulator induced by growth factors, cytokines, and stress signals such as radiation, injury, or mechanical stress to transduce signals that activate downstream signaling cascades. Various fibrogenic diseases are associated with elevated levels of EGR1 and TGF-β. We and others show that *T. cruzi* infection upregulates the expression of EGR1 in various cell types (Mott et al., 2011; Udoko et al., 2016; Shen et al., 2019). In *T. cruzi* challenged PHCF, *EGR1* expression was significantly decreased at the 1 h time point, followed by a significant increase at 3 h, which returned to a significant decrease at 6 h. The expression of npiR_167 does not appear to strongly correlate with *EGR1* transcript levels. In another study, we showed that *T. cruzi* upregulated the transcript and protein levels of EGR1 in PHCM during the early phase of infection (Udoko et al., 2016), an observation which is different in parasite challenged PHCF. *EGR1* upregulation in PHCM potentially leads to increased dysregulation of extracellular signals that can alter fibroblast response in cardiac tissue. Within the same period, the parasite induced an increase in the expression of FOS, an AP-1 transcription factor at all time points as we showed before with PHCM (Udoko et al., 2016). The novel piRNA shown to target *FOS* transcript, npiR_542 was also increased at all time points suggesting that this piRNA may function to stabilize the transcript for increased FOS protein expression. Since increased FOS expression has been associated with fibrogenic responses (Eferl et al., 2008; Nakagawa et al., 2016), its regulatory piRNAs can be developed to serve as fibrogenic biomarkers and/or therapeutic targets. Ongoing studies in the laboratory are geared towards determining the exact molecular mechanisms by which the DE piRNAs regulate gene expression to facilitate *T. cruzi* pathogenesis.

Selected DE piRNAs and their predicted targets ICAM1, SMAD2, CX3CL1, CXCR2, and EGR1 were used to generate networks that linked the piRNAs to their target genes and expanded them to one degree of biological interaction. Our *in silico* analysis showed that some of the differentially expressed piRNAs (known and novel) can target genes that we and others reported to be dysregulated during the early phase of *T. cruzi* infection. Two networks were generated based on chemokine signaling (**Figure 5**) and profibrotic transcription factors (**Figure 6**). In the chemokine network, CXCR2 had the most protein-protein interactions and was connected to ICAM1 through CX3CL1 and integrin beta 2 (ITGB2). We observed that the piRNAs dysregulated by the parasite may have an influence on other chemokines and proteins previously implicated in *T. cruzi* infection and Chagas disease. VASP is a signaling pathway that regulates integrin-extracellular matrix interactions directly modulating the actin ultrastructure. Changes in VASP activity have been linked to cardiomyopathies (Pula and Krause, 2008). *T. cruzi* infection triggers substantial production of nitric oxide (NO), which has been shown to have protective and toxic effects on the host’s immune system (Bergeron and Olivier, 2006). Integrin alpha-L chain (ITGAL) and ITGB2 form lymphocyte function-associated antigen 1 (LFA-1) and binds with ICAMs to facilitate intercellular adhesion of leukocytes. Integrins mediate lymphocyte migration into an infected tissue, and these cells are essential for regulating parasitemia, clearance, and inflammation (Acevedo et al., 2018). Mice treated with anti-LFA-1 displayed increased blood and tissue parasitemia, and quickly succumbed to infection (Ferreira et al., 2017). *T. cruzi* reduces interleukin 2 receptor alpha (IL2RA) expressed on the surface of immune cells, suppressing the immune response (Majumder and Kierszenbaum, 1996). Levels of pro-platelet basic protein (PPBP) and other peripheral blood mononuclear cells correlate with disease state in Chagas disease (Garg et al., 2016; Zago et al., 2018).

The DE piRNAs were also predicted to target sequences on genes that were within one degree of interaction with the AP-1 transcription factors evaluated in this study (**Figures 5**, **6**). In the profibrotic transcription factor network, FOS had the most interactions. We and others have described alterations in the AP-1 gene and protein expression profiles during parasite infection. In addition to the primary AP-1 family proteins in the seed nodes, other proteins linked to them by one degree of biological interaction could be implicated in piRNA regulation; NAB1 and NAB2 are transcriptional cofactors connected to EGR1. Transgenic mice exhibiting cardiac-specific overexpression of NAB1, revealed that NAB1 inhibits cardiac growth in response to pathological stimuli *in vivo*. NAB1 overexpression inhibited pressure overload–induced hypertrophy (Buitrago et al., 2005). NAB2 directly binds to EGR1 and regulates EGR1 gene transcription. Additionally, NAB2 expression is induced by TGF-β, which is suggested to play an important role in *T. cruzi* infection and pathogenesis (Waghabi et al., 2007; Bhattacharyya et al., 2009; Waghabi et al., 2009). In dermal fibroblasts, it was found that expression of NAB2 correlated with persistent fibrotic responses (Bhattacharyya et al., 2009; Bhattacharyya et al., 2011). CAMK2A, connected to SMAD2, is a major mediator of calcium signaling in the heart and it was suggested that *T. cruzi* uses calcium signaling to facilitate host cell invasion (Benaim et al., 2020). Inhibition of CAMK2A was shown to reduce occurrence of cardiac arrhythmias for *in vivo* and *ex vivo* Chagas disease models (Santos-Miranda et al., 2021). Elevated levels of insulin-like growth factor binding protein 7 (IGFBP7) have been observed in patients with heart failure and correlate with worse disease outcomes (Januzzi et al., 2018). In contrast, a study on dermal fibroblasts exposed to *T. cruzi* soluble factor resulted in reduced levels of IGFBP7 (Mott et al., 2011). SMAD2, a member of the SMAD protein family, is a signal transducer and transcriptional modulator that mediates multiple signaling pathways. The phosphorylated version of SMAD2 is downstream of TGFβ signaling, thus it regulates multiple cellular processes. Accumulating evidence establishes that SMAD/TGFβ signaling is required for *T. cruzi* infection and pathogenesis (Udoko et al., 2016; Ferreira et al., 2019; Silva et al., 2019). Accordingly, these selected DE piRNAs highlighted in our manuscript and their gene targets can be suggested to play significant roles in cardiopathogenesis as summarized in **Table 1**. These piRNAs can be developed as potential therapeutic targets against the onset of *T. cruzi* induced cardiomyopathies.

**Table 1:** Dysregulated piRNAs and their predicted targets linked to various cardiovascular pathologies.

piRNA	Gene target	Disease/pathology	Role in disease/pathology	Reference
npiR_17 and piR_016828	*ICAM1*	Incident coronary heart disease	Not identified	(Luc et al., 2003; Luo et al., 2014; Yin et al., 2019)
piR_017 716 and piR_016828	*SMAD2*	Cardiac fibrosis	Activation of profibrotic molecules	(Chen et al., 2019)
		Rheumatic heart disease	Activates endothelial to mesenchymal transition (EndMT)	(Xian et al., 2021)
piR_016742 and piR_017716	*CX3CL1*	Coronary artery disease	Not identified	(Damas et al., 2005)
		Heart failure		(Husberg et al., 2008)
piR_017716	*CXCR2*	Heart failure	Hypertrophic remodeling	(Wang et al., 2018; Zhang et al., 2019)
npiR_167	*EGR1*	Cardiac hypertrophy	Tissue thickening	(Khachigian, 2006)
		Myocardial infarction	Myocardial inflammation and apoptosis	(Rayner et al., 2013)

Taken together, our data show that during the early phase of *T. cruzi* challenge of PHCF, the parasite induces DE of piRNAs (both known and novel) that can target and regulate the expression of important profibrotic and inflammatory molecules. This finding is significant considering the extensive regulatory roles of piRNAs. This report showing that a pathogen can dysregulate host piRNA expression, leading to enhanced understanding of parasite-induced gene regulation, which is essential for the identification of biomarkers and the development of molecular intervention strategies during the early phase of *T. cruzi* infection.

## Data availability statement

The datasets presented in this study can be found in online repositories. The names of the repository/repositories and accession number(s) can be found in the article/**Supplementary Material**.

## Author contributions

Conceptualization, PN,; methodology, KR, AC, SP, PN; software, AC, SP; validation, KR, AC, SM, SP, PN; formal analysis, KR, AC, AA, SM, SP, ML, PN; investigation, KR, AC, SM, SP, PN; resources, SP, PN; data curation, KR, AC, SP; PN writing—original draft preparation, KR, AC, AA, SM, PN; writing—review and editing, KR, AC, AA, SM, PN; visualization, KR, AC, SP, PN; supervision, SP, PN; project administration, PN; funding acquisition, KR, ML, SP, PN. All authors contributed to the article and approved the submitted version.

## References

[B1] AboEllail M. A. M. Hata T. (2017). Fetal face as important indicator of fetal brain function. J. Perinat Med. 45 (6), 729–736. doi: 10.1515/jpm-2016-0377 28130960

[B2] Acevedo G. R. Girard M. C. Gomez K. A. (2018). The unsolved jigsaw puzzle of the immune response in chagas disease. Front. Immunol. 9, 1929. doi: 10.3389/fimmu.2018.01929 30197647 PMC6117404

[B3] Beatty N. L. Klotz S. A. (2020). Autochthonous chagas disease in the united states: How are people getting infected? Am. J. Trop. Med. Hyg 103 (3), 967–969. doi: 10.4269/ajtmh.19-0733 32602437 PMC7470559

[B4] Benaim G. Paniz-Mondolfi A. E. Sordillo E. M. Martinez-Sotillo N. (2020). Disruption of intracellular calcium homeostasis as a therapeutic target against trypanosoma cruzi. Front. Cell Infect. Microbiol. 10, 46. doi: 10.3389/fcimb.2020.00046 32133302 PMC7040492

[B5] Bergeron M. Olivier M. (2006). Trypanosoma cruzi-mediated IFN-gamma-inducible nitric oxide output in macrophages is regulated by iNOS mRNA stability. J. Immunol. 177 (9), 6271–6280. doi: 10.4049/jimmunol.177.9.6271 17056557

[B6] Bern C. Messenger L. A. Whitman J. D. Maguire J. H. (2019). Chagas disease in the united states: A public health approach. Clin. Microbiol. Rev. 33 (1). doi: 10.1128/CMR.00023-19 PMC6927308 31776135

[B7] Bhattacharyya S. Wei J. Melichian D. S. Milbrandt J. Takehara K. Varga J. (2009). The transcriptional cofactor nab2 is induced by tgf-beta and suppresses fibroblast activation: Physiological roles and impaired expression in scleroderma. PloS One 4 (10), e7620. doi: 10.1371/journal.pone.0007620 19888474 PMC2768752

[B8] Bhattacharyya S. Wu M. Fang F. Tourtellotte W. Feghali-Bostwick C. Varga J. (2011). Early growth response transcription factors: key mediators of fibrosis and novel targets for anti-fibrotic therapy. Matrix Biol. 30 (4), 235–242. doi: 10.1016/j.matbio.2011.03.005 21511034 PMC3135176

[B9] Blanton R. E. Okelo G. B. Kijobe J. King C. H. (1991). Antibody responses to *in vitro* translation products following albendazole therapy for echinococcus granulosus. Antimicrob. Agents Chemother. 35 (8), 1674–1676. doi: 10.1128/AAC.35.8.1674 1929340 PMC245241

[B10] Bonney K. M. Engman D. M. (2008). Chagas heart disease pathogenesis: One mechanism or many? Curr. Mol. Med. 8 (6), 510–518. doi: 10.2174/156652408785748004 18781958 PMC2859714

[B11] Bonney K. M. Luthringer D. J. Kim S. A. Garg N. J. Engman D. M. (2019). Pathology and pathogenesis of chagas heart disease. Annu. Rev. Pathol. 14, 421–447. doi: 10.1146/annurev-pathol-020117-043711 30355152 PMC7373119

[B12] Buitrago M. Lorenz K. Maass A. H. Oberdorf-Maass S. Keller U. Schmitteckert E. M. (2005). The transcriptional repressor Nab1 is a specific regulator of pathological cardiac hypertrophy. Nat. Med. 11 (8), 837–844. doi: 10.1038/nm1272 16025126

[B13] Chen H. Moreno-Moral A. Pesce F. Devapragash N. Mancini M. Heng E. L. (2019). WWP2 regulates pathological cardiac fibrosis by modulating SMAD2 signaling. Nat. Commun. 10 (1), 3616. doi: 10.1038/s41467-019-11551-9 31399586 PMC6689010

[B14] Choudhuri S. Garg N. J. (2020). Trypanosoma cruzi induces the PARP1/AP-1 pathway for upregulation of metalloproteinases and transforming growth factor beta in macrophages: Role in cardiac fibroblast differentiation and fibrosis in chagas disease. mBio 11 (6). doi: 10.1128/mBio.01853-20 PMC7667027 33172999

[B15] Coura J. R. Vinas P. A. (2010). Chagas disease: A new worldwide challenge. Nature 465 (7301), S6–S7. doi: 10.1038/nature09221 20571554

[B16] Cruz J. S. Santos-Miranda A. Sales-Junior P. A. Monti-Rocha R. Campos P. P. Machado F. S. (2016). Altered cardiomyocyte function and trypanosoma cruzi persistence in chagas disease. Am. J. Trop. Med. Hyg 94 (5), 1028–1033. doi: 10.4269/ajtmh.15-0255 26976879 PMC4856598

[B17] Cunningham D. A. Lin J. W. Brugat T. Jarra W. Tumwine I. Kushinga G. (2017). ICAM-1 is a key receptor mediating cytoadherence and pathology in the plasmodium chabaudi malaria model. Malar J. 16 (1), 185. doi: 10.1186/s12936-017-1834-8 28468674 PMC5415785

[B18] da Costa A. W. F. do Carmo Neto J. R. Braga Y. L. L. Silva B. A. Lamounier A. B. Silva B. O. (2019). Cardiac chagas disease: MMPs, TIMPs, galectins, and TGF-beta as tissue remodelling players. Dis. Markers 2019 p, 3632906. doi: 10.1155/2019/3632906 PMC6899287 31885735

[B19] Damas J. K. Boullier A. Waehre T. Smith C. Sandberg W. J. Green S. (2005). Expression of fractalkine (CX3CL1) and its receptor, CX3CR1, is elevated in coronary artery disease and is reduced during statin therapy. Arterioscler. Thromb. Vasc. Biol. 25 (12), 2567–2572. doi: 10.1161/01.ATV.0000190672.36490.7b 16224053

[B20] Dana H. Chalbatani G. M. Mahmoodzadeh H. Karimloo R. Rezaiean O. Moradzadeh A. (2017). Molecular mechanisms and biological functions of siRNA. Int. J. BioMed. Sci. 13 (2), 48–57. 28824341 PMC5542916

[B21] Das A. Samidurai A. Salloum F. N. (2018). Deciphering non-coding RNAs in cardiovascular health and disease. Front. Cardiovasc. Med. 5, 73. doi: 10.3389/fcvm.2018.00073 30013975 PMC6036139

[B22] Davani E. Y. Boyd J. H. Dorscheid D. R. Wang Y. Meredith A. Chau E. (2006). Cardiac ICAM-1 mediates leukocyte-dependent decreased ventricular contractility in endotoxemic mice. Cardiovasc. Res. 72 (1), 134–142. doi: 10.1016/j.cardiores.2006.06.029 16934241

[B23] Dorn P. L. Perniciaro L. Yabsley M. J. Roellig D. M. Balsamo G. Diaz J. (2007). Autochthonous transmission of trypanosoma cruzi, Louisiana. Emerg. Infect. Dis. 13 (4), 605–607. doi: 10.3201/eid1304.061002 17553277 PMC2725963

[B24] Drmanac R. Sparks A. B. Callow M. J. Halpern A. L. Burns N. L. Kermani B. G. (2010). Human genome sequencing using unchained base reads on self-assembling DNA nanoarrays. Science 327 (5961), 78–81. doi: 10.1126/science.1181498 19892942

[B25] Eferl R. Hasselblatt P. Rath M. Popper H. Zenz R. Komnenovic V. (2008). Development of pulmonary fibrosis through a pathway involving the transcription factor fra-2/AP-1. Proc. Natl. Acad. Sci. U.S.A. 105 (30), 10525–10530. doi: 10.1073/pnas.0801414105 18641127 PMC2492511

[B26] Efron B. Tibshirani R. Storey J. D. Tusher V. (2001). Empirical bayes analysis of a microarray experiment. J. Am. Stat. Assoc. 96 (456), 1151–1160. doi: 10.1198/016214501753382129

[B27] Enright A. J. John B. Gaul U. Tuschl T. Sander C. Marks D. S. (2003). MicroRNA targets in drosophila. Genome Biol. 5 (1), R1. doi: 10.1186/gb-2003-5-1-r1 14709173 PMC395733

[B28] Fehlmann T. Reinheimer S. Geng C. Su X. Drmanac S. Alexeev A. (2016). cPAS-based sequencing on the BGISEQ-500 to explore small non-coding RNAs. Clin. Epigenet. 8, 123. doi: 10.1186/s13148-016-0287-1 PMC5117531 27895807

[B29] Ferrao P. M. d'Avila-Levy C. M. Araujo-Jorge T. C. Degrave W. M. Goncalves Ada S. Garzoni L. R. (2015). Cruzipain activates latent TGF-beta from host cells during t. cruzi invasion. PLoS One 10 (5), e0124832. doi: 10.1371/journal.pone.0124832 25938232 PMC4418758

[B30] Ferreira C. P. Cariste L. M. Santos Virgilio F. D. Moraschi B. F. Monteiro C. B. Vieira Machado A. M. (2017). LFA-1 mediates cytotoxicity and tissue migration of specific CD8(+) T cells after heterologous prime-boost vaccination against trypanosoma cruzi infection. Front. Immunol. 8, 1291. doi: 10.3389/fimmu.2017.01291 29081775 PMC5645645

[B31] Ferreira L. R. P. Ferreira F. M. Laugier L. Cabantous S. Navarro I. C. da Silva Candido D. (2017). Integration of miRNA and gene expression profiles suggest a role for miRNAs in the pathobiological processes of acute trypanosoma cruzi infection. Sci. Rep. 7 (1), 17990. doi: 10.1038/s41598-017-18080-9 29269773 PMC5740174

[B32] Ferreira R. R. (2019). TGF-beta inhibitor therapy decreases fibrosis and stimulates cardiac improvement in a pre-clinical study of chronic chagas' heart disease. PLoS Negl. Trop. Dis. 13 (7), e0007602. doi: 10.1371/journal.pntd.0007602 31365537 PMC6690554

[B33] Fotis L. Agrogiannis G. Vlachos I. S. Pantopoulou A. Margoni A. Kostaki M. (2012). Intercellular adhesion molecule (ICAM)-1 and vascular cell adhesion molecule (VCAM)-1 at the early stages of atherosclerosis in a rat model. In Vivo 26 (2), 243–250. 22351665

[B34] Fu Y. Wu P. H. Beane T. Zamore P. D. Weng Z. (2018). Elimination of PCR duplicates in RNA-seq and small RNA-seq using unique molecular identifiers. BMC Genomics 19 (1), 531. doi: 10.1186/s12864-018-4933-1 30001700 PMC6044086

[B35] Garg N. J. Soman K. V. Zago M. P. Koo S. J. Spratt H. Stafford S. (2016). Changes in proteome profile of peripheral blood mononuclear cells in chronic chagas disease. PloS Negl. Trop. Dis. 10 (2), e0004490. doi: 10.1371/journal.pntd.0004490 26919708 PMC4769231

[B36] Gascon J. Bern C. Pinazo M. J. (2010). Chagas disease in Spain, the united states and other non-endemic countries. Acta Trop. 115 (1-2), 22–27. doi: 10.1016/j.actatropica.2009.07.019 19646412

[B37] Halajzadeh J. Dana P. M. Asemi Z. Mansournia M. A. Yousefi B. (2020). An insight into the roles of piRNAs and PIWI proteins in the diagnosis and pathogenesis of oral, esophageal, and gastric cancer. Pathol. Res. Pract. 216 (10), 153112. doi: 10.1016/j.prp.2020.153112 32853949

[B38] Herum K. M. Choppe J. Kumar A. Engler A. J. McCulloch A. D. (2017). Mechanical regulation of cardiac fibroblast profibrotic phenotypes. Mol. Biol. Cell 28 (14), 1871–1882. doi: 10.1091/mbc.e17-01-0014 28468977 PMC5541838

[B39] Hortells L. Johansen A. K. Z. Yutzey K. E. (2019). Cardiac fibroblasts and the extracellular matrix in regenerative and nonregenerative hearts. J. Cardiovasc. Dev. Dis. 6 (3). doi: 10.3390/jcdd6030029 PMC6787677 31434209

[B40] Hotez P. J. Dumonteil E. Betancourt Cravioto M. Bottazzi M. E. Tapia-Conyer R. Meymandi S. (2013). An unfolding tragedy of chagas disease in north America. PloS Negl. Trop. Dis. 7 (10), e2300. doi: 10.1371/journal.pntd.0002300 24205411 PMC3814410

[B41] Huang H. Calderon T. M. Berman J. W. Braunstein V. L. Weiss L. M. Wittner M. (1999). Infection of endothelial cells with trypanosoma cruzi activates NF-kappaB and induces vascular adhesion molecule expression. Infect. Immun. 67 (10), 5434–5440. doi: 10.1128/IAI.67.10.5434-5440.1999 10496926 PMC96901

[B42] Husberg C. Nygard S. Finsen A. V. Damas J. K. Frigessi A. Oie E. (2008). Cytokine expression profiling of the myocardium reveals a role for CX3CL1 (fractalkine) in heart failure. J. Mol. Cell Cardiol. 45 (2), 261–269. doi: 10.1016/j.yjmcc.2008.05.009 18585734

[B43] Ivey M. J. Tallquist M. D. (2016). Defining the cardiac fibroblast. Circ. J. 80 (11), 2269–2276. doi: 10.1253/circj.CJ-16-1003 27746422 PMC5588900

[B44] Januzzi J. L. Jr. Packer M. Claggett B. Liu J. Shah A. M. Zile M. R. (2018). IGFBP7 (Insulin-like growth factor-binding protein-7) and neprilysin inhibition in patients with heart failure. Circ. Heart Fail 11 (10), e005133. doi: 10.1161/CIRCHEARTFAILURE.118.005133 30354399 PMC12611560

[B45] John B. Enright A. J. Aravin A. Tuschl T. Sander C. Marks D. S. (2004). Human MicroRNA targets. PLoS Biol. 2 (11), e363. doi: 10.1371/journal.pbio.0020363 15502875 PMC521178

[B46] Kayama H. Koga R. Atarashi K. Okuyama M. Kimura T. Mak T. W. (2009). NFATc1 mediates toll-like receptor-independent innate immune responses during trypanosoma cruzi infection. PLoS Pathog. 5 (7), e1000514. doi: 10.1371/journal.ppat.1000514 19609356 PMC2704961

[B47] Khachigian L. M. (2006). Early growth response-1 in cardiovascular pathobiology. Circ. Res. 98 (2), 186–191. doi: 10.1161/01.RES.0000200177.53882.c3 16456111

[B48] Ku H. Y. Lin H. (2014). PIWI proteins and their interactors in piRNA biogenesis, germline development and gene expression. Natl. Sci. Rev. 1 (2), 205–218. doi: 10.1093/nsr/nwu014 25512877 PMC4265212

[B49] Kuwahara F. Kai H. Tokuda K. Niiyama H. Tahara N. Kusaba K. (2003). Roles of intercellular adhesion molecule-1 in hypertensive cardiac remodeling. Hypertension 41 (3 Pt 2), 819–823. doi: 10.1161/01.HYP.0000056108.73219.0A 12624002

[B50] Lachmann A. Schilder B. M. Wojciechowicz M. L. Torre D. Kuleshov M. V. Keenan A. B. (2019). Geneshot: search engine for ranking genes from arbitrary text queries. Nucleic Acids Res. 47 (W1), W571–W577. doi: 10.1093/nar/gkz393 31114885 PMC6602493

[B51] Langmead B. Trapnell C. Pop M. Salzberg S. L. (2009). Ultrafast and memory-efficient alignment of short DNA sequences to the human genome. Genome Biol. 10 (3), R25. doi: 10.1186/gb-2009-10-3-r25 19261174 PMC2690996

[B52] Laucella S. Salcedo R. Castanos-Velez E. Riarte A. De Titto E. H. Patarroyo M. (1996). Increased expression and secretion of ICAM-1 during experimental infection with trypanosoma cruzi. Parasite Immunol. 18 (5), 227–239. doi: 10.1046/j.1365-3024.1996.d01-95.x 9229375

[B53] Li R. Frangogiannis N. G. (2021). Chemokines in cardiac fibrosis. Curr. Opin. Physiol. 19, 80–91. doi: 10.1016/j.cophys.2020.10.004 33195890 PMC7665080

[B54] Liehn E. A. Kanzler I. Konschalla S. Kroh A. Simsekyilmaz S. Sonmez T. T. (2013). Compartmentalized protective and detrimental effects of endogenous macrophage migration-inhibitory factor mediated by CXCR2 in a mouse model of myocardial ischemia/reperfusion. Arterioscler. Thromb. Vasc. Biol. 33 (9), 2180–2186. doi: 10.1161/ATVBAHA.113.301633 23868943 PMC4337944

[B55] Lima M. F. Villalta F. (1989). Trypanosoma cruzi trypomastigote clones differentially express a parasite cell adhesion molecule. Mol. Biochem. Parasitol. 33 (2), 159–170. doi: 10.1016/0166-6851(89)90030-3 2657421

[B56] Lino D. O. C. Freitas I. A. Meneses G. C. Martins A. M. C. Daher E. F. Rocha J. H. C. (2019). Interleukin-6 and adhesion molecules VCAM-1 and ICAM-1 as biomarkers of post-acute myocardial infarction heart failure. Braz. J. Med. Biol. Res. 52 (12), e8658. doi: 10.1590/1414-431x20198658 31778438 PMC6886400

[B57] Liu Y. Dou M. Song X. Dong Y. Liu S. Liu H. (2019). The emerging role of the piRNA/piwi complex in cancer. Mol. Cancer 18 (1), 123. doi: 10.1186/s12943-019-1052-9 31399034 PMC6688334

[B58] Luc G. Arveiler D. Evans A. Amouyel P. Ferrieres J. Bard J. M. (2003). Circulating soluble adhesion molecules ICAM-1 and VCAM-1 and incident coronary heart disease: the PRIME study. Atherosclerosis 170 (1), 169–176. doi: 10.1016/S0021-9150(03)00280-6 12957696

[B59] Luo J. Y. Ma Y. T. Xie X. Yang Y. N. Li X. M. Ma X. (2014). Association of intercellular adhesion molecule−1 gene polymorphism with coronary heart disease. Mol. Med. Rep. 10 (3), 1343–1348. doi: 10.3892/mmr.2014.2360 24993975

[B60] Lynn M. K. Bossak B. H. Sandifer P. A. Watson A. Nolan M. S. (2020). Contemporary autochthonous human chagas disease in the USA. Acta Trop. 205, 105361. doi: 10.1016/j.actatropica.2020.105361 32006523

[B61] Majumder S. Kierszenbaum F. (1996). Mechanisms of trypanosoma cruzi-induced down-regulation of lymphocyte function. inhibition of transcription and expression of IL-2 receptor gamma (p64IL-2R) and beta (p70IL-2R) chain molecules in activated normal human lymphocytes. J. Immunol. 156 (10), 3866–3874. 8621925

[B62] Marin-Neto J. A. Cunha-Neto E. Maciel B. C. Simoes M. V. (2007). Pathogenesis of chronic chagas heart disease. Circulation 115 (9), 1109–1123. doi: 10.1161/CIRCULATIONAHA.106.624296 17339569

[B63] Martinez F. Perna E. Perrone S. V. Liprandi A. S. (2019). Chagas disease and heart failure: An expanding issue worldwide. Eur. Cardiol. 14 (2), 82–88. doi: 10.15420/ecr.2018.30.2 31360228 PMC6659042

[B64] Mathieu Bastian S. H. Jacomy. M. (2009). “Gephi: An open source software for exploring and manipulating networks,” in International AAAI Conference on Weblogs and Social Media.

[B65] Matsuda N. M. Miller S. M. Evora P. R. (2009). The chronic gastrointestinal manifestations of chagas disease. Clinics (Sao Paulo) 64 (12), 1219–1224. doi: 10.1590/S1807-59322009001200013 20037711 PMC2797592

[B66] Michailowsky V. Celes M. R. Marino A. P. Silva A. A. Vieira L. Q. Rossi M. A. (2004). Intercellular adhesion molecule 1 deficiency leads to impaired recruitment of T lymphocytes and enhanced host susceptibility to infection with trypanosoma cruzi. J. Immunol. 173 (1), 463–470. doi: 10.4049/jimmunol.173.1.463 15210806

[B67] Moreira C. Batista C. M. Fernandes J. C. Kessler R. L. Soares M. J. Fragoso S. P. (2017). Knockout of the gamma subunit of the AP-1 adaptor complex in the human parasite trypanosoma cruzi impairs infectivity and differentiation and prevents the maturation and targeting of the major protease cruzipain. PLoS One 12 (7), e0179615. doi: 10.1371/journal.pone.0179615 28759609 PMC5536268

[B68] Mott G. A. Costales J. A. Burleigh B. A. (2011). A soluble factor from trypanosoma cruzi inhibits transforming growth factor-ss-induced MAP kinase activation and gene expression in dermal fibroblasts. PLoS One 6 (9), e23482. doi: 10.1371/journal.pone.0023482 21931601 PMC3169535

[B69] Nakagawa N. Barron L. Gomez I. G. Johnson B. G. Roach A. M. Kameoka S. (2016). Pentraxin-2 suppresses c-Jun/AP-1 signaling to inhibit progressive fibrotic disease. JCI Insight 1 (20), e87446. doi: 10.1172/jci.insight.87446 27942582 PMC5135274

[B70] Nandi S. Chandramohan D. Fioriti L. Melnick A. M. Hebert J. M. Mason C. E. (2016). Roles for small noncoding RNAs in silencing of retrotransposons in the mammalian brain. Proc. Natl. Acad. Sci. U.S.A. 113 (45), 12697–12702. doi: 10.1073/pnas.1609287113 27791114 PMC5111663

[B71] Natarajan K. N. Miao Z. Jiang M. Huang X. Zhou H. Xie J. (2019). Comparative analysis of sequencing technologies for single-cell transcriptomics. Genome Biol. 20 (1), 70. doi: 10.1186/s13059-019-1676-5 30961669 PMC6454680

[B72] O'Brien J. Hayder H. Zayed Y. Peng C. (2018). Overview of MicroRNA biogenesis, mechanisms of actions, and circulation. Front. Endocrinol. (Lausanne) 9, 402. doi: 10.3389/fendo.2018.00402 30123182 PMC6085463

[B73] Peng J. C. Lin H. (2013). Beyond transposons: the epigenetic and somatic functions of the piwi-piRNA mechanism. Curr. Opin. Cell Biol. 25 (2), 190–194. doi: 10.1016/j.ceb.2013.01.010 23465540 PMC3651849

[B74] Pinazo M. J. Canas E. Elizalde J. I. Garcia M. Gascon J. Gimeno F. (2010). Diagnosis, management and treatment of chronic chagas' gastrointestinal disease in areas where trypanosoma cruzi infection is not endemic. Gastroenterol. Hepatol. 33 (3), 191–200. doi: 10.1016/j.gastrohep.2009.07.009 19837482

[B75] Pincha N. Hajam E. Y. Badarinath K. Batta S. P. R. Masudi T. Dey R. (2018). PAI1 mediates fibroblast-mast cell interactions in skin fibrosis. J. Clin. Invest. 128 (5), 1807–1819. doi: 10.1172/JCI99088 29584619 PMC5919880

[B76] Pula G. Krause M. (2008). Role of Ena/VASP proteins in homeostasis and disease. Handb. Exp. Pharmacol. 186, 39–65. doi: 10.1007/978-3-540-72843-6_3 18491048

[B77] Py M. O. (2011). Neurologic manifestations of chagas disease. Curr. Neurol. Neurosci. Rep. 11 (6), 536–542. doi: 10.1007/s11910-011-0225-8 21904918

[B78] Rajan K. S. Velmurugan G. Pandi G. Ramasamy S. (2014). miRNA and piRNA mediated akt pathway in heart: Antisense expands to survive. Int. J. Biochem. Cell Biol. 55, 153–156. doi: 10.1016/j.biocel.2014.09.001 25220478

[B79] Rassi A. Jr. Rassi A. Marin-Neto J. A. (2009). Chagas heart disease: Pathophysiologic mechanisms, prognostic factors and risk stratification. Mem Inst Oswaldo Cruz 104 (Suppl 1), 152–158. doi: 10.1590/S0074-02762009000900021 19753470

[B80] Rassi A. Jr. Rassi A. Marin-Neto J. A. (2010). Chagas disease. Lancet 375 (9723), 1388–1402. doi: 10.1016/S0140-6736(10)60061-X 20399979

[B81] Rayford K. J. Cooley A. Arun A. Rachakonda G. Kleschenko Y. Villalta F. (2020). Trypanosoma cruzi modulates PIWI-interacting RNA expression in primary human cardiac myocytes during the early phase of infection. Int. J. Mol. Sci. 21 (24). doi: 10.3390/ijms21249439 PMC7764157 33322418

[B82] Rayford K. J. Cooley A. Arun A. Rachakonda G. Villalta F. Lima M. F. (2021). piRNAs as modulators of disease pathogenesis. Int. J. Mol. Sci. 22 (5). doi: 10.3390/ijms22052373 PMC7956838 33673453

[B83] Rayford K. J. Cooley A. Rumph J. T. Arun A. Rachakonda G. Villalta F. (2022). Trypanosoma cruzi dysregulates expression of piRNA that can regulate IL-6 signaling in human cardiac fibroblasts during the early phase of infection. FASEB J. 36 (S1). doi: 10.1096/fasebj.2022.36.S1.R4785

[B84] Rayner B. S. Figtree G. A. Sabaretnam T. Shang P. Mazhar J. Weaver J. C. (2013). Selective inhibition of the master regulator transcription factor egr-1 with catalytic oligonucleotides reduces myocardial injury and improves left ventricular systolic function in a preclinical model of myocardial infarction. J. Am. Heart Assoc. 2 (4), e000023. doi: 10.1161/JAHA.113.000023 23902638 PMC3828787

[B85] Ricci M. F. Bela S. R. Moraes M. M. Bahia M. T. Mazzeti A. L. Oliveira A. C. S. (2020). Neuronal parasitism, early myenteric neurons depopulation and continuous axonal networking damage as underlying mechanisms of the experimental intestinal chagas' disease. Front. Cell Infect. Microbiol. 10, 583899. doi: 10.3389/fcimb.2020.583899 33178632 PMC7597600

[B86] Richards T. J. Kaminski N. Baribaud F. Flavin S. Brodmerkel C. Horowitz D. (2012). Peripheral blood proteins predict mortality in idiopathic pulmonary fibrosis. Am. J. Respir. Crit. Care Med. 185 (1), 67–76. doi: 10.1164/rccm.201101-0058OC 22016448 PMC3262037

[B87] Rizzo F. Rinaldi A. Marchese G. Coviello E. Sellitto A. Cordella A. (2016). Specific patterns of PIWI-interacting small noncoding RNA expression in dysplastic liver nodules and hepatocellular carcinoma. Oncotarget 7 (34), 54650–54661. doi: 10.18632/oncotarget.10567 27429044 PMC5342370

[B88] Santos-Miranda A. Costa A. D. Joviano-Santos J. V. Rhana P. Bruno A. S. Rocha P. (2021). Inhibition of calcium/calmodulin (Ca(2+) /CaM)-calcium/calmodulin-dependent protein kinase II (CaMKII) axis reduces in vitro and ex vivo arrhythmias in experimental chagas disease. FASEB J. 35 (10), e21901. doi: 10.1096/fj.202101060R 34569665

[B89] Shen J. Xing W. Gong F. Wang W. Yan Y. Zhang Y. (2019). MiR-150-5p retards the progression of myocardial fibrosis by targeting EGR1. Cell Cycle 18 (12), 1335–1348. doi: 10.1080/15384101.2019.1617614 31122130 PMC6592234

[B90] Shimoni Y. Friedlander G. Hetzroni G. Niv G. Altuvia S. Biham O. (2007). Regulation of gene expression by small non-coding RNAs: A quantitative view. Mol. Syst. Biol. 3, 138. doi: 10.1038/msb4100181 17893699 PMC2013925

[B91] Silva T. A. Ferreira L. F. C. Pereira M. C. S. Calvet C. M. (2020). Bearing my heart: The role of extracellular matrix on cardiac development, homeostasis, and injury response. Front. Cell Dev. Biol. 8, 621644. doi: 10.3389/fcell.2020.621644 33511134 PMC7835513

[B92] Silva A. C. Pereira C. Fonseca A. Pinto-do O. P. Nascimento D. S. (2019). Differential role of TGF-beta in extracellular matrix regulation during trypanosoma cruzi-host cell interaction. Int. J. Mol. Sci. 20 (19). doi: 10.20944/preprints201905.0171.v1 PMC6801917 31569452

[B93] Souders C. A. Bowers S. L. Baudino T. A. (2009). Cardiac fibroblast: the renaissance cell. Circ. Res. 105 (12), 1164–1176. doi: 10.1161/CIRCRESAHA.109.209809 19959782 PMC3345531

[B94] Suman S. Rachakonda G. Mandape S. N. Sakhare S. S. Villalta F. Pratap S. (2018). Phospho-proteomic analysis of primary human colon epithelial cells during the early trypanosoma cruzi infection phase. PloS Negl. Trop. Dis. 12 (9), e0006792. doi: 10.1371/journal.pntd.0006792 30222739 PMC6160231

[B95] Suzin J. Respondek A. Respondek M. Bienkiewicz L. Armatys A. (1987). [Prenatal diagnosis of grade III congenital heart block]. Pol. Przegl Radiol. 51 (1), 29–31. 3317299

[B96] Tanowitz H. B. Machado F. S. Jelicks L. A. Shirani J. de Carvalho A. C. Spray D. C. (2009). Perspectives on trypanosoma cruzi-induced heart disease (Chagas disease). Prog. Cardiovasc. Dis. 51 (6), 524–539. doi: 10.1016/j.pcad.2009.02.001 19410685 PMC2677559

[B97] Tarazona S. Furio-Tari P. Turra D. Pietro A. D. Nueda M. J. Ferrer A. (2011). Differential expression in RNA-seq: A matter of depth. Genome Res. 21 (12), 2213–2223. doi: 10.1101/gr.124321.111 21903743 PMC3227109

[B98] Tarazona S. Garcia-Alcalde F. Dopazo J. Ferrer A. Conesa A. (2015). Data quality aware analysis of differential expression in RNA-seq with NOISeq R/Bioc package. Nucleic Acids Res. 43 (21), e140. doi: 10.1093/nar/gkv711 26184878 PMC4666377

[B99] Tarzami S. T. Miao W. Mani K. Lopez L. Factor S. M. Berman J. W. (2003). Opposing effects mediated by the chemokine receptor CXCR2 on myocardial ischemia-reperfusion injury: recruitment of potentially damaging neutrophils and direct myocardial protection. Circulation 108 (19), 2387–2392. doi: 10.1161/01.CIR.0000093192.72099.9A 14568904

[B100] Tsoutsou P. G. Gourgoulianis K. I. Petinaki E. Mpaka M. Efremidou S. Maniatis A. (2004). ICAM-1, ICAM-2 and ICAM-3 in the sera of patients with idiopathic pulmonary fibrosis. Inflammation 28 (6), 359–364. doi: 10.1007/s10753-004-6647-6 16245079

[B101] Tuikue Ndam N. Moussiliou A. Lavstsen T. Kamaliddin C. Jensen A. T. R. Mama A. (2017). Parasites causing cerebral falciparum malaria bind multiple endothelial receptors and express EPCR and ICAM-1-Binding PfEMP1. J. Infect. Dis. 215 (12), 1918–1925. doi: 10.1093/infdis/jix230 28863469

[B102] Udoko A. N. Johnson C. A. Dykan A. Rachakonda G. Villalta F. Mandape S. N. (2016). Early regulation of profibrotic genes in primary human cardiac myocytes by trypanosoma cruzi. PLoS Negl. Trop. Dis. 10 (1), e0003747. doi: 10.1371/journal.pntd.0003747 26771187 PMC4714843

[B103] Villalta F. Lima M. F. Zhou L. (1990). Purification of trypanosoma cruzi surface proteins involved in adhesion to host cells. Biochem. Biophys. Res. Commun. 172 (2), 925–931. doi: 10.1016/0006-291X(90)90764-E 2241980

[B104] Waghabi M. C. Keramidas M. Feige J. J. Araujo-Jorge T. C. Bailly S. (2005). Activation of transforming growth factor beta by trypanosoma cruzi. Cell Microbiol. 7 (4), 511–517. doi: 10.1111/j.1462-5822.2004.00481.x 15760451

[B105] Waghabi M. C. Keramidas M. Calvet C. M. Meuser M. de Nazare C. S. M. Mendonca-Lima L. (2007). SB-431542, a transforming growth factor beta inhibitor, impairs trypanosoma cruzi infection in cardiomyocytes and parasite cycle completion. Antimicrob. Agents Chemother. 51 (8), 2905–2910. doi: 10.1128/AAC.00022-07 17526757 PMC1932517

[B106] Waghabi M. C. de Souza E. M. de Oliveira G. M. Keramidas M. Feige J. J. Araujo-Jorge T. C. (2009). Pharmacological inhibition of transforming growth factor beta signaling decreases infection and prevents heart damage in acute chagas' disease. Antimicrob. Agents Chemother. 53 (11), 4694–4701. doi: 10.1128/AAC.00580-09 19738024 PMC2772341

[B107] Wang C. Lin H. (2021). Roles of piRNAs in transposon and pseudogene regulation of germline mRNAs and lncRNAs. Genome Biol. 22 (1), 27. doi: 10.1186/s13059-020-02221-x 33419460 PMC7792047

[B108] Wang K. Liang C. Liu J. Xiao H. Huang S. Xu J. (2014). Prediction of piRNAs using transposon interaction and a support vector machine. BMC Bioinf. 15, 419. doi: 10.1186/s12859-014-0419-6 PMC4308892 25547961

[B109] Wang L. Zhang Y. L. Lin Q. Y. Liu Y. Guan X. M. Ma X. L. (2018). CXCL1-CXCR2 axis mediates angiotensin II-induced cardiac hypertrophy and remodelling through regulation of monocyte infiltration. Eur. Heart J. 39 (20), 1818–1831. doi: 10.1093/eurheartj/ehy085 29514257

[B110] Warde-Farley D. Donaldson S. L. Comes O. Zuberi K. Badrawi R. Chao P. (2010). The GeneMANIA prediction server: biological network integration for gene prioritization and predicting gene function. Nucleic Acids Res. 38 (Web Server issue), W214–W220. doi: 10.1093/nar/gkq537 20576703 PMC2896186

[B111] Weick E. M. Miska E. A. (2014). piRNAs: from biogenesis to function. Development 141 (18), 3458–3471. doi: 10.1242/dev.094037 25183868

[B112] Wen L. Zhao Z. Li F. Ji F. Wen J. (2022). ICAM-1 related long noncoding RNA is associated with progression of IgA nephropathy and fibrotic changes in proximal tubular cells. Sci. Rep. 12 (1), 9645. doi: 10.1038/s41598-022-13521-6 35688937 PMC9187724

[B113] Weng W. Liu N. Toiyama Y. Kusunoki M. Nagasaka T. Fujiwara T. (2018). Novel evidence for a PIWI-interacting RNA (piRNA) as an oncogenic mediator of disease progression, and a potential prognostic biomarker in colorectal cancer. Mol. Cancer 17 (1), 16. doi: 10.1186/s12943-018-0767-3 29382334 PMC5791351

[B114] Xian S. Chen A. Wu X. Lu C. Wu Y. Huang F. (2021). Activation of activin/Smad2 and 3 signaling pathway and the&nbsp;potential involvement of endothelial−mesenchymal transition in the valvular damage due to rheumatic heart disease. Mol. Med. Rep. 23 (1). doi: 10.3892/mmr.2020.11648 PMC7673319 33179113

[B115] Xuan L. Sun L. Zhang Y. Huang Y. Hou Y. Li Q. (2017). Circulating long non-coding RNAs NRON and MHRT as novel predictive biomarkers of heart failure. J. Cell Mol. Med. 21 (9), 1803–1814. doi: 10.1111/jcmm.13101 28296001 PMC5571539

[B116] Yin D. L. Zhao X. H. Zhou Y. Wang Y. Duan P. Li Q. X. (2019). Association between the ICAM-1 gene polymorphism and coronary heart disease risk: A meta-analysis. Biosci. Rep. 39 (2). doi: 10.1042/BSR20180923 PMC6386762 30674642

[B117] Zago M. P. Wiktorowicz J. E. Spratt H. Koo S. J. Barrientos N. Nunez Burgos A. (2018). Potential utility of protein targets of cysteine-S-Nitrosylation in identifying clinical disease status in human chagas disease. Front. Microbiol. 9, 3320. doi: 10.3389/fmicb.2018.03320 30697201 PMC6340995

[B118] Zhang P. Wang B. J. Wang J. Z. Xie X. M. Tong Q. X. (2020). Association of CX3CL1 and CX3CR1 expression with liver fibrosis in a mouse model of schistosomiasis. Curr. Med. Sci. 40 (6), 1121–1127. doi: 10.1007/s11596-020-2294-x 33428140

[B119] Zhang Y. L. Geng C. Yang J. Fang J. Yan X. Li P. B. (2019). Chronic inhibition of chemokine receptor CXCR2 attenuates cardiac remodeling and dysfunction in spontaneously hypertensive rats. Biochim. Biophys. Acta Mol. Basis Dis. 1865 (12), 165551. doi: 10.1016/j.bbadis.2019.165551 31494226

