# Supplementary material for: Trypanosoma cruzi dysregulates expression profile of piRNAs in primary human cardiac fibroblasts during early infection phase
Source: Front Cell Infect Microbiol. 2023 Mar 2;13:1083379. doi: 10.3389/fcimb.2023.1083379 (PMC10017870; doi:10.3389/fcimb.2023.1083379)
Supplement: Supplementary file 1 [file Table_1.docx]

**Supplementary Table 1: Differential expression values for piRNAs at 1, 3, and 6 hours.**

| piRNA id | Mean expression (Control) | Mean expression (1 hr) | Mean expression (3 hr) | Mean expression (6 hr) | log2Ratio(1 hr) | Probability (1, 1 hr) | log2Ratio(3 hr) | Probability (q, 3hr) | log2Ratio(6 hr) | Probability(q,6 hr) |
| --- | --- | --- | --- | --- | --- | --- | --- | --- | --- | --- |
| hsa_piR_000805 | 0.01 | 17.43333333 | 1.966666667 | 4.136666667 | 10.76763273 | 0.965049929 | 7.619608644 | 0.853165804 | 8.692324899 | 0.909920635 |
| hsa_piR_001356 | 0.033333333 | 11.17 | 13.3 | 20.22 | 8.388447876 | 0.936875892 | 8.640244936 | 0.947647204 | 9.244601688 | 0.965740741 |
| hsa_piR_002468 | 0.066666667 | 16.3 | 6.78 | 14.39 | 7.933690655 | 0.938896814 | 6.668175869 | 0.863759547 | 7.753885283 | 0.941666667 |
| hsa_piR_002485 | 0.29 | 17.6 | 20.97 | 39.25666667 | 5.923378718 | 0.814312886 | 6.176130151 | 0.856245381 | 7.080740966 | 0.939814815 |
| hsa_piR_004150 | 41.50666667 | 15821.66667 | 2141.893333 | 10371.67333 | 8.574342791 | 0.984427009 | 5.689399749 | 0.816580439 | 7.965089881 | 0.982275132 |
| hsa_piR_013624 | 0.14 | 10.71333333 | 11.13666667 | 15.22333333 | 6.257836791 | 0.842011412 | 6.313746845 | 0.852549889 | 6.764713652 | 0.907539683 |
| hsa_piR_016240 | 0.033333333 | 4.85 | 7.663333333 | 6.053333333 | 7.184875343 | 0.877318117 | 7.844862656 | 0.918452821 | 7.504620392 | 0.906878307 |
| hsa_piR_018573 | 0.306666667 | 30.32 | 33.23666667 | 70.70333333 | 6.627454583 | 0.896100808 | 6.75996053 | 0.907982262 | 7.848963062 | 0.969973545 |
| hsa_piR_019949 | 0.043333333 | 26.84333333 | 4.536666667 | 6.03 | 9.2748709 | 0.968021874 | 6.710011633 | 0.848731215 | 7.120536974 | 0.893253968 |
| hsa_piR_020388 | 3.473333333 | 907.89 | 493.7466667 | 1876.28 | 8.030052829 | 0.973371374 | 7.151306326 | 0.950849963 | 9.077338552 | 0.993783069 |
| hsa_piR_020490 | 0.166666667 | 51.16 | 40.70666667 | 43.95 | 8.26190686 | 0.964574417 | 7.932155684 | 0.959719143 | 8.042753761 | 0.967724868 |
| hsa_piR_020496 | 0.203333333 | 103.58 | 24.07333333 | 44.61 | 8.992683006 | 0.981573942 | 6.887445367 | 0.909090909 | 7.777376596 | 0.962962963 |
| hsa_piR_020499 | 0.286666667 | 329.3266667 | 40.49 | 50.04 | 10.16592946 | 0.994412744 | 7.142047674 | 0.93335797 | 7.44756382 | 0.956349206 |
| hsa_piR_020548 | 0.403333333 | 60.38333333 | 59.46333333 | 102.6666667 | 7.226033948 | 0.93913457 | 7.203883887 | 0.939270756 | 7.991779493 | 0.975396825 |
| hsa_piR_020582 | 0.743333333 | 1078.536667 | 162.0866667 | 571.8333333 | 10.5027783 | 0.997028055 | 7.768540399 | 0.967110126 | 9.5873697 | 0.994973545 |
| novel_pir1000 | 0.01 | 4.226666667 | 13.92333333 | 8.863333333 | 8.723376529 | 0.907275321 | 10.44328893 | 0.961320522 | 9.791705561 | 0.94457672 |
| novel_pir1001 | 0.01 | 4.996666667 | 14.25666667 | 16.00666667 | 8.964822167 | 0.916904422 | 10.47742099 | 0.961566888 | 10.64445719 | 0.963888889 |
| novel_pir1002 | 0.01 | 6.04 | 19.54 | 10.51 | 9.238404739 | 0.92617689 | 10.93221475 | 0.967726041 | 10.03754695 | 0.951190476 |
| novel_pir1003 | 0.01 | 2.35 | 6.936666667 | 3.586666667 | 7.876516947 | 0.866737993 | 9.438098749 | 0.934712983 | 8.486499862 | 0.902645503 |
| novel_pir1005 | 0.01 | 1.4 | 3.83 | 4.096666667 | 7.129283017 | 0.820732287 | 8.581200582 | 0.90145356 | 8.6783067 | 0.90978836 |
| novel_pir1007 | 0.01 | 1.343333333 | 4.923333333 | 2.96 | 7.069673528 | 0.81359962 | 8.94349161 | 0.917836906 | 8.209453366 | 0.891269841 |
| novel_pir1010 | 0.01 | 2.896666667 | 7.32 | 5.366666667 | 8.178249866 | 0.883856396 | 9.515699838 | 0.936807095 | 9.067882472 | 0.923412698 |
| novel_pir1012 | 0.01 | 6.18 | 13.68666667 | 12.81666667 | 9.271463028 | 0.927484546 | 10.41855541 | 0.960458241 | 10.32380538 | 0.957407407 |
| novel_pir1013 | 0.01 | 2.753333333 | 7.15 | 5.076666667 | 8.105035471 | 0.879695673 | 9.481799432 | 0.935698448 | 8.987737726 | 0.92010582 |
| novel_pir1018 | 0.01 | 2.636666667 | 6.2 | 6.863333333 | 8.042571384 | 0.876010461 | 9.276124405 | 0.928677014 | 9.422765614 | 0.934126984 |
| novel_pir1019 | 0.01 | 5.253333333 | 15.89333333 | 17.95333333 | 9.037089319 | 0.918568711 | 10.63420602 | 0.963661 | 10.81003601 | 0.966269841 |
| novel_pir1021 | 0.5 | 47.11 | 103.3466667 | 67.10666667 | 6.557961427 | 0.895981931 | 7.691348047 | 0.962305987 | 7.068384192 | 0.946825397 |
| novel_pir1026 | 0.01 | 10.54333333 | 21.27 | 11.9 | 10.04211534 | 0.95042796 | 11.05460432 | 0.970066519 | 10.21674586 | 0.954232804 |
| novel_pir1027 | 0.82 | 61.33 | 115.61 | 109.68 | 6.224825231 | 0.870066572 | 7.139426568 | 0.943089431 | 7.063460851 | 0.94973545 |
| novel_pir1028 | 1.073333333 | 76.97 | 147.44 | 152.9066667 | 6.164126155 | 0.867094627 | 7.101885978 | 0.943335797 | 7.154409311 | 0.95515873 |
| novel_pir1029 | 0.01 | 3.976666667 | 8.533333333 | 8.3 | 8.635415827 | 0.903233476 | 9.736965594 | 0.941980783 | 9.696967526 | 0.942460317 |
| novel_pir1032 | 0.01 | 1.236666667 | 2.96 | 2.443333333 | 6.950312876 | 0.803257252 | 8.209453366 | 0.884700665 | 7.932706887 | 0.874603175 |
| novel_pir1033 | 0.01 | 1.746666667 | 4.06 | 2.066666667 | 7.448460501 | 0.842724679 | 8.665335917 | 0.905395418 | 7.691161905 | 0.862169312 |
| novel_pir1043 | 0.01 | 1.74 | 2.336666667 | 4.88 | 7.442943496 | 0.842605801 | 7.868308133 | 0.867331855 | 8.930737338 | 0.918253968 |
| novel_pir1061 | 0.01 | 1.91 | 3.67 | 1.533333333 | 7.577428828 | 0.850095102 | 8.519636253 | 0.899359448 | 7.26052755 | 0.834391534 |
| novel_pir1065 | 0.01 | 3.146666667 | 3.153333333 | 2.716666667 | 8.297680549 | 0.888730385 | 8.300733873 | 0.889504804 | 8.085693748 | 0.884391534 |
| novel_pir1075 | 4.58 | 0.01 | 0.01 | 0.01 | -8.839203788 | 0.913100333 | -8.839203788 | 0.91438778 | -8.839203788 | 0.914814815 |
| novel_pir110 | 0.01 | 1.273333333 | 1.613333333 | 1.273333333 | 6.992466327 | 0.80658583 | 7.333900737 | 0.833086967 | 6.992466327 | 0.81005291 |
| novel_pir1102 | 0.01 | 4.696666667 | 9.086666667 | 4.733333333 | 8.875493396 | 0.914407989 | 9.827607346 | 0.94506036 | 8.886712714 | 0.916666667 |
| novel_pir1103 | 0.01 | 7.91 | 14.63 | 9.153333333 | 9.627533884 | 0.94020447 | 10.51471405 | 0.961813254 | 9.838153409 | 0.945502646 |
| novel_pir1104 | 0.01 | 4.363333333 | 6.65 | 3.223333333 | 8.769286881 | 0.909771755 | 9.37721053 | 0.932865238 | 8.332409579 | 0.897222222 |
| novel_pir1113 | 0.01 | 2.866666667 | 2.41 | 1.393333333 | 8.163230349 | 0.882905373 | 7.912889336 | 0.870288248 | 7.122396631 | 0.820899471 |
| novel_pir1114 | 0.18 | 11.41666667 | 14.03 | 10.39666667 | 5.987000771 | 0.810152164 | 6.284374292 | 0.858093126 | 5.851980335 | 0.817592593 |
| novel_pir1115 | 0.01 | 15.01333333 | 33.02333333 | 31.50666667 | 10.55202861 | 0.96172135 | 11.68927004 | 0.978073417 | 11.62144141 | 0.978439153 |
| novel_pir1116 | 0.01 | 2.12 | 2.413333333 | 1.99 | 7.727920455 | 0.858773181 | 7.914883386 | 0.870288248 | 7.636624621 | 0.859391534 |
| novel_pir1117 | 0.01 | 8.526666667 | 22.54666667 | 20.78333333 | 9.735838048 | 0.943295292 | 11.13869844 | 0.970682434 | 11.02121134 | 0.97037037 |
| novel_pir1119 | 0.01 | 6.42 | 6.666666667 | 4.986666667 | 9.326429487 | 0.929624346 | 9.380821784 | 0.932865238 | 8.961931959 | 0.919444444 |
| novel_pir112 | 0.01 | 3.46 | 4.326666667 | 4.333333333 | 8.434628228 | 0.894555397 | 8.757112167 | 0.909583641 | 8.759333407 | 0.912169312 |
| novel_pir1120 | 0.26 | 15.82 | 20.34666667 | 25.38333333 | 5.927094166 | 0.817047076 | 6.290137028 | 0.865976842 | 6.609226103 | 0.910846561 |
| novel_pir1121 | 0.01 | 6.376666667 | 9.033333333 | 5.34 | 9.316658658 | 0.929148835 | 9.819114636 | 0.944813994 | 9.060695932 | 0.923148148 |
| novel_pir1122 | 0.01 | 4.66 | 7.12 | 5.456666667 | 8.864186145 | 0.914051355 | 9.475733431 | 0.935575265 | 9.091876106 | 0.924206349 |
| novel_pir1181 | 0.01 | 3.18 | 1.356666667 | 1.21 | 8.312882955 | 0.889205896 | 7.083922484 | 0.810790835 | 6.918863237 | 0.802910053 |
| novel_pir122 | 0.01 | 3.17 | 3.44 | 3.813333333 | 8.30833903 | 0.888968141 | 8.426264755 | 0.895294407 | 8.574908836 | 0.906349206 |
| novel_pir1222 | 0.01 | 1.7 | 2.383333333 | 1.366666667 | 7.409390936 | 0.840109368 | 7.896836931 | 0.868440503 | 7.094517599 | 0.82010582 |
| novel_pir1223 | 1.553333333 | 173.57 | 262.0366667 | 1.303333333 | 6.804006348 | 0.922016167 | 7.398257437 | 0.95959596 | 7.026062297 | 0.812698413 |
| novel_pir1229 | 0.273333333 | 43.52333333 | 65.85666667 | 413.0566667 | 7.314983834 | 0.938777936 | 7.912524273 | 0.963907366 | 8.054828452 | 0.981481481 |
| novel_pir1231 | 0.01 | 3.73 | 5.106666667 | 72.70666667 | 8.54303182 | 0.899548264 | 8.996238081 | 0.919561468 | 8.055282436 | 0.973941799 |
| novel_pir1249 | 0.01 | 1.4 | 1.916666667 | 1.28 | 7.129283017 | 0.820732287 | 7.582455645 | 0.850948509 | 7 | 0.811772487 |
| novel_pir1257 | 0.01 | 28.38333333 | 4.726666667 | 12.82333333 | 11.47082831 | 0.976224441 | 8.884679317 | 0.916481892 | 10.32455561 | 0.957407407 |
| novel_pir1259 | 0.01 | 12.82 | 9.76 | 19.01 | 10.32418055 | 0.957085117 | 9.930737338 | 0.947524021 | 10.89254282 | 0.967724868 |
| novel_pir1263 | 0.22 | 17.31 | 21.55333333 | 29.50333333 | 6.297958391 | 0.860675226 | 6.614263673 | 0.890983001 | 7.067230628 | 0.93505291 |
| novel_pir1264 | 2.38 | 296.1066667 | 312.5433333 | 359.45 | 6.959011589 | 0.932952924 | 7.036950846 | 0.944444444 | 7.23868572 | 0.962698413 |
| novel_pir1267 | 0.633333333 | 56.25666667 | 101.7 | 80.63666667 | 6.472915249 | 0.890870185 | 7.327138951 | 0.95072678 | 6.992327179 | 0.944179894 |
| novel_pir127 | 0.01 | 7.626666667 | 2.473333333 | 2.13 | 9.574908836 | 0.938183547 | 7.950312876 | 0.872998275 | 7.73470962 | 0.864814815 |
| novel_pir128 | 0.01 | 3.733333333 | 5.816666667 | 6.263333333 | 8.544320516 | 0.899548264 | 9.18404882 | 0.926336536 | 9.290786851 | 0.92989418 |
| novel_pir1283 | 0.01 | 39.92333333 | 14.90666667 | 11.37 | 11.96301647 | 0.981930575 | 10.54174197 | 0.962305987 | 10.15101654 | 0.952910053 |
| novel_pir1290 | 0.01 | 7.996666667 | 2.993333333 | 2.81 | 9.643254942 | 0.940561103 | 8.225609134 | 0.885439763 | 8.13442632 | 0.887566138 |
| novel_pir1293 | 0.01 | 3.766666667 | 1.84 | 1.95 | 8.557144557 | 0.899904898 | 7.523561956 | 0.847129835 | 7.607330314 | 0.857539683 |
| novel_pir1302 | 0.01 | 9.99 | 6.156666667 | 8.426666667 | 9.964340868 | 0.948525915 | 9.26600565 | 0.928677014 | 9.718818247 | 0.942724868 |
| novel_pir1303 | 0.01 | 10.04333333 | 4.883333333 | 8.11 | 9.972022457 | 0.948882549 | 8.931722449 | 0.917836906 | 9.663558104 | 0.941005291 |
| novel_pir1324 | 0.01 | 2.013333333 | 2.023333333 | 1.81 | 7.653442239 | 0.855206847 | 7.660590206 | 0.855383099 | 7.499845887 | 0.851058201 |
| novel_pir1328 | 0.01 | 3.236666667 | 5.23 | 4.403333333 | 8.338364985 | 0.890751308 | 9.030667136 | 0.92042375 | 8.782452251 | 0.912698413 |
| novel_pir133 | 0.01 | 8.596666667 | 23.94666667 | 13.43333333 | 9.747633557 | 0.944008559 | 11.22560913 | 0.972283814 | 10.39160162 | 0.959656085 |
| novel_pir1334 | 0.01 | 1.35 | 8.2 | 8.646666667 | 7.076815597 | 0.814907275 | 9.6794801 | 0.940748953 | 9.756000264 | 0.944047619 |
| novel_pir134 | 0.01 | 8.043333333 | 3.876666667 | 3.383333333 | 9.651649699 | 0.940679981 | 8.598672881 | 0.902192658 | 8.402301511 | 0.89973545 |
| novel_pir1351 | 0.01 | 1.55 | 3.19 | 2.086666667 | 7.276124405 | 0.831550166 | 8.317412614 | 0.889997536 | 7.705056346 | 0.863624339 |
| novel_pir1352 | 0.01 | 1.98 | 2.106666667 | 1.26 | 7.62935662 | 0.853185925 | 7.718818247 | 0.859078591 | 6.977279923 | 0.809656085 |
| novel_pir1394 | 0.01 | 1.243333333 | 2.506666667 | 2.613333333 | 6.95806932 | 0.803257252 | 7.969626351 | 0.873860557 | 8.029747343 | 0.880820106 |
| novel_pir1398 | 0.01 | 2.923333333 | 2.843333333 | 5.18 | 8.191470532 | 0.884331907 | 8.151439431 | 0.882606553 | 9.016808288 | 0.921296296 |
| novel_pir1411 | 0.01 | 1.8 | 3.69 | 4.6 | 7.491853096 | 0.845815502 | 8.527477006 | 0.899728997 | 8.845490051 | 0.91494709 |
| novel_pir1429 | 0.01 | 1.56 | 2.203333333 | 1.383333333 | 7.285402219 | 0.832025678 | 7.783543961 | 0.862650899 | 7.112005026 | 0.820634921 |
| novel_pir1441 | 0.01 | 5.523333333 | 3.32 | 17.82666667 | 9.109395387 | 0.922610556 | 8.375039431 | 0.892830746 | 10.79982125 | 0.966137566 |
| novel_pir1445 | 0.01 | 7.733333333 | 4.236666667 | 17.54666667 | 9.594946589 | 0.938540181 | 8.726785814 | 0.907735896 | 10.77698127 | 0.965873016 |
| novel_pir1498 | 0.01 | 1.293333333 | 3.306666667 | 4.016666667 | 7.014950341 | 0.807536852 | 8.36923381 | 0.892461197 | 8.64985493 | 0.908862434 |
| novel_pir1500 | 0.01 | 3.153333333 | 3.156666667 | 4.006666667 | 8.300733873 | 0.888730385 | 8.302258115 | 0.889627987 | 8.64625868 | 0.908862434 |
| novel_pir1501 | 0.01 | 7.173333333 | 12.03 | 11.48666667 | 9.486499862 | 0.936043747 | 10.23242093 | 0.956023651 | 10.16574449 | 0.953306878 |
| novel_pir1516 | 0.01 | 4.686666667 | 7.07 | 4.423333333 | 8.872418378 | 0.914407989 | 9.465566405 | 0.935328899 | 8.788990155 | 0.912962963 |
| novel_pir1569 | 0.01 | 3.406666667 | 1.566666667 | 2.78 | 8.41221698 | 0.89384213 | 7.291554446 | 0.829761025 | 8.118941073 | 0.886772487 |
| novel_pir1573 | 0.01 | 4.226666667 | 3.163333333 | 6.246666667 | 8.723376529 | 0.907275321 | 8.305301776 | 0.889627987 | 9.286942737 | 0.92989418 |
| novel_pir1574 | 0.01 | 7.39 | 4.913333333 | 7.91 | 9.529430554 | 0.937232525 | 8.940558308 | 0.917836906 | 9.627533884 | 0.940343915 |
| novel_pir1575 | 0.286666667 | 86.36666667 | 41.1 | 58.79333333 | 8.23495664 | 0.969567285 | 7.163620425 | 0.934097068 | 7.680134606 | 0.964814815 |
| novel_pir1577 | 0.01 | 3.743333333 | 2.23 | 4.333333333 | 8.548179712 | 0.899667142 | 7.8008999 | 0.863389998 | 8.759333407 | 0.912169312 |
| novel_pir1578 | 0.01 | 1.84 | 1.4 | 1.81 | 7.523561956 | 0.84807418 | 7.129283017 | 0.81584134 | 7.499845887 | 0.851058201 |
| novel_pir1579 | 0.01 | 6.09 | 4.533333333 | 7.45 | 9.250298418 | 0.926414646 | 8.824428435 | 0.914264597 | 9.541096615 | 0.938359788 |
| novel_pir1581 | 0.01 | 2.14 | 1.963333333 | 1.696666667 | 7.741466986 | 0.859248692 | 7.617161323 | 0.852919438 | 7.406559345 | 0.844708995 |
| novel_pir1583 | 0.01 | 2.01 | 4.426666667 | 4.076666667 | 7.651051691 | 0.855206847 | 8.790076931 | 0.91155457 | 8.671246188 | 0.90978836 |
| novel_pir1597 | 0.01 | 3.713333333 | 9.033333333 | 3.716666667 | 8.536571017 | 0.899191631 | 9.819114636 | 0.944813994 | 8.537865494 | 0.904365079 |
| novel_pir1598 | 0.01 | 2.92 | 7.713333333 | 3.736666667 | 8.189824559 | 0.884331907 | 9.591210648 | 0.93902439 | 8.545608062 | 0.904761905 |
| novel_pir1600 | 0.313333333 | 76.09333333 | 244.9766667 | 67.22 | 7.923927996 | 0.962553495 | 9.610730372 | 0.992609017 | 7.745048476 | 0.967857143 |
| novel_pir1601 | 0.22 | 33.76666667 | 61.64333333 | 37.58666667 | 7.261952434 | 0.932477413 | 8.130297543 | 0.967233309 | 7.416573643 | 0.951851852 |
| novel_pir1602 | 0.01 | 3.776666667 | 9.233333333 | 5.013333333 | 8.560969645 | 0.900499287 | 9.85070776 | 0.945429909 | 8.969626351 | 0.919444444 |
| novel_pir1627 | 0.326666667 | 19.61333333 | 18.68 | 20.43666667 | 5.907871687 | 0.81573942 | 5.837531396 | 0.807957625 | 5.967196845 | 0.853306878 |
| novel_pir1635 | 0.01 | 4.573333333 | 6.716666667 | 6.723333333 | 8.837102265 | 0.913100333 | 9.391601623 | 0.933604336 | 9.393032868 | 0.933465608 |
| novel_pir167 | 0.01 | 1.736666667 | 2.52 | 2.016666667 | 7.440177062 | 0.842605801 | 7.977279923 | 0.874599655 | 7.655828831 | 0.860582011 |
| novel_pir1677 | 0.01 | 2.26 | 5.436666667 | 3.373333333 | 7.820178962 | 0.86317166 | 9.086578566 | 0.923503326 | 8.398031074 | 0.89973545 |
| novel_pir168 | 0.01 | 6.826666667 | 13.59333333 | 12.01333333 | 9.415037499 | 0.933309558 | 10.40868356 | 0.960088692 | 10.2304208 | 0.954365079 |
| novel_pir1685 | 0.01 | 3.73 | 3.7 | 4.77 | 8.54303182 | 0.899548264 | 8.531381461 | 0.89985218 | 8.897845456 | 0.917857143 |
| novel_pir1686 | 0.01 | 1.253333333 | 2.396666667 | 3.64 | 6.969626351 | 0.804208274 | 7.90488546 | 0.869918699 | 8.50779464 | 0.903306878 |
| novel_pir1688 | 0.01 | 4.113333333 | 6.74 | 7.86 | 8.684164178 | 0.905492154 | 9.396604781 | 0.933604336 | 9.618385502 | 0.93994709 |
| novel_pir1694 | 0.01 | 2.846666667 | 3.1 | 2.263333333 | 8.153129759 | 0.88254874 | 8.276124405 | 0.889012072 | 7.822305264 | 0.868386243 |
| novel_pir1695 | 0.01 | 1.586666667 | 2.59 | 2.09 | 7.309855263 | 0.833571089 | 8.016808288 | 0.876077852 | 7.707359132 | 0.863624339 |
| novel_pir1698 | 0.01 | 1.386666667 | 2.806666667 | 2.56 | 7.115477217 | 0.818235854 | 8.132713922 | 0.882113821 | 8 | 0.879100529 |
| novel_pir1703 | 1.846666667 | 143.69 | 241.6566667 | 337.5866667 | 6.281892376 | 0.880884451 | 7.03189151 | 0.940872136 | 7.514190639 | 0.970634921 |
| novel_pir1704 | 0.01 | 7.51 | 11.59333333 | 8.233333333 | 9.552669098 | 0.937708036 | 10.17907972 | 0.95516137 | 9.685332826 | 0.942063492 |
| novel_pir1715 | 0.01 | 4.46 | 4.056666667 | 4.01 | 8.8008999 | 0.911198288 | 8.664150952 | 0.905025868 | 8.647458426 | 0.908862434 |
| novel_pir173 | 0.01 | 1.456666667 | 2.593333333 | 1.52 | 7.186526969 | 0.824179743 | 8.018663844 | 0.876077852 | 7.247927513 | 0.833597884 |
| novel_pir186 | 0.01 | 1.503333333 | 1.796666667 | 1.406666667 | 7.232021123 | 0.827864955 | 7.489178962 | 0.844050259 | 7.136136688 | 0.822619048 |
| novel_pir197 | 2.866666667 | 0.01 | 0.01 | 0.01 | -8.163230349 | 0.882905373 | -8.163230349 | 0.882852919 | -8.163230349 | 0.889021164 |
| novel_pir205 | 0.01 | 2.28 | 4.336666667 | 4.333333333 | 7.832890014 | 0.864479315 | 8.760442746 | 0.909583641 | 8.759333407 | 0.912169312 |
| novel_pir212 | 0.01 | 1.273333333 | 2.33 | 1.42 | 6.992466327 | 0.80658583 | 7.864186145 | 0.867085489 | 7.14974712 | 0.823280423 |
| novel_pir221 | 0.01 | 5.02 | 1.766666667 | 1.513333333 | 8.971543554 | 0.9170233 | 7.464886049 | 0.842202513 | 7.241585987 | 0.832936508 |
| novel_pir230 | 0.01 | 1.693333333 | 1.966666667 | 1.246666667 | 7.403722186 | 0.83999049 | 7.619608644 | 0.853165804 | 6.961931959 | 0.808994709 |
| novel_pir241 | 0.01 | 1.256666667 | 1.653333333 | 1.656666667 | 6.973458213 | 0.804446029 | 7.36923381 | 0.835920177 | 7.372139541 | 0.841137566 |
| novel_pir248 | 0.446666667 | 56.10666667 | 82.78333333 | 94.94333333 | 6.972829799 | 0.922848312 | 7.533997936 | 0.955530919 | 7.731724297 | 0.970238095 |
| novel_pir256 | 0.01 | 13.09666667 | 8.09 | 9.723333333 | 10.35498395 | 0.95744175 | 9.659995892 | 0.940133038 | 9.92530717 | 0.947751323 |
| novel_pir281 | 0.01 | 3.033333333 | 4.726666667 | 1.72 | 8.244760234 | 0.886709463 | 8.884679317 | 0.916481892 | 7.426264755 | 0.845238095 |
| novel_pir290 | 0.01 | 17.27666667 | 15.19 | 17.61333333 | 10.75460918 | 0.964931051 | 10.56890615 | 0.962552353 | 10.78245225 | 0.966005291 |
| novel_pir291 | 0.01 | 10.16 | 4.936666667 | 4.333333333 | 9.988684687 | 0.949120304 | 8.947393425 | 0.917836906 | 8.759333407 | 0.912169312 |
| novel_pir293 | 0.01 | 2.556666667 | 1.653333333 | 1.75 | 7.998120267 | 0.874583928 | 7.36923381 | 0.835920177 | 7.451211112 | 0.847619048 |
| novel_pir299 | 0.01 | 5.1 | 3.096666667 | 1.68 | 8.994353437 | 0.917379933 | 8.274572286 | 0.888396157 | 7.392317423 | 0.842989418 |
| novel_pir300 | 0.01 | 20.27666667 | 11.91666667 | 7.42 | 10.98560479 | 0.968497385 | 10.21876503 | 0.955900468 | 9.535275377 | 0.938095238 |
| novel_pir310 | 0.01 | 1.926666667 | 1.273333333 | 1.203333333 | 7.589963182 | 0.850689491 | 6.992466327 | 0.803030303 | 6.910892526 | 0.802248677 |
| novel_pir316 | 0.01 | 9.17 | 4.223333333 | 2.6 | 9.840777924 | 0.945672848 | 8.722238308 | 0.907366346 | 8.022367813 | 0.880555556 |
| novel_pir319 | 0.01 | 4.286666667 | 2.413333333 | 2.406666667 | 8.743712427 | 0.908820732 | 7.914883386 | 0.870288248 | 7.910892526 | 0.872883598 |
| novel_pir320 | 0.01 | 9.316666667 | 7.703333333 | 7.663333333 | 9.863670067 | 0.946267237 | 9.589339044 | 0.93902439 | 9.58182825 | 0.939153439 |
| novel_pir324 | 0.01 | 16.05333333 | 8.906666667 | 7.85 | 10.64865718 | 0.96338564 | 9.798741792 | 0.944444444 | 9.616548844 | 0.93994709 |
| novel_pir327 | 0.01 | 4.423333333 | 3.313333333 | 3.73 | 8.788990155 | 0.910603899 | 8.372139541 | 0.892461197 | 8.54303182 | 0.90462963 |
| novel_pir331 | 0.01 | 9.823333333 | 7.046666667 | 6.55 | 9.940068844 | 0.94828816 | 9.460797161 | 0.935328899 | 9.355351096 | 0.931878307 |
| novel_pir332 | 0.01 | 6.793333333 | 5.95 | 8.223333333 | 9.407975835 | 0.932952924 | 9.216745858 | 0.927322 | 9.6835795 | 0.941798942 |
| novel_pir339 | 0.01 | 5.503333333 | 3.856666667 | 3.9 | 9.104161904 | 0.922372801 | 8.591210648 | 0.901823109 | 8.607330314 | 0.907407407 |
| novel_pir349 | 0.01 | 6.15 | 4.083333333 | 2.89 | 9.2644426 | 0.927127913 | 8.673603533 | 0.905641784 | 8.174925683 | 0.889417989 |
| novel_pir351 | 0.01 | 4.563333333 | 3.48 | 3.183333333 | 8.833944231 | 0.912862577 | 8.442943496 | 0.895910323 | 8.314394422 | 0.895502646 |
| novel_pir399 | 0.01 | 5.78 | 5.863333333 | 9.123333333 | 9.174925683 | 0.92510699 | 9.195577267 | 0.926582902 | 9.833417219 | 0.94537037 |
| novel_pir400 | 0.01 | 6.416666667 | 7.3 | 8.486666667 | 9.32568023 | 0.929624346 | 9.511752654 | 0.936683912 | 9.729054203 | 0.943121693 |
| novel_pir406 | 0.01 | 4.096666667 | 4.27 | 5.59 | 8.6783067 | 0.905373276 | 8.73809226 | 0.907982262 | 9.126704473 | 0.925529101 |
| novel_pir413 | 0.01 | 4.06 | 3.3 | 3.383333333 | 8.665335917 | 0.90466001 | 8.366322214 | 0.892091648 | 8.402301511 | 0.89973545 |
| novel_pir429 | 0.01 | 16.56333333 | 2.836666667 | 4.623333333 | 10.69377733 | 0.963861151 | 8.148052821 | 0.882606553 | 8.852789572 | 0.915079365 |
| novel_pir432 | 0.01 | 7.02 | 2.453333333 | 2.52 | 9.45532722 | 0.934854969 | 7.938599455 | 0.872259177 | 7.977279923 | 0.877116402 |
| novel_pir435 | 0.01 | 2.983333333 | 1.58 | 2.753333333 | 8.220781371 | 0.885877318 | 7.303780748 | 0.829884208 | 8.105035471 | 0.884920635 |
| novel_pir436 | 0.01 | 9.916666667 | 3.493333333 | 4.3 | 9.953711452 | 0.94828816 | 8.448460501 | 0.896279872 | 8.74819285 | 0.911111111 |
| novel_pir438 | 0.01 | 8.02 | 1.746666667 | 3.536666667 | 9.647458426 | 0.940561103 | 7.448460501 | 0.841463415 | 8.46624644 | 0.901587302 |
| novel_pir449 | 0.01 | 6.876666667 | 3.3 | 5.103333333 | 9.425565605 | 0.933547313 | 8.366322214 | 0.892091648 | 8.995296067 | 0.92010582 |
| novel_pir450 | 0.01 | 6.066666667 | 2.21 | 3.173333333 | 9.244760234 | 0.926295768 | 7.787902559 | 0.862897265 | 8.309855263 | 0.89510582 |
| novel_pir462 | 0.01 | 2.083333333 | 1.28 | 1.4 | 7.702749879 | 0.857584403 | 7 | 0.803523035 | 7.129283017 | 0.821428571 |
| novel_pir464 | 0.233333333 | 27.60333333 | 33.99333333 | 21.11 | 6.886306263 | 0.910128388 | 7.186715606 | 0.931263858 | 6.499390347 | 0.901587302 |
| novel_pir465 | 0.01 | 8.53 | 4.293333333 | 4.906666667 | 9.736401931 | 0.943295292 | 8.745954377 | 0.908351811 | 8.938599455 | 0.918783069 |
| novel_pir467 | 0.01 | 6.913333333 | 22.95333333 | 5.94 | 9.433237678 | 0.933547313 | 11.16448796 | 0.971051983 | 9.214319121 | 0.928174603 |
| novel_pir468 | 0.01 | 7.016666667 | 5.486666667 | 6.956666667 | 9.454642017 | 0.934854969 | 9.09978612 | 0.923872875 | 9.442252384 | 0.93478836 |
| novel_pir471 | 0.01 | 2.45 | 2.096666667 | 3.286666667 | 7.936637939 | 0.871136472 | 7.711953706 | 0.858832225 | 8.360481336 | 0.897751323 |
| novel_pir472 | 1.446666667 | 131.7566667 | 132.3366667 | 161.5266667 | 6.508999609 | 0.902282454 | 6.515336494 | 0.906996797 | 6.802896009 | 0.938624339 |
| novel_pir476 | 0.01 | 1.75 | 1.723333333 | 1.67 | 7.451211112 | 0.842724679 | 7.42905797 | 0.839122937 | 7.383704292 | 0.842063492 |
| novel_pir479 | 0.01 | 3.866666667 | 3.483333333 | 3.263333333 | 8.594946589 | 0.902044698 | 8.444324726 | 0.895910323 | 8.350202549 | 0.897486772 |
| novel_pir481 | 0.01 | 2.103333333 | 3.423333333 | 3.23 | 7.716533694 | 0.858535426 | 8.419257966 | 0.894678492 | 8.335390355 | 0.897354497 |
| novel_pir504 | 0.7 | 100.2066667 | 68.23666667 | 86.18 | 7.161407856 | 0.940679981 | 6.607048441 | 0.906873614 | 6.943854366 | 0.942063492 |
| novel_pir507 | 0.38 | 47.95333333 | 41.5 | 51.82 | 6.979487875 | 0.922491679 | 6.770968108 | 0.912293668 | 7.091365787 | 0.94457672 |
| novel_pir508 | 0.01 | 2.58 | 1.413333333 | 1.57 | 8.011227255 | 0.874821683 | 7.142957954 | 0.816210889 | 7.294620749 | 0.836640212 |
| novel_pir512 | 0.153333333 | 26.35333333 | 18.01 | 20.17666667 | 7.425170284 | 0.935211602 | 6.875983011 | 0.903178123 | 7.03987268 | 0.927380952 |
| novel_pir518 | 1.913333333 | 344.15 | 212.12 | 198.0033333 | 7.490805464 | 0.95851165 | 6.792648607 | 0.927322 | 6.693292671 | 0.935714286 |
| novel_pir519 | 0.01 | 15.22 | 7.883333333 | 9.243333333 | 10.57175264 | 0.962196862 | 9.622661968 | 0.939763489 | 9.8522694 | 0.945767196 |
| novel_pir522 | 0.01 | 9.003333333 | 3.49 | 3.966666667 | 9.814315424 | 0.944840704 | 8.447083226 | 0.896279872 | 8.631783357 | 0.908465608 |
| novel_pir523 | 0.01 | 10.39 | 6.756666667 | 6.626666667 | 10.02097994 | 0.949714693 | 9.400167873 | 0.933604336 | 9.372139541 | 0.932275132 |
| novel_pir525 | 1.593333333 | 221.19 | 152.7233333 | 179.0933333 | 7.117094235 | 0.94234427 | 6.582728569 | 0.910815472 | 6.812519707 | 0.941137566 |
| novel_pir528 | 2.6 | 277.4 | 212.45 | 247.1833333 | 6.737312354 | 0.923442701 | 6.35246791 | 0.897265336 | 6.570926037 | 0.930952381 |
| novel_pir529 | 2.22 | 262.1933333 | 182.99 | 206.3966667 | 6.883927516 | 0.929386591 | 6.365061324 | 0.896649421 | 6.538716184 | 0.929100529 |
| novel_pir533 | 0.01 | 5.43 | 7.646666667 | 6.696666667 | 9.084808388 | 0.921540656 | 9.578687175 | 0.938408475 | 9.387299348 | 0.932936508 |
| novel_pir536 | 0.3 | 20.40333333 | 20.69333333 | 17.10666667 | 6.087698556 | 0.839396101 | 6.108059746 | 0.847868933 | 5.833452359 | 0.830820106 |
| novel_pir537 | 0.01 | 2.466666667 | 1.546666667 | 2.033333333 | 7.94641896 | 0.871968616 | 7.273018494 | 0.826804632 | 7.667702932 | 0.860582011 |
| novel_pir538 | 0.01 | 20.70333333 | 16.10333333 | 19.16666667 | 11.01564735 | 0.968854018 | 10.65314364 | 0.963784183 | 10.90438374 | 0.967724868 |
| novel_pir540 | 0.01 | 1.513333333 | 1.833333333 | 1.55 | 7.241585987 | 0.828578222 | 7.518325308 | 0.846267554 | 7.276124405 | 0.83505291 |
| novel_pir542 | 0.01 | 15.06333333 | 10.04666667 | 11.94666667 | 10.55682534 | 0.961840228 | 9.972501201 | 0.949371766 | 10.22239242 | 0.954232804 |
| novel_pir566 | 0.54 | 49.63 | 49.99666667 | 42.4 | 6.522109237 | 0.894436519 | 6.532728695 | 0.898866716 | 6.294961047 | 0.898809524 |
| novel_pir569 | 0.01 | 2.91 | 2.04 | 2.236666667 | 8.184875343 | 0.884094151 | 7.672425342 | 0.856368564 | 7.805206455 | 0.867857143 |
| novel_pir573 | 0.233333333 | 24.2 | 19.42 | 18.36 | 6.696470816 | 0.895863053 | 6.379006969 | 0.870534614 | 6.298029827 | 0.883068783 |
| novel_pir579 | 0.01 | 4.093333333 | 3.113333333 | 3.61 | 8.677132345 | 0.905254398 | 8.282316239 | 0.889135255 | 8.495855027 | 0.903042328 |
| novel_pir580 | 0.646666667 | 75.44 | 91.36333333 | 79.92666667 | 6.866163619 | 0.92189729 | 7.142449231 | 0.940748953 | 6.949510866 | 0.943518519 |
| novel_pir581 | 0.01 | 2.44 | 2.763333333 | 2.423333333 | 7.930737338 | 0.870898716 | 8.110265791 | 0.88125154 | 7.920849053 | 0.873280423 |
| novel_pir583 | 0.01 | 2.913333333 | 3.29 | 2.733333333 | 8.186526969 | 0.884094151 | 8.361943774 | 0.892091648 | 8.094517599 | 0.884391534 |
| novel_pir586 | 0.01 | 3.323333333 | 4.906666667 | 5.116666667 | 8.376487194 | 0.892415597 | 8.938599455 | 0.917836906 | 8.99906044 | 0.920634921 |
| novel_pir587 | 1.286666667 | 91.92 | 139.63 | 148.7766667 | 6.158668546 | 0.868758916 | 6.761826786 | 0.922641045 | 6.853366123 | 0.941534392 |
| novel_pir590 | 0.01 | 10.83333333 | 13.55 | 16.86333333 | 10.0812615 | 0.951141227 | 10.40407714 | 0.959965509 | 10.71967402 | 0.964814815 |
| novel_pir647 | 0.3 | 31.38666667 | 43.19333333 | 52.03333333 | 6.709045509 | 0.900618165 | 7.169702346 | 0.935205716 | 7.438329821 | 0.957142857 |
| novel_pir655 | 0.01 | 26.23 | 88.27666667 | 23.59 | 11.35700209 | 0.975035663 | 13.10781644 | 0.989283075 | 11.2039597 | 0.972751323 |
| novel_pir656 | 0.01 | 8.15 | 20.50666667 | 8.723333333 | 9.670656249 | 0.941036614 | 11.00187729 | 0.969081054 | 9.768735708 | 0.944179894 |
| novel_pir658 | 0.01 | 4.59 | 13.35333333 | 3.386666667 | 8.842350343 | 0.913219211 | 10.38298421 | 0.95922641 | 8.403722186 | 0.899867725 |
| novel_pir665 | 1.496666667 | 0.01 | 0.01 | 0.01 | -7.225609134 | 0.827864955 | -7.225609134 | 0.823971422 | -7.225609134 | 0.831216931 |
| novel_pir68 | 0.01 | 8.036666667 | 11.31666667 | 15.05 | 9.650453435 | 0.940679981 | 10.14423336 | 0.954422271 | 10.55554777 | 0.962830688 |
| novel_pir686 | 0.01 | 6.026666667 | 5.093333333 | 10.35666667 | 9.235216462 | 0.92617689 | 8.992466327 | 0.919315102 | 10.01634403 | 0.950925926 |
| novel_pir715 | 0.01 | 2.336666667 | 1.43 | 1.746666667 | 7.868308133 | 0.866500238 | 7.159871337 | 0.818058635 | 7.448460501 | 0.847619048 |
| novel_pir722 | 0.01 | 5.65 | 1.686666667 | 1.41 | 9.142107057 | 0.923799334 | 7.398031074 | 0.838014289 | 7.139551352 | 0.822619048 |
| novel_pir723 | 0.01 | 13.13333333 | 6.63 | 4.786666667 | 10.35901741 | 0.957560628 | 9.37286506 | 0.932495689 | 8.902877533 | 0.917857143 |
| novel_pir725 | 0.01 | 8.683333333 | 5.426666667 | 3.4 | 9.762105156 | 0.944127437 | 9.083922484 | 0.92325696 | 8.409390936 | 0.899867725 |
| novel_pir727 | 0.01 | 4.07 | 2.596666667 | 3.056666667 | 8.668884984 | 0.90466001 | 8.020517017 | 0.876324218 | 8.255815423 | 0.893253968 |
| novel_pir731 | 0.01 | 18.67 | 9.473333333 | 7.88 | 10.86650621 | 0.966357584 | 9.887728339 | 0.946169007 | 9.622051819 | 0.94021164 |
| novel_pir735 | 0.01 | 11.55 | 7.913333333 | 4.986666667 | 10.17367714 | 0.953281027 | 9.628141719 | 0.939763489 | 8.961931959 | 0.919444444 |
| novel_pir739 | 0.01 | 2.883333333 | 3.936666667 | 1.486666667 | 8.171593822 | 0.883024251 | 8.620830749 | 0.902931757 | 7.215937399 | 0.829761905 |
| novel_pir741 | 0.01 | 3.956666667 | 1.316666667 | 1.513333333 | 8.628141719 | 0.903233476 | 7.040746342 | 0.808450357 | 7.241585987 | 0.832936508 |
| novel_pir744 | 0.01 | 5.496666667 | 4.463333333 | 2.56 | 9.102413183 | 0.922372801 | 8.801977745 | 0.912047302 | 8 | 0.879100529 |
| novel_pir788 | 0.01 | 1.916666667 | 1.646666667 | 2.033333333 | 7.582455645 | 0.850451736 | 7.363404731 | 0.835796994 | 7.667702932 | 0.860582011 |
| novel_pir810 | 0.01 | 3.813333333 | 4.186666667 | 5.226666667 | 8.574908836 | 0.90085592 | 8.709658248 | 0.906873614 | 9.029747343 | 0.921825397 |
| novel_pir818 | 0.01 | 4.856666667 | 1.98 | 3.706666667 | 8.923822661 | 0.916428911 | 7.62935662 | 0.853535354 | 8.533978572 | 0.903968254 |
| novel_pir855 | 0.01 | 3.29 | 5.986666667 | 4.393333333 | 8.361943774 | 0.89170233 | 9.225609134 | 0.927445184 | 8.779172154 | 0.912566138 |
| novel_pir856 | 0.313333333 | 27.86333333 | 47.87 | 38.42333333 | 6.474525794 | 0.881716595 | 7.25527974 | 0.939886672 | 6.938140616 | 0.932936508 |
| novel_pir858 | 0.01 | 1.34 | 2.39 | 1.38 | 7.06608919 | 0.81359962 | 7.900866808 | 0.869425967 | 7.108524457 | 0.820634921 |
| novel_pir873 | 0.01 | 3.516666667 | 5.056666667 | 6.59 | 8.458064783 | 0.896457442 | 8.98204287 | 0.919191919 | 9.364134655 | 0.931878307 |
| novel_pir918 | 0.01 | 4.53 | 3.43 | 4.32 | 8.82336724 | 0.912505944 | 8.422064766 | 0.894924858 | 8.754887502 | 0.911640212 |
| novel_pir921 | 0.01 | 2.08 | 2.943333333 | 1.543333333 | 7.700439718 | 0.857584403 | 8.201307127 | 0.884454299 | 7.269905883 | 0.834920635 |
| novel_pir934 | 0.01 | 2.046666667 | 2.506666667 | 2.18 | 7.677132345 | 0.856990014 | 7.969626351 | 0.873860557 | 7.768184325 | 0.865873016 |
| novel_pir946 | 0.01 | 2.306666667 | 2.76 | 2.556666667 | 7.849665727 | 0.865786971 | 8.108524457 | 0.88125154 | 7.998120267 | 0.879100529 |
| novel_pir950 | 0.01 | 6.7 | 6.993333333 | 6.956666667 | 9.388017285 | 0.93212078 | 9.449836462 | 0.93495935 | 9.442252384 | 0.93478836 |
| novel_pir952 | 0.01 | 3.603333333 | 7.52 | 8.446666667 | 8.493188307 | 0.898121731 | 9.554588852 | 0.938162109 | 9.722238308 | 0.942857143 |
| novel_pir954 | 0.01 | 2.74 | 4.433333333 | 2.776666667 | 8.098032083 | 0.879576795 | 8.79224803 | 0.91155457 | 8.117210185 | 0.886772487 |
| novel_pir955 | 0.153333333 | 10.66666667 | 26.64333333 | 26.15333333 | 6.120294234 | 0.824060865 | 7.440959418 | 0.93902439 | 7.414179664 | 0.945767196 |
| novel_pir960 | 1.5 | 76.53 | 115.52 | 98.56666667 | 5.672990994 | 0.805872563 | 6.267036336 | 0.887041143 | 6.038065432 | 0.883597884 |
| novel_pir962 | 0.01 | 1.26 | 1.41 | 3.666666667 | 6.977279923 | 0.805872563 | 7.139551352 | 0.816210889 | 8.518325308 | 0.903703704 |
| novel_pir969 | 0.526666667 | 36.97333333 | 75.17 | 58.43666667 | 6.133451153 | 0.859129815 | 7.157123041 | 0.939393939 | 6.793839924 | 0.930952381 |
| novel_pir971 | 0.01 | 7.95 | 5 | 11.89333333 | 9.63481105 | 0.940323348 | 8.965784285 | 0.918329638 | 10.2159374 | 0.954100529 |
| novel_pir972 | 0.01 | 1.216666667 | 2.43 | 2.553333333 | 6.926790153 | 0.800523062 | 7.924812504 | 0.87115053 | 7.996238081 | 0.879100529 |
| novel_pir973 | 1.386666667 | 75.30333333 | 154.6633333 | 171.1666667 | 5.763020795 | 0.811697575 | 6.801366374 | 0.924611973 | 6.947636938 | 0.948148148 |
| novel_pir976 | 0.18 | 11.04666667 | 28.79 | 29.93333333 | 5.939470385 | 0.807061341 | 7.321427072 | 0.93495935 | 7.377612227 | 0.946693122 |
| novel_pir977 | 2.476666667 | 128.96 | 310.8333333 | 233.55 | 5.702380129 | 0.811816453 | 6.971597704 | 0.939270756 | 6.559187925 | 0.929232804 |
| novel_pir978 | 0.01 | 3.516666667 | 9.923333333 | 10.85666667 | 8.458064783 | 0.896457442 | 9.954681006 | 0.948386302 | 10.0843655 | 0.951851852 |
| novel_pir980 | 0.01 | 3.436666667 | 7.496666667 | 10.34666667 | 8.424866117 | 0.894198764 | 9.550105445 | 0.937915743 | 10.01495034 | 0.950925926 |
| novel_pir981 | 0.01 | 4.473333333 | 10.15 | 9.006666667 | 8.805206455 | 0.911198288 | 9.987264012 | 0.949864499 | 9.814849459 | 0.94484127 |
| novel_pir982 | 0.01 | 6.453333333 | 5.183333333 | 11.87666667 | 9.333900737 | 0.929624346 | 9.017736364 | 0.92042375 | 10.21391427 | 0.954100529 |
| novel_pir984 | 1.613333333 | 98.63333333 | 318.0533333 | 316.58 | 5.933958839 | 0.842724679 | 7.62308035 | 0.96809559 | 7.616381759 | 0.973677249 |
| novel_pir996 | 0.01 | 3.396666667 | 9.756666667 | 5.243333333 | 8.407975835 | 0.89384213 | 9.93024453 | 0.947524021 | 9.034340455 | 0.922222222 |
| novel_pir998 | 0.01 | 6.66 | 19.38333333 | 28.10666667 | 9.379378367 | 0.931883024 | 10.92060098 | 0.967479675 | 11.45669665 | 0.976851852 |
| novel_pir999 | 0.01 | 6.04 | 17.17333333 | 10.38333333 | 9.238404739 | 0.92617689 | 10.74595438 | 0.965016014 | 10.02005395 | 0.950925926 |
| novel_pir1098 | 0.01 | 2.74 | 2.57 | NA | 8.098032083 | 0.879576795 | 8.005624549 | 0.875585119 | NA | NA |
| novel_pir1099 | 0.01 | 1.233333333 | 1.386666667 | NA | 6.94641896 | 0.802068474 | 7.115477217 | 0.815102242 | NA | NA |
| novel_pir1100 | 0.01 | 1.823333333 | 1.903333333 | NA | 7.510434522 | 0.846528768 | 7.572384435 | 0.849716679 | NA | NA |
| novel_pir1101 | 0.01 | 1.276666667 | 2.063333333 | NA | 6.996238081 | 0.807299097 | 7.688833098 | 0.856738113 | NA | NA |
| novel_pir1183 | 0.01 | 2.06 | 1.783333333 | NA | 7.686500527 | 0.857108892 | 7.478432581 | 0.843557526 | NA | NA |
| novel_pir1275 | 0.01 | 1.466666667 | 1.72 | NA | 7.196397213 | 0.824417499 | 7.426264755 | 0.839122937 | NA | NA |
| novel_pir1292 | 0.01 | 2.543333333 | 2.443333333 | NA | 7.990576746 | 0.874227294 | 7.932706887 | 0.872012811 | NA | NA |
| novel_pir1294 | 0.01 | 1.51 | 1.373333333 | NA | 7.238404739 | 0.828578222 | 7.101538026 | 0.813624045 | NA | NA |
| novel_pir17 | 0.01 | 3.853333333 | 1.976666667 | NA | 8.589963182 | 0.901806942 | 7.626925794 | 0.853535354 | NA | NA |
| novel_pir296 | 0.606666667 | 43.91 | 32.12666667 | NA | 6.17750168 | 0.861745126 | 5.726723446 | 0.804878049 | NA | NA |
| novel_pir329 | 0.166666667 | 15.00666667 | 10.4 | NA | 6.492494152 | 0.871017594 | 5.963474124 | 0.818305001 | NA | NA |
| novel_pir509 | 0.01 | 2.22 | 1.26 | NA | 7.794415866 | 0.861745126 | 6.977279923 | 0.801552106 | NA | NA |
| novel_pir749 | 0.01 | 2.233333333 | 1.72 | NA | 7.803054785 | 0.862101759 | 7.426264755 | 0.839122937 | NA | NA |
| novel_pir822 | 0.01 | 2.096666667 | 1.703333333 | NA | 7.711953706 | 0.858535426 | 7.41221698 | 0.838630204 | NA | NA |
| hsa_piR_000753 | 0.066666667 |  | NA | 16.46 | 6.167919866 | 0.805159296 | NA | NA | 7.947783026 | 0.947354497 |
| hsa_piR_004987 | 0.726666667 |  | NA | 38.99666667 | 5.805817813 | 0.820256776 | NA | NA | 5.745913272 | 0.836375661 |
| hsa_piR_016742 | 6.123333333 |  | NA | 584.3733333 | 7.909355808 | 0.971825963 | NA | NA | 6.576429314 | 0.932936508 |
| hsa_piR_016828 | 0.353333333 |  | NA | 30.28333333 | 7.221267916 | 0.937589158 | NA | NA | 6.421350345 | 0.901851852 |
| hsa_piR_017716 | 0.02 |  | NA | 7.536666667 | 8.329422631 | 0.918330956 | NA | NA | 8.557782776 | 0.932010582 |
| hsa_piR_019825 | 0.043333333 |  | NA | 64.25666667 | 10.4730032 | 0.985140276 | NA | NA | 10.53415321 | 0.987830688 |
| hsa_piR_020391 | 1.07 |  | NA | 79.98666667 | 6.117873668 | 0.85829767 | NA | NA | 6.224076829 | 0.898280423 |
| novel_pir1397 | 0.01 |  | NA | 2.126666667 | 6.942514505 | 0.802068474 | NA | NA | 7.732450113 | 0.864814815 |
| novel_pir1468 | 0.01 |  | NA | 1.496666667 | 6.981091537 | 0.805872563 | NA | NA | 7.225609134 | 0.831216931 |
| novel_pir149 | 0.01 |  | NA | 1.213333333 | 6.984893108 | 0.80658583 | NA | NA | 6.922832139 | 0.802910053 |
| novel_pir1496 | 0.01 |  | NA | 1.656666667 | 7.415037499 | 0.840228245 | NA | NA | 7.372139541 | 0.841137566 |
| novel_pir1572 | 0.01 |  | NA | 2.353333333 | 8.490516649 | 0.898121731 | NA | NA | 7.878561873 | 0.871428571 |
| novel_pir330 | 0.01 |  | NA | 2.203333333 | 7.453956489 | 0.842724679 | NA | NA | 7.783543961 | 0.866798942 |
| novel_pir357 | 0.01 |  | NA | 3.003333333 | 7.324930583 | 0.834403233 | NA | NA | 8.230420795 | 0.892857143 |
| novel_pir358 | 0.01 |  | NA | 3 | 7.17990909 | 0.823704232 | NA | NA | 8.22881869 | 0.892857143 |
| novel_pir359 | 0.01 |  | NA | 10.51666667 | 9.138698444 | 0.923680456 | NA | NA | 10.03846179 | 0.951190476 |
| novel_pir372 | 0.01 |  | NA | 2.8 | 7.866248611 | 0.866500238 | NA | NA | 8.129283017 | 0.887301587 |
| novel_pir381 | 0.01 |  | NA | 4.786666667 | 7.940558308 | 0.871493105 | NA | NA | 8.902877533 | 0.917857143 |
| novel_pir383 | 0.01 |  | NA | 2.17 | 7.574908836 | 0.849976224 | NA | NA | 7.761551232 | 0.865343915 |
| novel_pir386 | 0.01 |  | NA | 20.81333333 | 8.941536738 | 0.916666667 | NA | NA | 11.02329232 | 0.97037037 |
| novel_pir388 | 0.01 |  | NA | 6.563333333 | 9.596810938 | 0.938540181 | NA | NA | 9.358284895 | 0.931878307 |
| novel_pir390 | 0.01 |  | NA | 1.316666667 | 7.453956489 | 0.842724679 | NA | NA | 7.040746342 | 0.816269841 |
| novel_pir415 | 0.01 |  | NA | 2.763333333 | 8.314394422 | 0.889205896 | NA | NA | 8.110265791 | 0.885582011 |
| novel_pir420 | 0.01 |  | NA | 3.886666667 | 8.159871337 | 0.882786495 | NA | NA | 8.602389572 | 0.907407407 |
| novel_pir426 | 0.01 |  | NA | 1.603333333 | 7.604862058 | 0.85128388 | NA | NA | 7.324930583 | 0.839021164 |
| novel_pir439 | 0.01 |  | NA | 1.24 | 7.033423002 | 0.811103186 | NA | NA | 6.95419631 | 0.808333333 |
| novel_pir445 | 0.01 |  | NA | 1.24 | 7.932706887 | 0.870898716 | NA | NA | 6.95419631 | 0.808333333 |
| novel_pir457 | 6.216666667 |  | NA | 280.9233333 | 6.373612761 | 0.894674275 | NA | NA | 5.497891424 | 0.807142857 |
| novel_pir545 | 0.01 |  | NA | 1.203333333 | 7.285402219 | 0.832025678 | NA | NA | 6.910892526 | 0.802248677 |
| novel_pir605 | 0.01 |  | NA | 1.606666667 | 7.533978572 | 0.848193058 | NA | NA | 7.327926836 | 0.839550265 |
| novel_pir606 | 0.01 |  | NA | 1.193333333 | 7.982993575 | 0.874227294 | NA | NA | 6.898853277 | 0.801851852 |
| novel_pir614 | 0.01 |  | NA | 1.98 | 7.464886049 | 0.843556824 | NA | NA | 7.62935662 | 0.858994709 |
| novel_pir615 | 0.01 |  | NA | 1.976666667 | 8.417852515 | 0.894079886 | NA | NA | 7.626925794 | 0.858994709 |
| novel_pir718 | 0.01 |  | NA | 1.876666667 | 7.705056346 | 0.857584403 | NA | NA | 7.552028611 | 0.853439153 |
| novel_pir719 | 0.193333333 |  | NA | 20.72333333 | 7.035699743 | 0.918568711 | NA | NA | 6.744021868 | 0.914153439 |
| novel_pir720 | 0.01 |  | NA | 1.843333333 | 7.912889336 | 0.869472183 | NA | NA | 7.52617317 | 0.851851852 |
| novel_pir734 | 0.01 |  | NA | 2.39 | 7.843397672 | 0.865786971 | NA | NA | 7.900866808 | 0.872222222 |
| novel_pir1006 | 0.01 | NA | 1.936666667 | 1.63 | NA | NA | 7.597431853 | 0.851933974 | 7.348728154 | 0.840079365 |
| novel_pir1009 | 0.01 | NA | 2.663333333 | 1.43 | NA | NA | 8.057089192 | 0.87804878 | 7.159871337 | 0.823544974 |
| novel_pir1015 | 0.01 | NA | 3.516666667 | 1.963333333 | NA | NA | 8.458064783 | 0.896403055 | 7.617161323 | 0.858068783 |
| novel_pir1020 | 0.01 | NA | 1.673333333 | 1.443333333 | NA | NA | 7.386581053 | 0.83641291 | 7.173260714 | 0.824338624 |
| novel_pir1023 | 0.26 | NA | 25.53666667 | 17.55333333 | NA | NA | 6.617914787 | 0.893446662 | 6.077089587 | 0.862037037 |
| novel_pir1045 | 0.01 | NA | 1.26 | 3.05 | NA | NA | 6.977279923 | 0.801552106 | 8.252665432 | 0.893253968 |
| novel_pir1063 | 0.01 | NA | 1.263333333 | 2.663333333 | NA | NA | 6.981091537 | 0.801552106 | 8.057089192 | 0.881613757 |
| novel_pir1111 | 0.01 | NA | 1.98 | 1.883333333 | NA | NA | 7.62935662 | 0.853535354 | 7.557144557 | 0.853439153 |
| novel_pir1118 | 0.553333333 | NA | 46.12333333 | 60.37333333 | NA | NA | 6.381204134 | 0.886178862 | 6.769618811 | 0.931349206 |
| novel_pir1217 | 0.01 | NA | 1.666666667 | 1.17 | NA | NA | 7.380821784 | 0.83641291 | 6.87036472 | 0.800529101 |
| novel_pir1223 | 0.01 | NA | 1.796666667 | 1.303333333 | NA | NA | 7.489178962 | 0.844050259 | 7.026062297 | 0.812698413 |
| novel_pir1244 | 0.01 | NA | 1.79 | 1.446666667 | NA | NA | 7.483815777 | 0.84368071 | 7.176588732 | 0.82526455 |
| novel_pir1338 | 0.01 | NA | 2.36 | 1.29 | NA | NA | 7.882643049 | 0.868070953 | 7.011227255 | 0.812037037 |
| novel_pir1396 | 0.01 | NA | 1.476666667 | 1.876666667 | NA | NA | 7.206200388 | 0.822985957 | 7.552028611 | 0.853439153 |
| novel_pir1497 | 0.166666667 | NA | 14.40333333 | 19.03 | NA | NA | 6.433293326 | 0.871889628 | 6.835166157 | 0.915079365 |
| novel_pir1603 | 0.01 | NA | 1.363333333 | 1.703333333 | NA | NA | 7.090994532 | 0.8117763 | 7.41221698 | 0.844708995 |
| novel_pir166 | 0.01 | NA | 1.69 | 2.49 | NA | NA | 7.400879436 | 0.838014289 | 7.960001932 | 0.876322751 |
| novel_pir1705 | 0.206666667 | NA | 14.52 | 22.22 | NA | NA | 6.134591928 | 0.842448879 | 6.748409292 | 0.915079365 |
| novel_pir1707 | 0.153333333 | NA | 17.99333333 | 27.98333333 | NA | NA | 6.874647305 | 0.903178123 | 7.511752654 | 0.949867725 |
| novel_pir1713 | 0.353333333 | NA | 29.99666667 | 25.04666667 | NA | NA | 6.407628523 | 0.881621089 | 6.147444946 | 0.877116402 |
| novel_pir35 | 0.01 | NA | 1.68 | 1.32 | NA | NA | 7.392317423 | 0.837275191 | 7.044394119 | 0.816269841 |
| novel_pir405 | 0.01 | NA | 1.46 | 1.516666667 | NA | NA | 7.189824559 | 0.82027593 | 7.244760234 | 0.833465608 |
| novel_pir51 | 0.446666667 | NA | 51.42 | 26.94 | NA | NA | 6.846987205 | 0.919191919 | 5.914407446 | 0.850529101 |
| novel_pir659 | 0.01 | NA | 1.543333333 | 1.306666667 | NA | NA | 7.269905883 | 0.826804632 | 7.029747343 | 0.813888889 |
| novel_pir660 | 0.01 | NA | 2.273333333 | 1.433333333 | NA | NA | 7.828665428 | 0.865237743 | 7.163230349 | 0.823544974 |
| novel_pir706 | 0.01 | NA | 1.64 | 1.483333333 | NA | NA | 7.357552005 | 0.835427445 | 7.212699025 | 0.828835979 |
| novel_pir857 | 0.01 | NA | 1.543333333 | 1.466666667 | NA | NA | 7.269905883 | 0.826804632 | 7.196397213 | 0.827910053 |
| novel_pir865 | 0.01 | NA | 1.513333333 | 1.933333333 | NA | NA | 7.241585987 | 0.825203252 | 7.594946589 | 0.855952381 |
| novel_pir924 | 0.206666667 | NA | 38.77 | 17.74 | NA | NA | 7.55149121 | 0.949494949 | 6.423556485 | 0.891534392 |
| novel_pir935 | 0.01 | NA | 1.75 | 1.776666667 | NA | NA | 7.451211112 | 0.841463415 | 7.473029222 | 0.848941799 |
| novel_pir937 | 0.01 | NA | 1.763333333 | 1.456666667 | NA | NA | 7.462161411 | 0.842202513 | 7.186526969 | 0.827380952 |
| novel_pir942 | 0.01 | NA | 1.643333333 | 2.08 | NA | NA | 7.360481336 | 0.835427445 | 7.700439718 | 0.862830688 |
| novel_pir956 | 0.01 | NA | 1.496666667 | 1.31 | NA | NA | 7.225609134 | 0.823971422 | 7.033423002 | 0.813888889 |
| novel_pir959 | 0.833333333 | NA | 83.36 | 108.4066667 | NA | NA | 6.644317778 | 0.909090909 | 7.023344076 | 0.946957672 |
| novel_pir964 | 0.913333333 | NA | 76.66666667 | 63.76333333 | NA | NA | 6.391314158 | 0.892091648 | 6.125441753 | 0.887962963 |
| novel_pir967 | 0.903333333 | NA | 102.3666667 | 107.4066667 | NA | NA | 6.824271851 | 0.924119241 | 6.893609382 | 0.941269841 |
| novel_pir970 | 0.01 | NA | 2.36 | 2.95 | NA | NA | 7.882643049 | 0.868070953 | 8.204571144 | 0.891137566 |
| novel_pir974 | 0.153333333 | NA | 9.92 | 13.03666667 | NA | NA | 6.015596855 | 0.81867455 | 6.409759865 | 0.883730159 |
| novel_pir983 | 0.14 | NA | 23.96333333 | 22.69666667 | NA | NA | 7.419257966 | 0.935452082 | 7.340909795 | 0.940740741 |
| hsa_piR_000794 | 0.01 | 1.216666667 | NA | NA | 6.926790153 | 0.800523062 | NA | NA | NA | NA |
| novel_pir1042 | 0.01 | 1.266666667 | NA | NA | 6.984893108 | 0.80658583 | NA | NA | NA | NA |
| novel_pir1146 | 0.01 | 1.436666667 | NA | NA | 7.166581558 | 0.822396576 | NA | NA | NA | NA |
| novel_pir1156 | 0.01 | 2.376666667 | NA | NA | 7.892795766 | 0.868402282 | NA | NA | NA | NA |
| novel_pir1172 | 0.01 | 1.44 | NA | NA | 7.169925001 | 0.822396576 | NA | NA | NA | NA |
| novel_pir1173 | 0.01 | 1.973333333 | NA | NA | 7.624490865 | 0.852829291 | NA | NA | NA | NA |
| novel_pir1182 | 0.01 | 1.926666667 | NA | NA | 7.589963182 | 0.850689491 | NA | NA | NA | NA |
| novel_pir1184 | 0.153333333 | 10.74666667 | NA | NA | 6.131074072 | 0.832738944 | NA | NA | NA | NA |
| novel_pir1252 | 0.01 | 3.73 | NA | NA | 8.54303182 | 0.899548264 | NA | NA | NA | NA |
| novel_pir1330 | 0.01 | 1.563333333 | NA | NA | 7.288481612 | 0.832025678 | NA | NA | NA | NA |
| novel_pir1530 | 0.01 | 1.353333333 | NA | NA | 7.080373416 | 0.814907275 | NA | NA | NA | NA |
| novel_pir1533 | 0.01 | 1.47 | NA | NA | 7.199672345 | 0.824417499 | NA | NA | NA | NA |
| novel_pir1625 | 0.01 | 1.736666667 | NA | NA | 7.440177062 | 0.842605801 | NA | NA | NA | NA |
| novel_pir227 | 0.01 | 1.26 | NA | NA | 6.977279923 | 0.805872563 | NA | NA | NA | NA |
| novel_pir286 | 0.01 | 1.543333333 | NA | NA | 7.269905883 | 0.8308369 | NA | NA | NA | NA |
| novel_pir292 | 0.286666667 | 2.146666667 | NA | NA | 7.745954377 | 0.859724204 | NA | NA | NA | NA |
| novel_pir297 | 0.01 | 16.69 | NA | NA | 5.863465985 | 0.806110319 | NA | NA | NA | NA |
| novel_pir303 | 0.01 | 1.906666667 | NA | NA | 7.574908836 | 0.849976224 | NA | NA | NA | NA |
| novel_pir317 | 0.01 | 1.523333333 | NA | NA | 7.251087854 | 0.829053733 | NA | NA | NA | NA |
| novel_pir318 | 0.01 | 1.92 | NA | NA | 7.584962501 | 0.850451736 | NA | NA | NA | NA |
| novel_pir347 | 0.01 | 3.133333333 | NA | NA | 8.291554446 | 0.888373752 | NA | NA | NA | NA |
| novel_pir37 | 0.01 | 2.366666667 | NA | NA | 7.886712714 | 0.867570138 | NA | NA | NA | NA |
| novel_pir382 | 0.01 | 1.47 | NA | NA | 7.199672345 | 0.824417499 | NA | NA | NA | NA |
| novel_pir387 | 0.01 | 2.476666667 | NA | NA | 7.9522559 | 0.872206372 | NA | NA | NA | NA |
| novel_pir407 | 0.01 | 1.983333333 | NA | NA | 7.631783357 | 0.853185925 | NA | NA | NA | NA |
| novel_pir422 | 0.01 | 1.28 | NA | NA | 7 | 0.807299097 | NA | NA | NA | NA |
| novel_pir423 | 0.01 | 1.61 | NA | NA | 7.330916878 | 0.834878745 | NA | NA | NA | NA |
| novel_pir442 | 0.01 | 1.293333333 | NA | NA | 7.014950341 | 0.807536852 | NA | NA | NA | NA |
| novel_pir454 | 0.01 | 2.506666667 | NA | NA | 7.969626351 | 0.87339515 | NA | NA | NA | NA |
| novel_pir455 | 0.01 | 2.513333333 | NA | NA | 7.973458213 | 0.87339515 | NA | NA | NA | NA |
| novel_pir456 | 0.01 | 1.756666667 | NA | NA | 7.456696651 | 0.843319068 | NA | NA | NA | NA |
| novel_pir458 | 0.01 | 1.406666667 | NA | NA | 7.136136688 | 0.820970043 | NA | NA | NA | NA |
| novel_pir459 | 6.903333333 | 1.403333333 | NA | NA | 7.132713922 | 0.820732287 | NA | NA | NA | NA |
| novel_pir463 | 1.073333333 | 390.43 | NA | NA | 5.821626954 | 0.833452211 | NA | NA | NA | NA |
| novel_pir497 | 0.01 | 88.79666667 | NA | NA | 6.370335428 | 0.887779363 | NA | NA | NA | NA |
| novel_pir514 | 0.01 | 1.633333333 | NA | NA | 7.351675438 | 0.836424156 | NA | NA | NA | NA |
| novel_pir549 | 0.01 | 1.923333333 | NA | NA | 7.587465008 | 0.850451736 | NA | NA | NA | NA |
| novel_pir559 | 0.01 | 1.393333333 | NA | NA | 7.122396631 | 0.818235854 | NA | NA | NA | NA |
| novel_pir593 | 0.01 | 1.63 | NA | NA | 7.348728154 | 0.836424156 | NA | NA | NA | NA |
| novel_pir595 | 0.01 | 1.22 | NA | NA | 6.930737338 | 0.800523062 | NA | NA | NA | NA |
| novel_pir601 | 0.233333333 | 1.353333333 | NA | NA | 7.080373416 | 0.814907275 | NA | NA | NA | NA |
| novel_pir602 | 0.01 | 15.72 | NA | NA | 6.074064986 | 0.832144555 | NA | NA | NA | NA |
| novel_pir603 | 0.01 | 1.546666667 | NA | NA | 7.273018494 | 0.831193533 | NA | NA | NA | NA |
| novel_pir604 | 0.26 | 1.54 | NA | NA | 7.266786541 | 0.8308369 | NA | NA | NA | NA |
| novel_pir607 | 0.46 | 20.41333333 | NA | NA | 6.294856349 | 0.859248692 | NA | NA | NA | NA |
| novel_pir61 | 0.01 | 35.86666667 | NA | NA | 6.284866001 | 0.867807893 | NA | NA | NA | NA |
| novel_pir610 | 0.01 | 1.336666667 | NA | NA | 7.062495926 | 0.81359962 | NA | NA | NA | NA |
| novel_pir639 | 0.01 | 4.7 | NA | NA | 8.876516947 | 0.914407989 | NA | NA | NA | NA |
| novel_pir713 | 0.01 | 1.763333333 | NA | NA | 7.462161411 | 0.843319068 | NA | NA | NA | NA |
| novel_pir730 | 0.01 | 1.436666667 | NA | NA | 7.166581558 | 0.822396576 | NA | NA | NA | NA |
| novel_pir732 | 0.01 | 1.986666667 | NA | NA | 7.63420602 | 0.853542558 | NA | NA | NA | NA |
| novel_pir748 | 0.01 | 2.076666667 | NA | NA | 7.698125852 | 0.857584403 | NA | NA | NA | NA |
| novel_pir919 | 0.01 | 1.246666667 | NA | NA | 6.961931959 | 0.803613885 | NA | NA | NA | NA |
| novel_pir1628 | 0.38 | NA | 21.81 | NA |  |  | 5.842846541 | 0.81140675 | NA | NA |
| novel_pir350 | 0.01 | NA | 1.496666667 | NA |  |  | 7.225609134 | 0.823971422 | NA | NA |
| novel_pir1191 | 0.01 | NA | 1.766666667 | NA |  |  | 7.464886049 | 0.842202513 | NA | NA |
| novel_pir1037 | 0.01 | NA | 1.59 | NA |  |  | 7.312882955 | 0.83037694 | NA | NA |
| novel_pir384 | 0.01 | NA | 1.36 | NA |  |  | 7.087462841 | 0.8117763 | NA | NA |
| novel_pir1017 | 0.01 | NA | 2.08 | NA |  |  | 7.700439718 | 0.857723577 | NA | NA |
| novel_pir1014 | 0.01 | NA | 1.5 | NA |  |  | 7.22881869 | 0.823971422 | NA | NA |
| novel_pir1016 | 0.01 | NA | 1.383333333 | NA |  |  | 7.112005026 | 0.814363144 | NA | NA |
| novel_pir176 | 0.01 | NA | 1.346666667 | NA |  |  | 7.073248982 | 0.810790835 | NA | NA |
| novel_pir206 | 0.01 | NA | 1.45 | NA |  |  | 7.17990909 | 0.819413649 | NA | NA |
| novel_pir1735 | 0.01 | NA | 1.426666667 | NA |  |  | 7.156504486 | 0.818058635 | NA | NA |
| novel_pir929 | 0.01 | NA | 1.823333333 | NA |  |  | 7.510434522 | 0.845282089 | NA | NA |
| hsa_piR_001184 | 0.123333333 | NA | 11.40333333 | NA |  |  | 6.530749023 | 0.871643262 | NA | NA |
| novel_pir1462 | 0.01 | NA | 1.616666667 | NA |  |  | 7.336878436 | 0.833456516 | NA | NA |
| novel_pir490 | 0.01 | NA | 3.31 | NA |  |  | 8.370687407 | 0.892461197 | NA | NA |
| novel_pir1365 | 0.01 | NA | 1.366666667 | NA |  |  | 7.094517599 | 0.812269032 | NA | NA |
| novel_pir1034 | 0.01 | NA | 1.273333333 | NA |  |  | 6.992466327 | 0.803030303 | NA | NA |
| novel_pir808 | 0.01 | NA | 1.566666667 | NA |  |  | 7.291554446 | 0.829761025 | NA | NA |
| novel_pir414 | 0.01 | NA | 1.48 | NA |  |  | 7.209453366 | 0.822985957 | NA | NA |
| novel_pir943 | 0.01 | NA | 1.473333333 | NA |  |  | 7.202940059 | 0.82187731 | NA | NA |
| novel_pir1004 | 0.143333333 | NA | 11.24666667 | NA |  |  | 6.293979504 | 0.852673072 | NA | NA |
| novel_pir284 | 0.01 | NA | 2.143333333 | NA |  |  | 7.743712427 | 0.859940872 | NA | NA |
| novel_pir899 | 0.01 | NA | 1.653333333 | NA |  |  | 7.36923381 | 0.835920177 | NA | NA |
| novel_pir923 | 0.01 | NA | 1.613333333 | NA |  |  | 7.333900737 | 0.833086967 | NA | NA |
| novel_pir1406 | 0.01 | NA | 1.3 | NA |  |  | 7.022367813 | 0.805493964 | NA | NA |
| novel_pir1030 | 0.01 | NA | 1.383333333 | NA |  |  | 7.112005026 | 0.814363144 | NA | NA |
| novel_pir541 | 0.01 | NA | 1.26 | NA |  |  | 6.977279923 | 0.801552106 | NA | NA |
| novel_pir565 | 0.01 | NA | 1.343333333 | NA |  |  | 7.069673528 | 0.810298103 | NA | NA |
| novel_pir1039 | 0.01 | NA | 2.07 | NA |  |  | 7.693486957 | 0.856861296 | NA | NA |
| hsa_piR_000823 | 0.333333333 | NA | NA | 38.14 | NA | NA | NA | NA | 6.838195439 | 0.928439153 |
| hsa_piR_004506 | 0.196666667 | NA | NA | 13.9 | NA | NA | NA | NA | 6.143188619 | 0.861243386 |
| hsa_piR_016239 | 0.01 | NA | NA | 1.423333333 | NA | NA | NA | NA | 7.153129759 | 0.823280423 |
| hsa_piR_016677 | 42.58 | NA | NA | 4098.74 | NA | NA | NA | NA | 6.588860717 | 0.934259259 |
| hsa_piR_020365 | 2.39 | NA | NA | 503.62 | NA | NA | NA | NA | 7.719181149 | 0.976455026 |
| novel_pir108 | 0.01 | NA | NA | 1.393333333 | NA | NA | NA | NA | 7.122396631 | 0.820899471 |
| novel_pir1227 | 0.01 | NA | NA | 2.22 | NA | NA | NA | NA | 7.794415866 | 0.867063492 |
| novel_pir1262 | 0.01 | NA | NA | 1.206666667 | NA | NA | NA | NA | 6.914883386 | 0.802910053 |
| novel_pir1347 | 0.01 | NA | NA | 1.886666667 | NA | NA | NA | NA | 7.559695742 | 0.853703704 |
| novel_pir1362 | 0.01 | NA | NA | 2.006666667 | NA | NA | NA | NA | 7.648657176 | 0.86031746 |
| novel_pir1400 | 0.01 | NA | NA | 1.666666667 | NA | NA | NA | NA | 7.380821784 | 0.842063492 |
| novel_pir1403 | 0.01 | NA | NA | 1.606666667 | NA | NA | NA | NA | 7.327926836 | 0.839550265 |
| novel_pir1451 | 0.01 | NA | NA | 3.173333333 | NA | NA | NA | NA | 8.309855263 | 0.89510582 |
| novel_pir1499 | 0.01 | NA | NA | 1.243333333 | NA | NA | NA | NA | 6.95806932 | 0.808333333 |
| novel_pir170 | 0.01 | NA | NA | 1.44 | NA | NA | NA | NA | 7.169925001 | 0.824338624 |
| novel_pir1701 | 0.01 | NA | NA | 1.193333333 | NA | NA | NA | NA | 6.898853277 | 0.801851852 |
| novel_pir1739 | 1.286666667 | NA | NA | 0.01 | NA | NA | NA | NA | -7.007494537 | 0.812037037 |
| novel_pir371 | 0.01 | NA | NA | 1.393333333 | NA | NA | NA | NA | 7.122396631 | 0.820899471 |
| novel_pir373 | 0.01 | NA | NA | 1.906666667 | NA | NA | NA | NA | 7.574908836 | 0.85489418 |
| novel_pir377 | 0.01 | NA | NA | 2.043333333 | NA | NA | NA | NA | 7.674780763 | 0.861375661 |
| novel_pir395 | 0.01 | NA | NA | 1.506666667 | NA | NA | NA | NA | 7.235216462 | 0.832936508 |
| novel_pir397 | 0.01 | NA | NA | 1.333333333 | NA | NA | NA | NA | 7.058893689 | 0.816666667 |
| novel_pir427 | 0.01 | NA | NA | 1.293333333 | NA | NA | NA | NA | 7.014950341 | 0.812037037 |
| novel_pir434 | 0.01 | NA | NA | 1.696666667 | NA | NA | NA | NA | 7.406559345 | 0.844708995 |
| novel_pir470 | 0.01 | NA | NA | 1.396666667 | NA | NA | NA | NA | 7.125843933 | 0.821428571 |
| novel_pir506 | 0.01 | NA | NA | 1.236666667 | NA | NA | NA | NA | 6.950312876 | 0.808333333 |
| novel_pir544 | 0.01 | NA | NA | 1.4 | NA | NA | NA | NA | 7.129283017 | 0.821428571 |
| novel_pir900 | 0.01 | NA | NA | 1.32 | NA | NA | NA | NA | 7.044394119 | 0.816269841 |
| novel_pir957 | 0.01 | NA | NA | 1.996666667 | NA | NA | NA | NA | 7.641449692 | 0.860185185 |
| novel_pir963 | 0.01 | NA | NA | 1.273333333 | NA | NA | NA | NA | 6.992466327 | 0.81005291 |
| novel_pir375 | 0.01 | 3.73 | 1.396666667 | 4.77 | NA | NA | 7.125843933 | 0.81584134 | 8.897845456 | 0.917857143 |
