# Supplementary material for: Trypanosoma cruzi dysregulates expression profile of piRNAs in primary human cardiac fibroblasts during early infection phase
Source: Front Cell Infect Microbiol. 2023 Mar 2;13:1083379. doi: 10.3389/fcimb.2023.1083379 (PMC10017870; doi:10.3389/fcimb.2023.1083379)
Supplement: Supplementary file 2 [file Table_2.docx]

**Supplementary Table 2: Sequences of differentially expressed known and novel piRNAs.**

| **piRNA id** | **Sequence** |
| --- | --- |
| hsa_piR_000753 | AGCAGTTGAACATGGGTCAGTCGGTCCTG |
| hsa_piR_000805 | AGCCTGAGCAACATAGCGAGACCCCGTCTCTA |
| hsa_piR_001356 | CAACAAGTACCGTAAGGGAAAGTTGA |
| hsa_piR_002468 | TCAGAAGATTCCAGGTTCGACTCCTGGC |
| hsa_piR_002485 | TCAGACATTTGGTGTATGTGCTTGGC |
| hsa_piR_004150 | TCCCTGGTAGTCTAGTGGTTAGGATTC |
| hsa_piR_004987 | TCGCCGTGATCGTATAGTGGTTAGTACTCTG |
| hsa_piR_013624 | TGGGAATGCAGCCCAAAGCGGGTGGTA |
| hsa_piR_016240 | TGTTAACCGAAAGGTTGGTGGTTCGAGCC |
| hsa_piR_016742 | CCGGCTAGCTCAGTCGGTAGAGCATGAGA |
| hsa_piR_016828 | CCTGGGCAACATAGCGAGACCTCGTCTC |
| hsa_piR_017716 | TTCCCTGGTGGTCTAGTGGTTAGGATTCGGC |
| hsa_piR_018573 | TTGGTGTATGTGCTTGGCTGAGGAGCC |
| hsa_piR_019825 | GCATTGGTGGTTCAGTGGTAGAATTCTCAC |
| hsa_piR_019949 | GCCTGGATAGCTCAGTTGGTAGAGCATCAGA |
| hsa_piR_020388 | GGCTCGTTGGTCTAGGGGTATGATTCTCGG |
| hsa_piR_020391 | GGCTCTGTTGCGCAATGGATAGCGCAT |
| hsa_piR_020490 | GGGGCGAAGCTACCATCTGTGGGATT |
| hsa_piR_020496 | GGGGGATTAGCTCAAATGGTAGAGCGCTCG |
| hsa_piR_020499 | GGGGGTATAGCTCAGTGGTAGAGCATTTGA |
| hsa_piR_020548 | GGTCAGTCGGTCCTGAGAGATGGGCGAGC |
| hsa_piR_020582 | GGTTCCATGGTGTAATGGTTAGCACTCTG |
| hsa_piR_004506 | TCCTCATTAGTATAGTGGTGAGTATCCC |
| hsa_piR_020365 | GGCCGTGATCGTATAGTGGTTAGTACTCTG |
| hsa_piR_000823 | AGCGTTGGTGGTATAGTGGTGAGCATAGCTGC |
| hsa_piR_016239 | TGTTAACCGAAAGATTGGTGGTTCGAG |
| hsa_piR_016677 | CCCCTGGTGGTCTAGTGGTTAGGATTCGGC |
| hsa_piR_001184 | ATCAGACCCCAGAAAAGGTGTTGGTTGAT |
| hsa_piR_000794 | AGCCCGGCTAGCTCAGTCGGTAGAGCATGAGA |
| novel_pir1000 | GTGTATGTGCTTGGCTGAGG |
| novel_pir1001 | GTGTATGTGCTTGGCTGAGGAGCC |
| novel_pir1002 | GTGTATGTGCTTGGCTGAG |
| novel_pir1003 | GTGTATGTGCTTGGCTGA |
| novel_pir1005 | GTGTATGTGCTTGGCTGAGGAGC |
| novel_pir1006 | TGTATGTGCTTGGCTGAGGAGC |
| novel_pir1007 | TGTATGTGCTTGGCTGAGG |
| novel_pir1009 | TGTATGTGCTTGGCTGAG |
| novel_pir1010 | TGTATGTGCTTGGCTGAGGAG |
| novel_pir1012 | TGTATGTGCTTGGCTGAGGAGCC |
| novel_pir1013 | GTATGTGCTTGGCTGAGGAGCC |
| novel_pir1015 | GTATGTGCTTGGCTGAGGAG |
| novel_pir1018 | GTATGTGCTTGGCTGAGGAGCCA |
| novel_pir1019 | TATGTGCTTGGCTGAGGAGC |
| novel_pir1020 | TATGTGCTTGGCTGAGGAGCCAATGGGG |
| novel_pir1021 | TATGTGCTTGGCTGAGGAG |
| novel_pir1023 | TATGTGCTTGGCTGAGGA |
| novel_pir1026 | TATGTGCTTGGCTGAGGAGCCAA |
| novel_pir1027 | TATGTGCTTGGCTGAGGAGCC |
| novel_pir1028 | TATGTGCTTGGCTGAGGAGCCA |
| novel_pir1029 | ATGTGCTTGGCTGAGGAGCCA |
| novel_pir1032 | ATGTGCTTGGCTGAGGAGCC |
| novel_pir1033 | ATGTGCTTGGCTGAGGAG |
| novel_pir1043 | TCGCAGTCTCCCTTGGAGGCGTGGG |
| novel_pir1045 | TCGCAGTCTCCCTTGGAGGCGTG |
| novel_pir1061 | CTTTGTGAAGGGCAGGGCGCCCTGG |
| novel_pir1063 | CTTTGTGAAGGGCAGGGCGCCC |
| novel_pir1065 | TTTGTGAAGGGCAGGGCG |
| novel_pir1075 | GTGAGTGTGTGTGTGTGAGTGTG |
| novel_pir1098 | AATGTGGCGTACGGAAGACCC |
| novel_pir1099 | AATGTGGCGTACGGAAGA |
| novel_pir110 | TCCTCCTGGCTGGCTCGCCA |
| novel_pir1100 | AATGTGGCGTACGGAAGAC |
| novel_pir1101 | AATGTGGCGTACGGAAGACC |
| novel_pir1102 | ATGTGGCGTACGGAAGAC |
| novel_pir1103 | ATGTGGCGTACGGAAGACC |
| novel_pir1104 | ATGTGGCGTACGGAAGACCC |
| novel_pir1111 | GCCCAAGTCCTTCTGATCGAGG |
| novel_pir1113 | CCCAAGTCCTTCTGATCG |
| novel_pir1114 | CCCAAGTCCTTCTGATCGAG |
| novel_pir1115 | CCCAAGTCCTTCTGATCGAGG |
| novel_pir1116 | CCCAAGTCCTTCTGATCGA |
| novel_pir1117 | CCAAGTCCTTCTGATCGAGG |
| novel_pir1118 | CAAGTCCTTCTGATCGAGG |
| novel_pir1119 | CAAGTCCTTCTGATCGAG |
| novel_pir112 | TTCCTCCTGGCTGGCTCG |
| novel_pir1120 | AAGTCCTTCTGATCGAGG |
| novel_pir1121 | GGAAATGTGGCGTACGGAA |
| novel_pir1122 | GGAAATGTGGCGTACGGA |
| novel_pir1181 | TTACCTCCTCATGCCGGACT |
| novel_pir1183 | TTACCTCCTCATGCCGGA |
| novel_pir1217 | CAGAAACCTCCCGTGGAG |
| novel_pir122 | GTGGTCTAGTGGCTAGGAT |
| novel_pir1222 | CTTTTGGGTTTTAAGCAGG |
| novel_pir1223 | CTTTTGGGTTTTAAGCAGGAGGT |
| novel_pir1229 | TTGGGTTTTAAGCAGGAGGTG |
| novel_pir1231 | TTGGGTTTTAAGCAGGAGGT |
| novel_pir1244 | AATGGTTGGAAAAGTTCA |
| novel_pir1249 | TGTGTCCTCTGCTGTTTCT |
| novel_pir1257 | GCCTGGATAGCTCAGTCGGTAGAGCA |
| novel_pir1259 | GCCTGGATAGCTCAGTCGGTAGAGC |
| novel_pir1263 | GGATAGCTCAGTCGGTAGAG |
| novel_pir1264 | GATAGCTCAGTCGGTAGAG |
| novel_pir1267 | ATAGCTCAGTCGGTAGAG |
| novel_pir127 | TCCCTGGTGGTCTAGTGGCTAGGA |
| novel_pir1275 | CTAAGCAGGGTCGGGCCAGG |
| novel_pir128 | GTGGTCTAGTGGCTAGGA |
| novel_pir1283 | TGGTGTAATGGTTAGCACTCTG |
| novel_pir1290 | GGTGTAATGGTTAGCACTCTG |
| novel_pir1292 | GGTGTAATGGTTAGCACTCTGG |
| novel_pir1293 | GTGTAATGGTTAGCACTCTG |
| novel_pir1294 | GTGTAATGGTTAGCACTCTGG |
| novel_pir1302 | TGTAATGGTTAGCACTCTGG |
| novel_pir1303 | TGTAATGGTTAGCACTCTG |
| novel_pir1324 | TTTGTAGTTGATTGAATCT |
| novel_pir1328 | TACTTGTTGGATTGTTGA |
| novel_pir133 | GGTGGTCTAGTGGCTAGG |
| novel_pir1334 | TCTGTTATTGATCTTGGCA |
| novel_pir1338 | TCAGGTTCAGGAGCAGGT |
| novel_pir134 | TCCCTGGTGGTCTAGTGGCTAG |
| novel_pir1351 | CTAAGCAGGGTCGGGCCTG |
| novel_pir1352 | CTAAGCAGGGTCGGGCCT |
| novel_pir1394 | TCTTTGTTCTTGTGTTGCT |
| novel_pir1396 | GGTATAGTGGTGAGCATA |
| novel_pir1397 | TGGTATAGTGGTGAGCATA |
| novel_pir1398 | GTGGTATAGTGGTGAGCATA |
| novel_pir1411 | CTCTCTCTCTCTCTCCCCCGCT |
| novel_pir1429 | ACTTGGTCTCTTGTTTGT |
| novel_pir1441 | CTACTTCGTGGCATCTAACCATCGTTTT |
| novel_pir1445 | CTACTTCGTGGCATCTAACCATCGTTT |
| novel_pir1468 | CAGCTGGGGATTGTGGGT |
| novel_pir149 | TCGGATCCCACTTCTGAC |
| novel_pir1496 | GTGGTCTAGTGGTTAGGATTC |
| novel_pir1497 | GTGGTCTAGTGGTTAGGATT |
| novel_pir1498 | GGTCTAGTGGTTAGGATT |
| novel_pir1500 | GTGGTCTAGTGGTTAGGAT |
| novel_pir1501 | GTGGTCTAGTGGTTAGGA |
| novel_pir1516 | CTGGATTGTGGGGTAAGC |
| novel_pir1569 | ACTGAAGATCTAAAGGTCCCTGGT |
| novel_pir1572 | GACTGAAGATCTAAAGGTCCCTGG |
| novel_pir1573 | ACTGAAGATCTAAAGGTCCCTGG |
| novel_pir1574 | GACTGAAGATCTAAAGGTCCCTG |
| novel_pir1575 | ACTGAAGATCTAAAGGTCCCTG |
| novel_pir1577 | ACTGAAGATCTAAAGGTCCCT |
| novel_pir1578 | GACTGAAGATCTAAAGGTCCC |
| novel_pir1579 | ACTGAAGATCTAAAGGTCCC |
| novel_pir1581 | ACTGAAGATCTAAAGGTCC |
| novel_pir1583 | ACTGAAGATCTAAAGGTC |
| novel_pir1597 | GATTCCGTGGGTGGTGGTGC |
| novel_pir1598 | ATTCCGTGGGTGGTGGTGC |
| novel_pir1600 | GATTCCGTGGGTGGTGGTG |
| novel_pir1601 | ATTCCGTGGGTGGTGGTG |
| novel_pir1602 | GATTCCGTGGGTGGTGGT |
| novel_pir1603 | TGTGCCTAAAATAATTCCT |
| novel_pir1627 | TTACAAAGGAACAGTTGGC |
| novel_pir1635 | CCTATTCAGTATGGTTGT |
| novel_pir166 | CTGACCCGGTGAGGCGGGGGGG |
| novel_pir167 | CACTGACCCGGTGAGGCGGGGGGG |
| novel_pir1677 | ATGTTGTTCTACTTTTTGT |
| novel_pir168 | ACTGACCCGGTGAGGCGGGGGGG |
| novel_pir1685 | GTACGTAGCAGAGCAGCTCCCTCGCTG |
| novel_pir1686 | GTACGTAGCAGAGCAGCTCCCTCGCT |
| novel_pir1688 | TACGTAGCAGAGCAGCTCCCTCGCT |
| novel_pir1694 | ACGTAGCAGAGCAGCTCCCTCGCTG |
| novel_pir1695 | CGTAGCAGAGCAGCTCCCTCGCTG |
| novel_pir1698 | CGTAGCAGAGCAGCTCCCTCGCT |
| novel_pir17 | GGCTGAGGTGGGAGGATC |
| novel_pir173 | CACTGACCCGGTGAGGCGGGG |
| novel_pir1703 | ATCTATTGAAAGTCAGCCCT |
| novel_pir1704 | ATCTATTGAAAGTCAGCC |
| novel_pir1705 | ATCTATTGAAAGTCAGCCC |
| novel_pir1707 | TCTATTGAAAGTCAGCCCT |
| novel_pir1713 | ATGATGTGTTGTTGCCAT |
| novel_pir1715 | CATGCCTCAGAATCACTG |
| novel_pir186 | ATTTTTTGAGAGTTTGAT |
| novel_pir197 | TGAGTGTGTGTGTGAGTGTGTG |
| novel_pir205 | CTCCCCGCGCCCCCGCCCC |
| novel_pir212 | CATCTGAATTATTGCTTGA |
| novel_pir221 | TCTACGGCCATACCACCCT |
| novel_pir230 | TTTTTAATAGGCCACAGA |
| novel_pir241 | CATCTTGTCATTTACCTCT |
| novel_pir248 | TTGGGTTTTAAGCAGGAGG |
| novel_pir256 | AGTGGTTAAGGCAATGGA |
| novel_pir281 | AATGCTGCTGGAGTAATTGG |
| novel_pir290 | CAGGTCAAAACTCCCGTGCTGATC |
| novel_pir291 | TCAAAACTCCCGTGCTGATC |
| novel_pir293 | GTCAAAACTCCCGTGCTGATC |
| novel_pir296 | AAAACTCCCGTGCTGATC |
| novel_pir299 | CAGGTCAAAACTCCCGTGCTGA |
| novel_pir300 | CAGGTCAAAACTCCCGTGCTG |
| novel_pir310 | GGGTGAACCGGCCCAGGTCGGA |
| novel_pir316 | CCTAAGGAGGGGTGAACCGGCCCAG |
| novel_pir319 | CCTAAGGAGGGGTGAACCGGCCCA |
| novel_pir320 | CCTAAGGAGGGGTGAACCGGCCC |
| novel_pir324 | GCCTAAGGAGGGGTGAACCGGCCC |
| novel_pir327 | AAGGAGGGGTGAACCGGCC |
| novel_pir329 | GCCTAAGGAGGGGTGAACCGGCC |
| novel_pir330 | CTAAGGAGGGGTGAACCGGCC |
| novel_pir331 | CCTAAGGAGGGGTGAACCGGCC |
| novel_pir332 | TAAGGAGGGGTGAACCGGCC |
| novel_pir339 | CCAGGTTGCCTAAGGAGGGGTG |
| novel_pir349 | GGGAGCGGGGGACCACCAGGTTG |
| novel_pir35 | AAAGCAATGGAAAGTGGA |
| novel_pir351 | CCGGGAGCGGGGGACCACCAGGTTG |
| novel_pir357 | AATATGGTGACCTCCCGGGAGCGGGGGACCACC |
| novel_pir358 | ATATGGTGACCTCCCGGGAGCGGGGGACCACC |
| novel_pir359 | CAATATGGTGACCTCCCGGGAGCGGGGGACCACC |
| novel_pir372 | AATATGGTGACCTCCCGGGAGCGGGGGA |
| novel_pir381 | ACTAAGTTCGGCATCAATATGGTGACCTCC |
| novel_pir383 | AGTTCGGCATCAATATGGTGACCTCC |
| novel_pir386 | CACTAAGTTCGGCATCAATATGGTGACCTCC |
| novel_pir388 | CACTAAGTTCGGCATCAATATGGTGACCTC |
| novel_pir390 | ACTAAGTTCGGCATCAATATGGTGACCTC |
| novel_pir399 | CACTAAGTTCGGCATCAATATGGTG |
| novel_pir400 | AGTTCGGCATCAATATGGTG |
| novel_pir405 | AGTTCGGCATCAATATGGT |
| novel_pir406 | CACTAAGTTCGGCATCAATATGGT |
| novel_pir413 | CACTAAGTTCGGCATCAAT |
| novel_pir415 | CCGATCGGGTGTCCGCACTAAGTTCGGCATCA |
| novel_pir420 | CCGATCGGGTGTCCGCACTAAGTTCGGCATC |
| novel_pir426 | ATCGGGTGTCCGCACTAAGTTCGGCA |
| novel_pir429 | CCGATCGGGTGTCCGCACTAAGTTCGGCA |
| novel_pir432 | CCGATCGGGTGTCCGCACTAAGTTCGGC |
| novel_pir435 | ATCGGGTGTCCGCACTAAGTTCGGC |
| novel_pir436 | CCGATCGGGTGTCCGCACTAAGTTCGG |
| novel_pir438 | TGCCGATCGGGTGTCCGCACTAAGTTCGG |
| novel_pir439 | CGGGTGTCCGCACTAAGTTCGG |
| novel_pir445 | GCCGATCGGGTGTCCGCACTAAGTTCG |
| novel_pir449 | TGCCGATCGGGTGTCCGCACTAAGTTC |
| novel_pir450 | TGCCGATCGGGTGTCCGCACTAAGTT |
| novel_pir457 | TGCCGATCGGGTGTCCGCACTA |
| novel_pir462 | CGCTATGCCGATCGGGTGTCCGCAC |
| novel_pir464 | TGCCGATCGGGTGTCCGCA |
| novel_pir465 | CGCTATGCCGATCGGGTGTCCGCA |
| novel_pir467 | TGCCGATCGGGTGTCCGC |
| novel_pir468 | CGCTATGCCGATCGGGTGTCCGC |
| novel_pir471 | TGCGCTATGCCGATCGGGTGTCCG |
| novel_pir472 | CGCTATGCCGATCGGGTGTCCG |
| novel_pir476 | CGCTATGCCGATCGGGTGTCC |
| novel_pir479 | CGCTATGCCGATCGGGTGTC |
| novel_pir481 | CGCTATGCCGATCGGGTGT |
| novel_pir504 | AGTTCTGGGCTGTAGTGCGCTATG |
| novel_pir507 | GAGTTCTGGGCTGTAGTGCGCTATG |
| novel_pir508 | TCTGGGCTGTAGTGCGCTATG |
| novel_pir509 | AGGAGTTCTGGGCTGTAGTGCGCTATG |
| novel_pir51 | ACTCAATTTCTGGTCTCC |
| novel_pir512 | AGTTCTGGGCTGTAGTGCGCTAT |
| novel_pir518 | AGTTCTGGGCTGTAGTGCGCTA |
| novel_pir519 | GGAGTTCTGGGCTGTAGTGCGCTA |
| novel_pir522 | AGGAGTTCTGGGCTGTAGTGCGCTA |
| novel_pir523 | GTTCTGGGCTGTAGTGCGCTA |
| novel_pir525 | GAGTTCTGGGCTGTAGTGCGCTA |
| novel_pir528 | AGTTCTGGGCTGTAGTGCGCT |
| novel_pir529 | GAGTTCTGGGCTGTAGTGCGCT |
| novel_pir533 | AGTTCTGGGCTGTAGTGCGC |
| novel_pir536 | AGTTCTGGGCTGTAGTGCG |
| novel_pir537 | GGAGTTCTGGGCTGTAGTGCG |
| novel_pir538 | GAGTTCTGGGCTGTAGTGCG |
| novel_pir540 | GAGTTCTGGGCTGTAGTGC |
| novel_pir542 | AGGAGTTCTGGGCTGTAGTG |
| novel_pir545 | AGGATCGCTTGAGCCCAGGAGTTCTGGG |
| novel_pir566 | AGGTGGGAGGATCGCTTGAGCCCAGG |
| novel_pir569 | GGAGGATCGCTTGAGCCCAGG |
| novel_pir573 | AGGTGGGAGGATCGCTTGAGCCCAG |
| novel_pir579 | GGAGGATCGCTTGAGCCCAG |
| novel_pir580 | AGGTGGGAGGATCGCTTGAGCCCA |
| novel_pir581 | GTGGGAGGATCGCTTGAGCCCA |
| novel_pir583 | TGGGAGGATCGCTTGAGCCCA |
| novel_pir586 | GGAGGATCGCTTGAGCCCA |
| novel_pir587 | AGGTGGGAGGATCGCTTGAGCCC |
| novel_pir590 | AGGTGGGAGGATCGCTTGAGCC |
| novel_pir605 | GCTGAGGTGGGAGGATCG |
| novel_pir606 | AGGCTGAGGTGGGAGGATCG |
| novel_pir614 | CGCGTGCCTGTAGTCCCAGCTACTCGGGA |
| novel_pir615 | GCGCGTGCCTGTAGTCCCAGCTACTCGGGA |
| novel_pir647 | GGTTTTAAGCAGGAGGTG |
| novel_pir655 | ATTGGAGGGCAAGTCTGGTG |
| novel_pir656 | TTGGAGGGCAAGTCTGGTG |
| novel_pir658 | ATTGGAGGGCAAGTCTGGT |
| novel_pir659 | TTGGAGGGCAAGTCTGGT |
| novel_pir660 | ATTGGAGGGCAAGTCTGG |
| novel_pir665 | CACTGCTTCACTTGACTAGCC |
| novel_pir68 | ATTGTGAAGCAGAATTCA |
| novel_pir686 | TAGTGGTATCATGCAAGATTCC |
| novel_pir706 | CTTGATCCTGAAGCAGCT |
| novel_pir715 | GGCCATACCACCCTGAACGCG |
| novel_pir718 | GCCATACCACCCTGAACGCG |
| novel_pir719 | CCATACCACCCTGAACGCG |
| novel_pir720 | CCATACCACCCTGAACGCGC |
| novel_pir722 | CCATACCACCCTGAACGCGCCCGATC |
| novel_pir723 | CCATACCACCCTGAACGCGCCCGAT |
| novel_pir725 | CCATACCACCCTGAACGCGCCCG |
| novel_pir727 | CCATACCACCCTGAACGCGCC |
| novel_pir731 | CATACCACCCTGAACGCGCCCGAT |
| novel_pir734 | CATACCACCCTGAACGCGC |
| novel_pir735 | CATACCACCCTGAACGCGCCCG |
| novel_pir739 | ATACCACCCTGAACGCGCCCG |
| novel_pir741 | ATACCACCCTGAACGCGCC |
| novel_pir744 | ATACCACCCTGAACGCGCCCGAT |
| novel_pir749 | TACCACCCTGAACGCGCCCGAT |
| novel_pir788 | CAGGGGTAGAGCACTGGT |
| novel_pir810 | TAGTGGTTATCACATTCG |
| novel_pir818 | GTTTTCATCCATGGATTCA |
| novel_pir822 | TATCTTGCTTTATTTTCTTCA |
| novel_pir855 | GTTTGTGTGTTGTTGGCAG |
| novel_pir856 | TTTGTGTGTTGTTGGCAG |
| novel_pir857 | GTTTGTGTGTTGTTGGCA |
| novel_pir858 | CAGAAGACTTATTGCTGTTGT |
| novel_pir865 | CTTGCCCAGGAAACCTTTCC |
| novel_pir873 | GTGGTCTAGTGGCTAGGATT |
| novel_pir918 | TTCAAATCCTGTCTTCTTC |
| novel_pir921 | TAAGATATGGTTGCCTTT |
| novel_pir924 | TGATTTCTGCCCAGTGCT |
| novel_pir934 | AGACATTTGGTGTATGTGCTTG |
| novel_pir935 | AGACATTTGGTGTATGTGCTTGGCTG |
| novel_pir937 | AGACATTTGGTGTATGTGCTTGG |
| novel_pir942 | GACATTTGGTGTATGTGCTTGGC |
| novel_pir946 | GACATTTGGTGTATGTGCTTG |
| novel_pir950 | CATTTGGTGTATGTGCTTG |
| novel_pir952 | CATTTGGTGTATGTGCTTGGC |
| novel_pir954 | CATTTGGTGTATGTGCTTGG |
| novel_pir955 | ATTTGGTGTATGTGCTTGGCTG |
| novel_pir956 | ATTTGGTGTATGTGCTTGGCTGAGGA |
| novel_pir959 | ATTTGGTGTATGTGCTTGGC |
| novel_pir960 | ATTTGGTGTATGTGCTTG |
| novel_pir962 | ATTTGGTGTATGTGCTTGGCTGAGGAGCC |
| novel_pir964 | ATTTGGTGTATGTGCTTGG |
| novel_pir967 | TTTGGTGTATGTGCTTGGCTG |
| novel_pir969 | TTTGGTGTATGTGCTTGG |
| novel_pir970 | TTTGGTGTATGTGCTTGGCTGAGGAG |
| novel_pir971 | TTTGGTGTATGTGCTTGGCTGAGGAGCCA |
| novel_pir972 | TTTGGTGTATGTGCTTGGCTGA |
| novel_pir973 | TTTGGTGTATGTGCTTGGC |
| novel_pir974 | TTTGGTGTATGTGCTTGGCT |
| novel_pir976 | TTGGTGTATGTGCTTGGCT |
| novel_pir977 | TTGGTGTATGTGCTTGGC |
| novel_pir978 | TTGGTGTATGTGCTTGGCTGAGG |
| novel_pir980 | TTGGTGTATGTGCTTGGCTGAGGAG |
| novel_pir981 | TTGGTGTATGTGCTTGGCTGA |
| novel_pir982 | TTGGTGTATGTGCTTGGCTGAGGAGCCAA |
| novel_pir983 | TTGGTGTATGTGCTTGGCTGAG |
| novel_pir984 | TTGGTGTATGTGCTTGGCTG |
| novel_pir996 | GGTGTATGTGCTTGGCTG |
| novel_pir998 | GTGTATGTGCTTGGCTGAGGAGCCA |
| novel_pir999 | GTGTATGTGCTTGGCTGAGGAG |
| novel_pir37 | TGGCAGGGGAGATACCATG |
| novel_pir61 | AATCCGAGTCACGGCACCA |
| novel_pir108 | TTCCTCCTGGCTGGCTCGCCA |
| novel_pir170 | CACTGACCCGGTGAGGCGGGGG |
| novel_pir176 | CACTGACCCGGTGAGGCGG |
| novel_pir206 | TCCCCGCGCCCCCGCCCC |
| novel_pir227 | ACGGCCATACCACCCTGAA |
| novel_pir284 | ATGCTGCTGGAGTAATTGG |
| novel_pir286 | AAACTCCCGTGCTGATCAG |
| novel_pir292 | CAAAACTCCCGTGCTGATC |
| novel_pir297 | CAGGTCAAAACTCCCGTGCTGAT |
| novel_pir303 | AGGTCAAAACTCCCGTGCTG |
| novel_pir317 | GCCTAAGGAGGGGTGAACCGGCCCAG |
| novel_pir318 | GCCTAAGGAGGGGTGAACCGGCCCA |
| novel_pir347 | ACCTCCCGGGAGCGGGGGACCACCAGGTTG |
| novel_pir350 | CGGGGGACCACCAGGTTG |
| novel_pir371 | CAATATGGTGACCTCCCGGGAGCGGGGGA |
| novel_pir373 | ATATGGTGACCTCCCGGGAGCGGGGGA |
| novel_pir375 | ATATGGTGACCTCCCGGGAGCGGGGG |
| novel_pir377 | ATATGGTGACCTCCCGGGAGCGGG |
| novel_pir382 | TAAGTTCGGCATCAATATGGTGACCTCC |
| novel_pir384 | ATCAATATGGTGACCTCC |
| novel_pir387 | AAGTTCGGCATCAATATGGTGACCTCC |
| novel_pir395 | CACTAAGTTCGGCATCAATATGGTGA |
| novel_pir397 | AGTTCGGCATCAATATGGTGA |
| novel_pir407 | CACTAAGTTCGGCATCAATATGG |
| novel_pir414 | CACTAAGTTCGGCATCAA |
| novel_pir422 | ATCGGGTGTCCGCACTAAGTTCGGCAT |
| novel_pir423 | CCGATCGGGTGTCCGCACTAAGTTCGGCAT |
| novel_pir427 | GGTGTCCGCACTAAGTTCGGCA |
| novel_pir434 | TGCCGATCGGGTGTCCGCACTAAGTTCGGC |
| novel_pir442 | CGATCGGGTGTCCGCACTAAGTTCGG |
| novel_pir454 | TGCCGATCGGGTGTCCGCACTAAG |
| novel_pir455 | TGCCGATCGGGTGTCCGCACTAA |
| novel_pir456 | CGATCGGGTGTCCGCACTA |
| novel_pir458 | CGCTATGCCGATCGGGTGTCCGCACTA |
| novel_pir459 | TGCCGATCGGGTGTCCGCACT |
| novel_pir463 | TGCCGATCGGGTGTCCGCAC |
| novel_pir470 | GCTATGCCGATCGGGTGTCCG |
| novel_pir490 | CGCTATGCCGATCGGGTG |
| novel_pir497 | AGTTCTGGGCTGTAGTGCGCTATGCCG |
| novel_pir506 | GTTCTGGGCTGTAGTGCGCTATG |
| novel_pir514 | GGAGTTCTGGGCTGTAGTGCGCTAT |
| novel_pir541 | AGTTCTGGGCTGTAGTGC |
| novel_pir544 | AGGAGTTCTGGGCTGTAG |
| novel_pir549 | CTTGAGCCCAGGAGTTCTG |
| novel_pir559 | GATCGCTTGAGCCCAGGAG |
| novel_pir565 | GGATCGCTTGAGCCCAGG |
| novel_pir593 | GGCTGAGGTGGGAGGATCGCTTGAGC |
| novel_pir595 | GGCTGAGGTGGGAGGATCGCTTGAG |
| novel_pir601 | GGCTGAGGTGGGAGGATCGCT |
| novel_pir602 | AGGCTGAGGTGGGAGGATCGCT |
| novel_pir603 | GCTGAGGTGGGAGGATCGC |
| novel_pir604 | GGCTGAGGTGGGAGGATCGC |
| novel_pir607 | GGCTGAGGTGGGAGGATCG |
| novel_pir610 | CTGTAGTCCCAGCTACTCGGGAGGCTG |
| novel_pir639 | GCGCGTGCCTGTAGTCCC |
| novel_pir713 | ACGGCCATACCACCCTGAACGCG |
| novel_pir730 | CATACCACCCTGAACGCGCCCGATCT |
| novel_pir732 | CATACCACCCTGAACGCGCCCGA |
| novel_pir748 | TACCACCCTGAACGCGCCCG |
| novel_pir808 | TCTTGGTTTTTGTCTTCTT |
| novel_pir899 | CTTTGGGTGCTAATGGTG |
| novel_pir900 | TTGATGTTGTTGAGTTGT |
| novel_pir919 | TCAAATCCTGTCTTCTTC |
| novel_pir923 | AGATTAGTTAATAATATTGT |
| novel_pir929 | CAGACATTTGGTGTATGTGCTTGG |
| novel_pir943 | GACATTTGGTGTATGTGCTTGG |
| novel_pir957 | ATTTGGTGTATGTGCTTGGCTGAGGAG |
| novel_pir963 | ATTTGGTGTATGTGCTTGGCTGA |
| novel_pir1004 | GTGTATGTGCTTGGCTGAGGA |
| novel_pir1014 | GTATGTGCTTGGCTGAGGAGC |
| novel_pir1016 | GTATGTGCTTGGCTGAGG |
| novel_pir1017 | GTATGTGCTTGGCTGAGGA |
| novel_pir1030 | ATGTGCTTGGCTGAGGAGC |
| novel_pir1034 | TGTGCTTGGCTGAGGAGCC |
| novel_pir1037 | TGTGCTTGGCTGAGGAGCCA |
| novel_pir1039 | GCTGAGGAGCCAATGGGG |
| novel_pir1042 | AATTGGTTAGAGCTGGTG |
| novel_pir1146 | CTCAGCAGGGTCGGGCCTGGTT |
| novel_pir1156 | GGGTCGGAGTTAGCTCAAGCGG |
| novel_pir1172 | CTCAAGCGGTTACCTCCTCATGCCGG |
| novel_pir1173 | CTCAAGCGGTTACCTCCTCATGCCGGA |
| novel_pir1182 | TTACCTCCTCATGCCGGACTT |
| novel_pir1184 | TTACCTCCTCATGCCGGAC |
| novel_pir1191 | TACCTCCTCATGCCGGAC |
| novel_pir1227 | TTTTGGGTTTTAAGCAGGAGGTG |
| novel_pir1233 | TGGGTTTTAAGCAGGAGGTG |
| novel_pir1252 | CTTTAGCTCAGCGGTTACT |
| novel_pir1262 | TGGATAGCTCAGTCGGTAGAG |
| novel_pir1330 | GAAGCTAAGCAGGGTCAGG |
| novel_pir1347 | CTTTGAAGAGAGAGTTCAA |
| novel_pir1362 | TCCCTGTTGTCTAGTGGTTAGG |
| novel_pir1365 | GCGGGGCCGGGGGTGGGG |
| novel_pir1400 | GTGGTATAGTGGTGAGCAT |
| novel_pir1403 | GTGGTATAGTGGTGAGCA |
| novel_pir1406 | TTGGTGGTATAGTGGTGAG |
| novel_pir1451 | CTACTTCGTGGCATCTAACCATCGTT |
| novel_pir1462 | AAGGGAACGGACTTGAAG |
| novel_pir1499 | TGGTCTAGTGGTTAGGATT |
| novel_pir1530 | GCTGAGGCTGGAGGATCGCT |
| novel_pir1533 | GCTGAGGCTGGAGGATCGC |
| novel_pir1625 | CTAAGCAGGGTTGGGCCTGGTT |
| novel_pir1628 | TTACAAAGGAACAGTTGG |
| novel_pir1701 | TAGCAGAGCAGCTCCCTCGCT |
| novel_pir1735 | GAATCAATAGTTTGTTGCT |
| novel_pir1739 | GGCCTCATTAAATGTTTGTTG |
